# Supplementary material for: Signal-noise metrics for RNA binding protein identification reveal broad spectrum protein-RNA interaction frequencies and dynamics
Source: Nat Commun. 2023 Sep 21;14:5868. doi: 10.1038/s41467-023-41284-9 (PMC10514315; doi:10.1038/s41467-023-41284-9)
Supplement: Supplementary file 1 — Supplementary Information [file 41467_2023_41284_MOESM1_ESM.pdf]

Supplementary Information

Signal-noise metrics for RNA binding protein identification reveal broad spectrum protein-RNA interaction frequencies and dynamics

JohnCarlo Kristofich[1] and Christopher V. Nicchitta[1]*

[1]Department of Cell Biology
Duke University School of Medicine
Durham, North Carolina 27710 USA

*Email: christopher.nicchitta@duke.edu

Supplementary Methods

1. Description of sample types.
2. Description of terminologies.
3. Description of quantitative metrics.
4. Evaluating UV-dependent enrichment and S/N by SDS-PAGE RNase-sensitivity Assay (SRA).
5. Estimating RBP-specific UV-crosslinking efficiencies and S/N ratios by SDS-PAGE and immunoblot.
6. Evaluating total protein and total RNA-bound protein abundance by SDS-PAGE.
7. MS data analysis.
8. RCS rank analysis.
9. Additional protocol information.
9a. Repeated guanidinium thiocyanate-phenol-chloroform extraction (AGPC) protocol.
9b. Isolation of RNP fractions by LEAP-RBP.
9c. LEAP-RBP DNA depletion step.
9d. SDS-PAGE, SYBR Safe, Coomassie Blue, and Silver Stain staining.
9e. Immunoblot.
9f. Sample preparation for MS proteomic analysis.

Supplementary Notes

Supplementary Notes provide additional observations and rationale for successful applications of the methods herein, characterization of S/N, and S/N-based analyses.

Supplementary Note 1 provides supporting information and technical considerations for LEAP-RBP and DNA depletion steps (Supplementary Fig. 1-11).

Supplementary Note 2 provides supporting information and technical considerations for the SDS-PAGE RNase-sensitivity Assay (Supplementary Fig. 12).

Supplementary Note 3 provides supporting information for estimating RNA, protein, and RBP-specific UV-crosslinking efficiencies (Supplementary Fig. 13).

Supplementary Note 4 provides additional observations and rationale for distinguishing UV-dependent enrichment of free protein and signal-dependent recovery of noise (Supplementary Fig. 14, 15) and supporting documentation illustrating the importance of method robustness and specificity for comparative studies (Supplementary Fig. 16).

Supplementary Note 5 provides supporting information and rationale for protein-specific S/N ratios, SILAC LC-MS/MS, and MS data handling (Supplementary Fig. 17-19).

Supplementary Note 6 provides supporting information for S/N-based analyses and additional considerations for comparative LEAP-RBP experiments and other downstream applications (Supplementary Fig. 20, 21).

Supplementary Note 7 provides comparative analysis of proteins identified as UV-enriched* by LEAP-RBP and reference RNA-centric methods (Supplementary Fig. 22-29).

Supplementary Note 8 provides extended S/N-based analysis of LEAP-RBP, INP, and referenced RNA-centric methods (Supplementary Fig. 30-42).

Supplementary Note 9 includes extended protocols details for referenced RNA-centric methods.

Supplementary Methods

1. Description of sample types.

"UV-crosslinked cells" and "non-crosslinked cells" refers to UV-irradiated or non-irradiated samples containing "total cellular mass" (i.e., "total cellular protein", "total cellular RNA", "total cellular DNA", etc.). "UV-crosslinked samples" and "non-crosslinked samples", contained or were derived from UV-crosslinked or non-crosslinked cells. "AGP suspensions" contained samples suspended in acidic guanidinium thiocyanate-phenol (2:1) buffer. In this study, "AGP input suspensions" refers to AGP suspensions containing UV-crosslinked or non-crosslinked cells (i.e., total cellular mass). However, it was considered reasonable for AGP input suspensions to represent any "starting sample" resuspended and/or mixed with >6 parts acidic guanidinium thiocyanate-phenol (2:1) buffer (e.g., cytosolic fractions; Supplementary Note 1). "Aqueous phases" and "organic phases" refers to the upper (aqueous) and lower (organic) phases during AGPC extraction. "AGPC interphase" refers to the insoluble material remaining after AGPC extraction and removal of the aqueous and organic phases. "AGPC interphase samples" contained protein recovered from the AGPC interphase by methanol precipitation (95% v/v). "AGP interphase suspensions" contained the AGPC interphase resuspended in fresh acidic guanidinium thiocyanate-phenol (2:1) buffer. "Final AGPC interphase" refers to the AGPC interphase at maximum $\%TP_S$ (Supplementary Note 4e). "Final AGPC interphase suspensions" contained the final AGPC interphase resuspended in acidic guanidinium thiocyanate-phenol (2:1) buffer. "AGPC mixtures" or "AGPC samples" refers to samples during AGPC extraction prior to centrifugation, or AGP suspensions after the addition of chloroform during the LEAP step. "RNA samples" contained RNA isolated by any process (with or without DNA depletion step). "Protein samples" contained protein isolated by any process. "RNP samples" contained mixtures of crosslinked ribonucleoproteins ("clRNPs") and/or free RNA and protein isolated by any process but excluded samples where DNA contamination impacted RNA (UV-spectrophotometry) quantitation >10%. "Input" refers to samples containing total protein isolated by methanol precipitation (95% v/v) of AGP input suspensions. "clRNP fractions" refers to LEAP-RBP fractions isolated from final AGPC interphase suspensions of UV-crosslinked samples (with or without DNA depletion step). For simplicity, clRNP fractions were labeled as RNP fractions when compared to other RNP fractions (e.g., Fig. 8a-c). Where appropriate, samples or sets of samples were described by the isolation process with or without the word "fraction(s)" (e.g., LEAP-RBP fractions).

2. Description of terminologies.

"Signal" (quantity = S) refers to "RNA-bound proteins" while "noise" (quantity = N) refers to their "unbound counterparts" or "unbound proteins". "Background" (quantity = B) refers to "background proteins" without "RNA-bound counterparts" (S = 0). For simplicity, "free proteins" refer to both unbound and background proteins; and unbound proteins included background proteins (i.e., $N = B$ when $S = 0$). However, true noise was considered distinguishable from true background (Supplementary Note 4d). "Observable proteins" refers to proteins identifiable by MS-based proteomic analysis via peptide mapping or proteins migrating at their expected (unbound) molecular weight during SDS-PAGE. "Observed proteins" or "Obs." (quantity = O) refers to proteins that were observable (e.g., observed proteins during SDS-PAGE of UV-crosslinked samples only included free proteins). In the absence of adjectives (e.g., RNA-bound), "proteins" refers to observable proteins. "Protein quantities" refers to their quantitative amounts. "Protein profiles" refers to "relative quantities" of proteins in the sample.

"SILAC LC-MS/MS analysis" refers to MS-based proteomic analysis of protein samples isolated from pooled input samples containing equivalent amounts of differentially SILAC-labeled UV-crosslinked and non-crosslinked samples. "LC-MS/MS analysis" refers to MS-based proteomic analysis of protein samples isolated from non-pooled input samples containing equivalent amounts of UV-crosslinked or non-crosslinked samples. For simplicity, "SILAC" and "non-SILAC" referred to SILAC LC-MS/MS and LC-MS/MS analysis respectively. "MS data analysis" refers to the analysis of MS datatsets generated by LC-MS/MS and SILAC LC-MS/MS experiments. "Specific" or "Non-specific UV-crosslinking" refers to photo-crosslinking of proteins to RNA or non-RNA substrates respectively. "UV-dependent enrichment of RNA" refers to the fold-enrichment of RNA in UV-crosslinked samples when compared to an equivalent % fraction of non-crosslinked sample. "UV-dependent enrichment of proteins" refers to the fold-enrichment of proteins in UV-crosslinked samples when compared to an equivalent % fraction of non-crosslinked sample and is represented by CL/nCL

ratios. “Significantly UV-enriched*” or “UV-enriched*” proteins displayed CL/nCL ratios significantly greater than 1 by statistical hypothesis testing during MS-based proteomic analysis (SILAC and non-SILAC). “RNA-bound protein enrichment” refers to the enrichment of RNA-bound proteins over their unbound counterparts. “*S*/*N* ratios” or “*S*/*N* of proteins” represents the ratio of RNA-bound to unbound counterparts. “Enrichment efficiency” refers to the magnitude of CL/nCL and/or *S*/*N* ratios.

SRA and SILAC LC-MS/MS analysis were considered *S*/*N*-based analyses because they distinguish RNA-bound proteins from unbound proteins and evaluated *S*/*N* (Supplementary Note 8a). Compared to SILAC LC-MS/MS, evaluating *S*/*N* by SRA was considered more accurate because non-specific UV-crosslinking does not contribute to the displayed *S*/*N* of proteins (i.e., *S*/*N* of observable protein quantities). Therefore, proteins which appeared “RNase-sensitive” ($|S| > 0$) by SRA analysis were considered “RNase-sensitive RBPs” or “bona fide RBPs” while proteins displaying “positive *S*/*N* ratios” by SILAC LC-MS/MS analysis were considered “UV-enriched” (i.e., CL/nCL ratios >1). The “*S*/*N* of RBPs” refers to the *S*/*N* of GO-annotated RBPs or RNase-sensitive RBPs, while the “*S*/*N* of non-RBPs” refers to the *S*/*N* of proteins without prior GO-annotations (GO:RBP). During MS data analysis, *S*/*N* ratios for non-RBPs represented the ratio of RNA-bound to unbound counterparts (SILAC and non-SILAC) despite the premise that non-specific UV-crosslinking to non-RNA substrates and/or UV-dependent enrichment of free protein can result in their apparent UV-enrichment (i.e., CL/nCL ratios >1 and *S*/*N* ratios >0). Observed quantities of proteins displaying *S*/*N* ratios greater than or less than 1 by *S*/*N* -based analyses were considered more representative of their RNA-bound or unbound quantities respectively. Observed quantities of proteins displaying *S*/*N* ratios >3 by *S*/*N*-based analyses were considered representative of their RNA-bound quantities (>75% RNA-bound). Conversely, observed quantities of proteins displaying *S*/*N* greater less than 0.33 were considered representative of their unbound quantities (<25% RNA-bound); Supplementary Note 6.

3. Description of quantitative metrics.

“Yield” represents the quantity of RNA or protein recovered by a given process expressed as micrograms per one percent of starting sample fraction (e.g., µg/% fraction). “Recovery” represents the quantity of RNA or protein isolated by a given process expressed as a percentage of the starting quantity (e.g., %RNA recovery). “Comparable recovery” refers to a non-significant difference in yield. “Near 100% recovery” refers to a non-significant difference in yield when compared to a suitable control. “Signal recovery” refers to RNA-bound protein or protein-bound RNA recovery. While signal referred to RNA-bound proteins, evaluating signal recovery by comparing protein-bound RNA yield was considered more accurate (Supplementary Note 4e, f). “Recovery of noise” refers to unbound protein recovery (Supplementary Note 4a). “Without signal loss” refers to an indiscernible decrease in signal recovery as compared to a suitable control. “RBP-specific signal loss” refers to discernible, varied decrease in recovery of RNA-bound RBPs. “High yield” and “low yield” methods were considered processes with high and low recovery respectively.

The following metrics were used when the isolation processes employed recovered near 100% of RNA and/or protein from samples, and when the indicated population contributed >90% of recovered RNA (UV-spectrophotometry) and/or protein (BCA) by mass (Supplementary Note 3). These RNA and protein populations can be described as total populations of starting samples, or herein, total cellular populations. RNA and protein recovered from AGP input suspensions by LEAP-RBP (with DNA depletion step) were considered representative of “total RNA” and “total RNA-bound protein” respectively. RNA recovered from the final AGPC interphase (with or without resuspension) by any process capable of near 100% RNA recovery (with or without DNA depletion step) was considered representative of “total protein-bound RNA”. However, only protein recovered from final AGPC interphase suspensions by LEAP-RBP (with or without DNA depletion step) was considered representative of total RNA-bound protein (i.e., “total clRNPs”). Protein recovered from AGP input suspensions by methanol (95% v/v) precipitation was considered representative of “total protein”, or herein, total cellular protein.

For clarity, total protein was distinguished from “total protein isolated” or “total protein in X” where X denotes the sample or fraction (e.g., total protein in LEAP-RBP fractions was considered representative of total RNA-bound protein). “Total protein abundance” represents protein quantity per µg of total protein but was distinguished from “protein abundance” representing protein quantity as a percentage of “total protein in the

sample" [1-3]. Nonetheless, protein abundances estimated as a percentage of total protein were considered equivalent to their total protein abundances. "Total RNA-bound protein abundance" represents RNA-bound protein quantity per μg of total RNA-bound protein but was distinguished from "RNA-bound protein abundance" which represents RNA-bound protein quantity (SPI_S) as a percentage of total protein in the sample ($\%TP_S$). RNA-bound protein abundances estimated as a percentage of total RNA-bound protein were considered equivalent to their total RNA-bound protein abundances (Supplementary Note 4f).

"RNP compositions" represents the ratio of protein to RNA in RNP fractions and was estimated by dividing protein yields with their corresponding RNA yields. "clRNP compositions" represents the ratio of RNA-bound protein to protein-bound RNA in clRNP fractions and was estimated by dividing protein yields with their corresponding RNA yields. "Protein UV-crosslinking efficiency" represents the percentage of total protein UV-crosslinked to RNA and was estimated by dividing total RNA-bound protein yield with the corresponding total protein yield, multiplied by 100. "RNA UV-crosslinking efficiency" represents the percentage of total RNA UV-crosslinked to protein and was estimated by dividing total protein-bound RNA yield with the corresponding total RNA yield, multiplied by 100 (Supplementary Note 3).

4. Evaluating UV-dependent enrichment and *S*/*N* by SDS-PAGE RNase-sensitivity Assay (SRA).
To evaluate *S*/*N* by SRA, RNase-treated samples were compared to equivalent amounts of untreated samples by SDS-PAGE with SYBR Safe (RNA&DNA), Coomassie Blue (protein), and Silver Stain (RNA, DNA, and protein) staining, or immunoblot (Supplementary Note 2). "RNase-dependent fold-change" or "RNase-sensitivity" refers to the fold-change in observed protein quantity (denoted by $\Delta\log_2(O)$) between RNase-treated and untreated samples as shown in equation (1). Proteins were considered "RNase-sensitive" if they displayed discernible RNase-sensitivity and "RNase-insensitive" if they did not. All RNase-sensitive proteins were considered RNase-sensitive RBPs or bona fide RBPs while "RNase-insensitive proteins" were considered non-RBPs or RBPs with low *S*/*N*. UV-enriched proteins displaying CL/nCL ratios >1 by SILAC LC-MS/MS and which remained undetectable by SRA and immunoblot were not considered bona fide RBPs regardless of GO-annotation status (e.g., GRP94, a GO-annotated RBP). However, because this could be due to their low RNA-bound abundance, it was not considered confirmation that a protein lacks RNA-binding activity. The RNase-sensitivity of an RBP was considered linearly related to their *S*/*N*: $(|S|+N)_{\mathrm{RNase}}/(N)_{\mathrm{untreated}} = S/N + 1$. Therefore, an increase in ΔO was considered indicative of "enhanced *S*/*N*". The "sensitivity of SRA" refers to the detectability of RNase-sensitive RBPs during SRA analysis (e.g., SRA with Coomassie Blue (protein) staining or immunoblot). Because the amount of RNA-bound protein analyzed by SRA was determined by RNA quantity, depleting free RNA and concentrating protein-bound RNA enhanced the sensitivity of SRA (i.e., $|S|$/μg RNA). The RNase-sensitivity of total protein in the sample analyzed by SRA and Coomassie Blue (protein) staining was considered directly related to $\%TP_S$; the RNase-sensitivity (*S*/*N*) of individual RBPs was not (Supplementary Note 4e, 8a).

To evaluate UV-dependent enrichment by SRA, RNase-treated and untreated samples isolated from UV-crosslinked and non-crosslinked cells were normalized to % fraction and analyzed by SDS-PAGE with SYBR Safe (RNA&DNA), Coomassie Blue (protein), and Silver Stain (RNA, DNA, and protein) staining, or immunoblot (e.g., Supplementary Fig. 5a). "UV-enrichment of RNA" or "UV-enrichment of protein" referred to the fold-enrichment of RNA or protein in UV-crosslinked samples as compared to non-crosslinked samples respectively.

5. Estimating RBP-specific UV-crosslinking efficiencies and *S*/*N* ratios by SDS-PAGE and immunoblot.
In this study, *S*/*N* ratios of proteins were estimated by SILAC LC-MS/MS analysis of LEAP-RBP fractions (Supplementary Note 5, 6). However, estimating *S*/*N* of RBPs by comparing serially diluted RNase-treated samples to a corresponding untreated sample by SDS-PAGE and immunoblot was considered a reasonable alternative (Supplementary Fig. 2a). "RBP-specific UV-crosslinking efficiencies" represents the percentage of total protein quantity that was UV-crosslinked to RNA and was estimated by comparing serial dilutions of non-crosslinked total protein and RNase-treated total RNA-bound protein by SDS-PAGE and immunoblot (Supplementary Fig. 2a, Supplementary Note 3c). Notably, the latter was only considered appropriate when the observed protein quantity (RNase) was representative of RNA-bound quantity (i.e., display *S*/*N* ratios >3).

6. Evaluating total protein and total RNA-bound protein abundance by SDS-PAGE.
To evaluate total protein abundance, non-crosslinked or RNase-treated UV-crosslinked input samples were normalized to μg of total protein and analyzed by SDS-PAGE with Coomassie Blue (protein) staining or immunoblot (e.g., input, RNase; Fig. 7i, j). To evaluate total RNA-bound protein abundance, RNase-treated RNP fractions containing total RNA-bound protein were normalized to μg of total RNA or total protein-bound RNA and analyzed by SDS-PAGE with Coomassie Blue (protein) staining or immunoblot (e.g., clRNP fractions, RNase; Fig. 7i, j). Notably, this was only considered appropriate when the observed protein quantity (RNase) was representative of RNA-bound quantity (i.e., display S/N ratios >3). Three different classes of RNA-binding proteins were observed in this study. This includes more constitutive RBPs whose total abundance and RNA-bound abundance were similar between experimental samples (e.g., pAbPC1 or RPL4; Fig. 7j); differentially abundant RBPs whose total abundance and RNA-bound abundance vary between experimental samples but whose relative abundance in each fraction appears directly related (e.g., NCL or RPN1); and dynamic RBPs exhibiting a difference in RNA-bound abundance without a discernible difference in total abundance or vice versa (e.g., TIA1 and LRRC59).

7. MS data analysis.
For MS-based proteomic analysis, protein quantities were estimated as the sum of their identified peptide intensities or sum peptide intensities and were represented by SPI values. The sum of all SPI values or "total SPI" was equal to the total MS signal and was considered representative of total protein in the sample as defined by the TPA method [1-3]. Replicate samples were denoted by "R#" where # is the replicate sample number. Sum peptide intensities of proteins observed in the UV-crosslinked SILAC channel (SILAC) or UV-crosslinked sample (non-SILAC) were represented by "SPI_{CL}" values, while the sum of all SPI_{CL} values was represented by "total SPI_{CL}". Sum peptide intensities of proteins observed in the non-crosslinked SILAC channel (SILAC) or non-crosslinked sample (non-SILAC) were represented by "SPI_{nCL}" values, while the sum of all SPI_{nCL} values was represented by "total SPI_{nCL}". $Log_2(CL/nCL)$ and $\log_2(S/N)$ ratios were generated with SPI_{CL} values and average SPI_{nCL} values according to equations (2) and (3). Proteins only detected in UV-crosslinked samples were given the following pseudo-values: $\log_2(S/N) = 10$, $\log_2(CL/nCL) = 10$. Proteins displaying negative average $\log_2(CL/nCL)$ ratios were given pseudo-$\log_2(S/N)$ ratios of −10. Average SPI values and S/N ratios were used to estimate RNA-bound and free protein quantities which were represented by "SPI_S" and "SPI_N" values respectively. Unless indicated otherwise, $SPI = SPI_{nCL} + SPI_{CL} = SPI_O = SPI_S + SPI_N$ for both SILAC and non-SILAC LC-MS/MS experiments. Additional information, examples, and equations for Excel were included in the provided Source Data for Fig. 5-d.

The standard deviation of $\log_2(SPI)$ was used to calculate the detectable fold-change in observed quantities or detectable $\Delta\log_2(O)$ representing the 95% confidence interval of $\log_2(SPI)$ as demonstrated in provided Source Data for Fig. 5e. The average $\log_2(S/N)$ ratio was used to calculate the detectable fold-change in RNA-bound quantity or detectable $\Delta\log_2(S)$ representing the estimated change in $\log_2(SPI_S)$ necessary to elicit a detectable change in $\log_2(SPI)$; $\log_2(2\text{^}(SD/\text{sqrt}(n) * t_{crit}) - 1/(S + 1)) - \log_2(S/(S + 1))$ where $SD = SD$ of $\log_2(SPI)$, n = sample size, $S = 2\text{^}\log_2(S/N)$, and t_{crit} = the corresponding t-critical value for two-tailed 95% confidence interval. Detectable $\Delta\log_2(S)$ was estimated by assuming N is constant. This assumption was proven false but was considered less impactful for proteins representative of RNA-bound quantities (Supplementary Note 6c, d). Additional information, examples, proofs, and full equations for Excel were included in the provided Source Data for Fig. 5e.

The total RNA-bound protein in the sample was estimated as the sum of all SPI_S values and represented by "total SPI_S". The total free protein in the sample was estimated as the sum of all SPI_N values and represented by "total SPI_N". The absolute quantity of total RNA-bound protein in the sample was represented by total $|S|$ and was considered dependent on UV-crosslinking conditions (total $|S|$ in starting samples) and signal recovery. Total SPI_S of RNP fractions containing total protein-bound RNA was considered representative of total RNA-bound protein. Protein abundances were estimated using the TPA or 'Total Protein Approach' by dividing average SPI values with the average total SPI and were represented as a percentage of total protein in the sample (i.e., "%TP" values) [1-3]. %TP values and average S/N ratios were used to estimate the abundance of RNA-bound ("$\%TP_S$") and free protein ("$\%TP_N$") quantities as a percentage of total SPI according to equations (9-11). Cumulatively, $\%TP_S$ and $\%TP_N$ represented the estimated abundance of total

SPI_S and total SPI_N in the sample. Protein abundances estimated as a percentage of other total populations in the sample (e.g., total SPI_{CL}) were represented by "$\%TP_{(CL)}$" values, where the parenthetical text indicates the identity of the total protein population. *S/N* ratios generated by only considering the estimated noise contributions of UV-crosslinked samples were represented by $S/N_{(CL)}$ ratios (Supplementary Note 6a).

"Relative abundance" represents the ratio of protein abundances (%TP/%TP) or $\Delta\log_{10}$(%TP) and was considered equivalent to their relative quantities (SPI/SPI) or $\Delta\log_{10}$(SPI) (Source Data Fig. 7h, Supplementary Note 4f). A decrease in free protein recovery without a decrease in total $|S|$ was expected to increase $\%TP_S$ of RBPs and non-RBPs without altering their relative RNA-bound abundances ($\%TP_S/\%TP_S$). This was evidenced by comparable $\%TP_{(S)}$ contributions from RBPs and non-RBPs in both INP and LEAP-RBP fractions despite a large difference in $\%TP_S$ (Supplementary Note 8a). "Non-specific $\%TP_{(S)}$ contributions" referred to the $\%TP_{(S)}$ contributions of non-RBPs (Supplementary Note 7e). A "favorable increase in $\%TP_S$" was considered an increase in $\%TP_S$ which did not appreciably increase non-specific $\%TP_{(S)}$ contributions. "Non-specific UV-enrichment" referred to UV-enrichment of free protein and was expected to increase non-specific $\%TP_{(S)}$ contributions. Similar non-specific $\%TP_{(S)}$ contributions were observed for other RNA-centric methods utilizing SILAC LC-MS/MS to accurately quantify free protein recovery: 2.6 for XRNAX and ~5.0 for TRAPP [4, 5]. As expected, non-SILAC comparison resulted in high non-specific $\%TP_S$ contributions: 24.4 for OOPs and 28.4 for Ptex fractions [6, 7]. Notably, non-specific $\%TP_{(S)}$ contributions for the referenced RIC study were only 1.5% [8]. This was attributed to high $\%TP_S$ of the RIC method and the observation that current GO-annotations of RBPs are largely based on their UV-enrichment* status in prior RIC-like (non-SILAC) experiments (Supplementary Note 8b). For these reasons, $\%TP_S$ was considered a key metric when evaluating method specificity for RNA-bound RBPs because it provided key information about *S/N*, free protein contributions ($\%TP_N$), and non-specific UV-enrichment (Supplementary Note 7, 8).

Observed abundances (%TP) for proteins displaying *S/N* ratios >3 were considered representative of their RNA-bound abundances ($\%TP_S$) (i.e., $\%TP \approx \%TP_S$), Because the total protein in LEAP-RBP fractions was considered representative of total RNA-bound protein (total SPI $\approx$ total SPI_S), %TP values of proteins displaying *S/N* ratios >3 were considered representative of their total RNA-bound abundances ($\%TP \approx \%TP_S \approx \%TP_{(S)}$) (Supplementary Note 4f). %TP values were $\log_{10}$ normalized and adjusted by subtracting the minimum $\log_{10}$(%TP) value of the MS dataset. This adjustment of $\log_{10}$(%TP) values was done for graphical and RCS ranking purposes. Method specificity for RNA-bound RBPs was evaluated graphically by comparing the abundances ($\log_{10}$(%TP)) of RBPs and non-RBPs as a function of their average $\log_2(S/N)$ ratios. A larger range of protein abundances (highest %TP–lowest %TP or "%TP range") resulted in a larger $\log_{10}$(%TP) range and was considered indicative of an improved (i.e., lower) limit of detection ("LOD"). Cumulative frequency curves for comparison of adj. $\log_{10}$(%TP) values only included proteins detected in all UV-crosslinked samples; adjusted $\log_{10}$(%TP) range (0–6) was divided into 50 bins and the median values for each bin were plotted as a function of their cumulative frequencies with increasing %TP and represented as a percentage of total protein IDs. Cumulative frequency curves for average $\log_2(S/N)$ ratios were generated in the same way but only included proteins displaying positive *S/N* ratios; *S* can't be negative, $0/N = B$.

8. RCS rank analysis.

"RBP confidence scores" (denoted by RCS) were generated for proteins detected in all UV-crosslinked samples and represent the product of adj. $\log_{10}$(%TP) values and average $\log_2(S/N)$ ratios. Because protein abundance was an important contributor, a lower RCS ranking may result from MS-based quantitation biases [9]. For example, XRN1 was found to be enriched in clRNP fractions by SRA and immunoblot while LRRC59 was de-enriched. However, XRN1 ranked lower than LRRC59 by protein abundance (%TP rank) by SILAC LC-MS/MS; 866 vs 290 respectively (Supplementary Fig. 2a). Low-abundance proteins only detected in UV-crosslinked samples (red box; Fig. 6b) were not included because their *S/N* ratios were considered less meaningful and more indicative of their lower abundance and quantitative accuracy (Supplementary Note 8a).

RCS rank analysis was performed by binning proteins according to their ordinal RCS rank (each bin = 100 proteins) and calculating the number of GO-annotated RBPs, average RCS, cumulative %TP contributions, $\log_2$(average *S/N* ratios), average number of unique peptides, and average detectable $\Delta\log_2(S)$ for each bin. The primary purpose of RCS or %TP ranking was to identify UV-enriched proteins more likely to be

orthogonally validated by SRA and immunoblot. Accuracy of RCS ranking was considered dependent on method specificity for RNA-bound RBPs or $\%TP_S$ (Supplementary Note 8a). Notably, RCS ranking assumed higher UV-crosslinking efficiency, indicated by higher RNA-bound protein abundance, was indicative of physiological relevance [10]. A full list of RBPs and non-RBPs identified by SILAC LC-MS/MS of LEAP-RBP fractions and ranked by RCS is included in the provided Source Data for Fig. 6g.

9. Additional protocol information.

9a. Repeated guanidinium thiocyanate-phenol-chloroform extraction (AGPC) protocol.
A gel loading pipette tip attached to a P1000 pipette tip was used to remove the aqueous and organic phases while leaving the interphase undisturbed. Resolubilizing the AGPC interphase in AGP by pipetting prior to adding chloroform and mixing was found to decrease the maximum $\%TP_S$ of the final AGPC interphase (Supplementary Note 4e). Therefore, both were added sequentially, and samples were vigorously vortexed for 10 sec without pipetting. Residual organic or aqueous phase during repeated AGPC extraction did not impact results. However, most of the organic phase was removed prior to final suspension. To that point, the ability to remove most of the organic phase without disturbing the interphase served as a qualitative indicator that maximum $\%TP_S$ has been reached (Supplementary Fig. 10c-e, Supplementary Note 1a). Samples were not re-centrifuged if the interphase was disturbed while removing the organic phase. In this scenario, a conservative removal of the organic phase was performed, fresh AGPC was added, and the samples were re-extracted.

9b. Isolation of RNP fractions by LEAP-RBP.
LEAP-RBP was performed on 200 µL aliquots of AGP input suspensions (>6 parts AGP) or final AGPC interphase suspensions containing up to 55 µg RNA&DNA. Typically, most of the organic phase following repeated AGPC extraction was removed to avoid having to determine the optimal amount of chloroform to add. If necessary, one 200 µL aliquot of the final AGPC interphase suspension per sample was used to determine the appropriate volume of chloroform for precipitation of the remaining aliquots: 12 µL of chloroform were added and the sample was mixed by pulse vortexing several times; AGPC mixtures were kept off lid. If the AGPC mixture assumed a cloudy white appearance and retracted to the bottom of the tube (after step A; Fig. 2c), this indicated the optimal concentration of chloroform has been reached (14–16 µL). If necessary, an additional 2 µL of chloroform were added. Alternatively, most of the organic phase during repeated AGPC extractions and/or prior to the final resuspension of the AGPC interphase was found to speed up free protein depletion and eliminated the need to determine the optimal volume of chloroform to be added (14 µL). In these scenarios, signal loss which occurred during the repeated AGPC extraction step was corrected for using the strategy outlined in the provided Source Data for Supplementary Fig. 8d-g. Once AGPC mixtures assumed a cloudy appearance they were mixed by continuous vortexing for another 10 sec; AGPC mixtures were kept off lid. Aliquots of the AGP input suspension were processed using 14 µl chloroform. Unlike final AGPC interphase suspensions, a cloudy appearance before adding chloroform was not problematic. Using an appropriate volume of chloroform and keeping RNA concentrations above 10 ng/µL of AGP were necessary to ensure optimal recovery (Supplementary Note 1). Four parts of a precipitation solution containing 3.75 M LiCl (10515, VWR) and 50% isopropanol were gently added/layered onto the AGPC mixtures, and the tubes were closed. Using a sample rack, samples were slowly inverted to 90 degrees and/or until the AGPC mixture was displaced from the bottom of the tube, and then the rack was returned to an upright position followed by incubation on bench for 1 minute. This process was repeated at least four more times, switching the direction of inversion, increasing the angle, and increasing the speed during reversion. Additional inversions were used if residual AGPC mixture remained at the bottom of the tube and the final reversion was performed forcefully. The protein and RNA composition of AGP suspensions were found to alter the optimal mixing speed and/or number of inversions. In all cases, performing the initial three inversions slowly before increasing speed resulted in optimal recovery while additional inversions did not diminish $\%TP_S$ (Supplementary Note 1). Samples were then homogenized by vigorous vortexing (5 sec), centrifuged at 14,000 x g for 5 min at 20 ˚C, and supernatants were removed. RNP pellets were rinsed twice with 1 mL RT 95% methanol by inverting the tube 2-3 times and removing the supernatant; a syringe equipped with a 19 ga 1-1/2" needle facilitates easy removal of the supernatant from multiple samples. Leaving the 1st, 2nd, or 3rd 95% methanol wash on the RNP pellets overnight at room temperature did not result in more free protein recovery; however, failing to remove the methanol washes rapidly after inverting sample tubes does.

Before removing the final methanol wash, RNP pellets which remained adhered to the bottom of the tube need to be dislodged. This was done by sliding a P1000 pipette tip down the side of the tube and against the top of the pellets until they started to move. Then, a small volume of the final methanol was pipetted to fully displace the RNP pellet off the bottom of the tube. Following removal of the final methanol wash, pellets were transferred to a new tube before resuspension. This was done by pouring ~1 mL of 95% methanol from the new tube into the tube containing the RNP pellet and then immediately pouring it back into the new tube. The methanol was removed and RNP pellets were air dried by leaving the tube open and incubating for 10 min at RT. RNP pellets were resuspended at the desired concentration with 1% LiDS TE by incubating for 30 min at room temperature with occasional pipetting (90% sample volume, 8 times) at the 2-, 16-, and 30-min mark. If bubbles formed during resuspension, samples were incubated at 55 ˚C for 20 sec, mixed by vigorous vortexing for 5 sec, and centrifuged at 3,000 x g for 10 sec at 20 ˚C. To mitigate the formation of bubbles, pipette tips were centered above the bottom of the tube while aspirating 90% of the sample volume and gently swirled against the bottom of the tube while ejecting. RNP suspension were then used immediately or stored at −80 ˚C for up to a year. Working concentrations of LEAP-RBP isolated RNPs ranged from 0.1–4.0 µg of protein-bound RNA/µL.

9c. LEAP-RBP DNA depletion step.
Turbo DNase is strongly inhibited by LiDS and so for the DNA depletion step, RNP pellets were resuspended in TE buffer. Samples were gently resuspended while keeping the samples at the bottom of the tube; not doing so diminished recovery (Supplementary Note 1c). Samples were not quantified during this step. Using a new pipette tip for each sample suspension, 5 µL of a master mix containing TE buffer, 10X Turbo DNase buffer, and Turbo DNase were added and mixed by swirling the pipette tip for 3 sec. Samples were incubated at 37 ˚C for 15 min without agitation and nine parts (180 µL) fresh acid guanidinium thiocyanate-phenol (2:1) buffer were added. Samples were precipitated according to the LEAP-RBP protocol using 14 µL of chloroform and resuspended in 1% LiDS TE at the desired concentration; samples were vortexed for 10 sec before and after adding chloroform while keeping AGPC mixtures off lid.

9d. SDS-PAGE, SYBR Safe, Coomassie Blue, Silver Stain staining.
LB WS was added to samples for a final detergent concentration of 2% (3.6 µL per 12.0 µL reaction containing 4 µL 1% LiDS TE). Samples were heated for 15 min at 65 ˚C in a thermocycler with heated lid (98 ˚C) and chilled on ice for at least 2 min prior to loading. Samples were not kept on ice for extended periods of time to avoid precipitation of SDS. Empty wells were loaded with LB WS to match the amount of detergent in samples (4.2 µL for prior example). Samples were separated on a 0.75 mm, 15-well, 4–12% gradient polyacrylamide gel (6, 8, 10, 12% (1:1:1:1) resolver, 4% stacker) at constant voltage (80 V) for 1.5 hours at RT (Supplementary Note 2c). For dual SYBR Safe (DNA&RNA) and Coomassie Blue (protein) staining, each polyacrylamide gel was incubated in 30 mL of 1X TBE containing 1.2 µL SYBR Safe (S33102, Invitrogen) on an orbital shaker (65 rpm) for 20 min at RT and rinsed three times with 50 mL DI water prior to imaging. Then, each gel was incubated in 30 mL Coomassie Blue stain (25% isopropanol (v/v), 10% glacial acetic acid, and 0.05% (m/v) Coomassie Brilliant Blue G-250 (1610406, Biorad)) on an orbital shaker (65 rpm) for 20 min at RT, rinsed three times with 50 mL DI water, and de-stained by incubating in 30 mL pre-warmed 10% acetic acid (v/v) on an orbital shaker (65 rpm) for 10 min at RT. The destaining step was repeated followed by overnight standing incubation in 30 mL fresh destain solution at RT. Then, each gel was rinsed three times with 50 mL DI water, incubated on bench in 50 mL DI water for 20 min twice, and rinsed an additional three times with 50 mL DI water prior to imaging. Silver Stain staining was performed on Coomassie Blue stained gels using a ProteoSilver Stain Plus Silver Stain Kit (PROTSIL2, Sigma). Imaging of SYBR Safe, Coomassie Blue, and Silver Stain-stained gels was performed using an Amersham Imager 600 (see corresponding Source Data).

9e. Immunoblot.
Following separation by SDS-PAGE (detailed above), samples were transferred to nitrocellulose membranes using Bjerrum and Schafer-Nielsen transfer buffer (48 mM Tris and 39 mM glycine) supplemented with 10% methanol (v/v) and 0.03% SDS. For each transfer, the gel was equilibrated in 100 mL of 1X transfer buffer on an orbital shaker (65 rpm) for 15 min at RT. Then, one 7 x 9 cm nitrocellulose membrane and six 9 x 11 cm thin filter paper sections were added individually to the gel container. Transfers were done at constant voltage (20 V) for 30 min at RT using a Trans-Blot SD semi-dry electrophoretic transfer cell (170-3940, Bio-

Rad). Alternatively, samples were wet transferred to nitrocellulose membranes using 25 mM Tris, 96 mM glycine, 0.05% SDS, and 20% methanol (v/v). The gel, membrane, and filter papers were equilibrated in 100 mL of 1X transfer buffer as described previously. Transfers were done at constant voltage (24 V) overnight at 4 ˚C using a Bio-Rad Mini-Protean II system. Blocking and immunoblotting was performed for each protein target according to the conditions listed in Supplementary Data 1. Signal detection was performed using WesternBright ECL HRP substrate (K-12045, Advansta) and an Amersham Imager 600 (see corresponding Source Data).

9f. Sample preparation for MS proteomic analysis.

For Turbo DNase digestion of each input sample containing 20µg of total protein, 42.5 µL TE buffer were added and the sample was incubated on bench for 5 min at RT without pipetting. Then, 5 µL of 10X Turbo DNase buffer were added and the sample was incubated on bench for 2 min at RT without pipetting. Using a new pipette tip for each sample, 90% of the sample volume was pipetted 8 times while swirling the pipette tip against the bottom of the tube. Samples were incubated for 2 min at RT and the pipetting step was repeated using the same pipette tip following by incubation for an additional 2 min at RT. Using a new pipette tip for each sample, 2.5 µL of Turbo DNase were added and the sample was mixed by swirling the pipette tip for 5 sec. After RNase and Turbo DNase digestion steps and during methanol precipitation/washing steps, precipitates were less adherent to the side of microcentrifuge tubes. Therefore, supernatants were removed using a P200 pipette tip attached to a P1000 pipette tip or by using a syringe equipped with a 19 ga 1-1/2" needle while leaving 50–100 µL residual supernatant. Samples were centrifuged a second time after removing most of the final 95% methanol wash and before removing the residual 95% methanol (~100-200 µL) using a P10 pipette tip attached to a P1000 pipette tip. Protein concentration of samples was kept above 25 ng/µL as estimated by BCA quantitation to ensure efficient protein recovery by 95% methanol v/v.

Supplementary Notes
Supplementary Notes provide additional observations and rationale for successful applications of the methods herein, characterization of *S*/*N*, and *S*/*N*-based analyses.

Supplementary Note 1
This note provides supporting information and technical considerations for LEAP-RBP and DNA depletion step.

1a. Repeated AGPC extraction concentrates clRNPs and makes them amenable to LEAP-RBP.
The combination of AGPC extraction and LEAP-RBP is a powerful tool for rapid and efficient purification of RNA-bound protein from biological samples. AGP, more commonly known by its commercial name "Trizol", is a universal method for simultaneous purification of RNA, protein, and DNA from biological samples [11, 12]. The finding that UV-crosslinked RNA-protein adducts efficiently partition to the AGPC interphase is a notable discovery [5, 13]. In practice, AGPC extraction allows concentration of covalently bound RNA-protein adducts from very dilute samples for subsequent LEAP-RBP; add 4 parts AGP, 1 parts chloroform, and perform AGPC extraction. When in doubt, purified clRNPs isolated by LEAP-RBP under optimal conditions were diluted with the untested sample buffer and processed accordingly. Recovery efficiency was tested by comparing the isolated clRNPs to an equivalent % of the purified clRNPs used as input.

Shearing lysates with a syringe needle was necessary to allow removal of the aqueous phase without disturbing the interphase. We've empirically determined that syringe needle shearing of lysates or harsh vortexing of AGPC mixtures does not diminish UV-crosslinked RNP integrity. For example, clRNPs isolated from AGPC interphase suspensions following repeated AGPC extraction were comparable to RNP fractions isolated from AGP input suspensions without repeated AGPC extraction by SRA analysis (Fig. 3a, b); RNA samples isolated from clRNP fractions with repeated AGPC extractions were found to be of high quality by bioanalyzer (RIN > 9) (Supplementary Fig. 4a). These data demonstrate that shearing and vortexing does not impact RNP integrity or results. However, AGPC mixing conditions were found to impact the rate at which free protein is depleted from the AGPC interphase (Supplementary Fig. 10a). Another factor found to impact the rate of free protein depletion is the ratio of phenol to chloroform. To ensure the proper ratio is maintained during repeat extractions, pipette tips must be pre-wetted. This was done by pipetting to and from the stock solution a few times before adding solvents to samples in an identical fashion.

During repeated AGPC extraction, residual aqueous and organic phase did not impact the %TP_S of final AGPC interphase samples evaluated by SRA with Coomassie Blue (protein) staining nor does letting the samples equilibrate to room temperature (RT) following centrifugation. If the interphase was disturbed, the samples were not re-centrifuged. Instead, fresh AGPC were added, and the samples were re-extracted. The organic phase contains phenol and chloroform, and the aqueous phase contains GT and aqueous buffers (PBS, etc). Therefore, the ratio of phenol and chloroform was maintained even when different percentages of the aqueous and organic phases were removed if AGP and chloroform were added at the proper ratio. For the repeated AGPC extraction experiment presented in this study (Fig. 1b, c), AGPC extractions were performed in 2 mL microcentrifuge tubes. Because the interphase of non-crosslinked cells was more dispersed, the volume of aqueous and organic phase removed was limited to prevent introduction of technical artifacts (red dashed line; Supplementary Fig. 10c). The interphase of UV-crosslinked cells appeared to have two layers; a lower layer which appears more dispersed, and an inner or upper layer with higher apparent density and integrity (Supplementary Fig. 10d). When removing the organic phase, the lower layer was found to break apart and fall into the organic phase. We suspect the upper layer contains clRNPs but avoided removing either by limiting the volume of organic phase removed during the first few extractions. Repeated AGPC extractions appears to deplete the lower layer until only the upper layer remains (Supplementary Fig. 10e). Removing most of the organic phase was necessary for resuspension of the interphase in fresh AGP and LEAP-RBP (Supplementary Fig. 10f). The lower layer impeded organic phase removal. Therefore, performing repeated AGPC extractions until the lower layer was depleted facilitated organic phase removal (Supplementary Fig. 10e). Critically, repeated AGPC extractions is not necessary to isolate most RNA-bound protein with sufficient *S*/*N* by LEAP-RBP; RPN1 was the only exception observed (Fig. 3a, b). Therefore, the main purpose

of repeated AGPC extraction is to remove free RNA and enhance sensitivity of downstream applications e.g., SRA (Fig. 1b, c, Supplementary Fig. 10f) and concentrate clRNPs while making them amenable to LEAP-RBP (>6 parts AGP; Methods).

1b. LEAP-RBP provides rapid and efficient isolation of clRNPs from AGP suspensions.
The behavior of samples during the LEAP-RBP step varies depending on the composition of the AGP suspension. Initially, LEAP-RBP was developed to work on final AGPC interphase suspensions. It was assumed that removal of chloroform and solubilization of the interphase in AGP was necessary for high %TP_S. However, when chloroform was intentionally added to test this assumption, it was found to increase yield without diminishing %TP_S (Supplementary Fig. 11a). Under suboptimal conditions, proteins bound to small RNAs displayed decreased recovery when compared to an equivalent percentage of methanol (95% v/v) precipitated AGPC interphase samples. The dual-stained 65 kD clRNP (gold asterisk; Supplementary Fig. 11a) was determined to be a reliable indicator of small RNA-bound protein recovery as quantified by RT-qPCR. Earlier versions of LEAP-RBP without near 100% recovery showed diminished recovery of the 65 kD clRNP when compared INP fractions containing an equivalent amount of protein-bound RNA (gold box; Supplementary Fig. 11b); differences in the relative intensities of RNase-sensitive bands illustrates RBP-specific signal loss (gold asterisks; Supplementary Fig. 11b). The optimized LEAP-RBP method shows comparable recovery of the 65 kD clRNP from final AGPC interphase suspensions of UV-crosslinked cells as methanol (95% v/v) or INP precipitation methods (Fig. 2a). Comparison of LEAP-RBP fractions isolated from AGP input suspensions containing UV-crosslinked or non-crosslinked cells by SRA and SYBR Safe (RNA&DNA) staining demonstrates that the 65 kD clRNP is formed by UV-crosslinking (SYBR Safe stained gel; Fig. 3a, Supplementary Fig. 6b). Furthermore, separating proteinase K treated clRNP fractions by SDS-PAGE results in a downward shift of the SYBR Safe (RNA&DNA) stained RNA interactor to ~28 kD and is no longer visible with Coomassie Blue (protein) staining (Supplementary Fig. 3d). Based on these data, we estimate the protein and RNA interactors are roughly 36 kD and 100 bp (33 kD) respectively [7]. Serendipitously, the optimal volume of chloroform results in a noticeable qualitative change (Supplementary Fig. 11c); the optimal final concentration of chloroform was determined to be between 6–8% (Supplementary Methods). Solubilization of the AGPC interphase and re-introduction of chloroform ensures reproducible results.

All LEAP-RBP steps for this study were performed in 1.5 mL microcentrifuge tubes with a rounded bottom (490003, VWR); microcentrifuge tubes with pointed ends were found to impede mixing during inversions. AGPC interphases were routinely resuspended in ~1.3 mL fresh AGP and split across six 1.5 mL microcentrifuge tubes (200 µL each). Samples were centrifuged briefly to concentrate samples at the bottom of the tube (Supplementary Fig. 10f). The minimum amount of RNA for efficient recovery was found to be 1 µg but routinely kept above 2 µg (Supplementary Fig. 11d). We found it useful to estimate the expected yield under optimal conditions by diluting 20–50 µL of the original AGP suspension to 200 µL with fresh AGP and performing LEAP-RBP with the optimal volume of chloroform (14 µL). If the yield was above 2 µg, it was used to estimate the yield of the undiluted aliquots. For the undiluted aliquots, 12 µL of chloroform were added initially and samples were pulse vortexed several times; AGPC mixtures were kept off lid. If sample appearance did not change (Supplementary Fig. 11c), an additional 2 µL were added and the samples were pulse vortexed again. In addition to the cloudy-white appearance, AGPC mixtures were found to retract to the bottom of the tube after brief vortexing if the optimal amount of chloroform has been added (Fig. 2c). Turbo DNase treated samples mixed with AGP were an exception and did not stick to the side of the tubes. When performing LEAP-RBP on AGP suspensions with low protein-content, the emulsion of AGPC mixtures may separate prior to adding the precipitation solution. Extending the duration of most steps to 3 min did not impact the results (asterisk; Supplementary Fig. 11e).

During the LEAP step, samples are inverted to increase the surface area between the two solvents. Various mixing methods were tested, and the inversion method was found to be the most consistent. Gently mixing the two solvents during the first few inversions was critical to optimal yields (Supplementary Fig. 11f). The appearance and behavior of samples were found to be dependent on sample composition; samples with lower protein levels mixed more readily (e.g., Turbo DNase treated RNP samples isolated by LEAP-RBP); samples with more protein required more forceful inversions after the first 3 inversions to fully mix (e.g., input AGP-

suspensions containing 400 μg protein and upwards of 55 μg nucleic acids). Excess chloroform results in a visible phase-separation (Supplementary Fig. 11g); samples with a sharp phase-boundary containing visible precipitates were not used.

1c. LEAP-RBP allows rapid and efficient isolation of total RNA from AGP input suspensions or following DNA-digestion.

As noted, AGP input suspensions appear cloudy before chloroform addition; the optimal volume of chloroform (14 μL) was added for the LEAP-RBP step regardless of sample appearance. Samples appeared clarified following inversion(s) and mixing by vortex. The use of methanol vs other alcohols (e.g., EtOH) or aqueous-based washes (LiCl) was chosen because methanol is more efficient at removing lipids, saccharides, guanidinium thiocyanate, and phenol than ethanol or aqueous based solvent. After a single methanol rinse, samples were found free of contaminants such as GT and phenol, as determined by UV-spectrophotometry (Supplementary Fig. 11h).

The integrity of microcentrifuge tubes was apparently diminished with each LEAP step. Therefore, pellets were transferred to a new tube before resuspension. This was done by pouring ~1 mL of 95% methanol from the new tube into the tube containing the RNP pellet and then immediately pouring it back into the new tube. For experiments where precision was key, multiple aliquots were processed in parallel and pooled prior to resuspension to reduce technical variability. For example, samples for SILAC LC-MS/MS contained 150 μg of protein-bound RNA spread across six 1.5 mL microcentrifuge tubes. Following the initial LEAP-RBP step, pellets were combined into three tubes for DNA depletion. Following DNA depletion and the second LEAP, pellets were again pooled for resuspension and LC-MS/MS sample prep.

During the DNA depletion step, samples were maintained at the bottom of the tube. TE suspended clRNPs adhere to the sides of microcentrifuge tubes; not keeping samples at the bottom of the tubes led to sample loss. Samples were incubated in 15 μL TE buffer for 2 min and then pipetted gently 8 times (5 μL). Samples were incubated at RT for 2 min and the process was repeated if necessary. Depending on RNA concentration and UV-crosslinking conditions (i.e., μg RNA-bound protein/μg RNA), samples may not resolubilize completely even after extended incubation and pipetting. This does not affect DNA digestion efficiency or recovery if samples are incubated in TE for a minimum of 5 min and triturated as noted previously. Samples containing clRNPs take on a cloudy appearance upon addition of Turbo DNase buffer and adhere to pipette tips (Supplementary Fig. 11i). Therefore, the master mix containing Turbo DNase buffer and Turbo DNase were added directly to the bottom of the sample suspension and instead of pipetting, samples were mixed by swirling the pipette tip. The DNA depletion step was designed to overcome UV-dependent alterations in sample physical properties and allow efficient DNA digestion and subsequent recovery by LEAP-RBP. Following Turbo DNase digestion and re-introduction of AGP, it was necessary to vortex samples for 10 sec at medium setting before and after adding chloroform for efficient recovery during the second LEAP. During the second LEAP, UV-crosslinked samples were found to behave differently than non-crosslinked samples containing equivalent amounts of RNA (Supplementary Fig. 11j). A ~10% decrease in quantifiable RNA by UV-spectrophotometry after performing the DNA depletion step on clRNP fractions was expected; no apparent differences in signal recovery or RNase-sensitive protein profiles were observed (Fig. 3a, b, Supplementary Fig. 5b). Samples and buffers were kept at RT during all steps unless indicated otherwise.

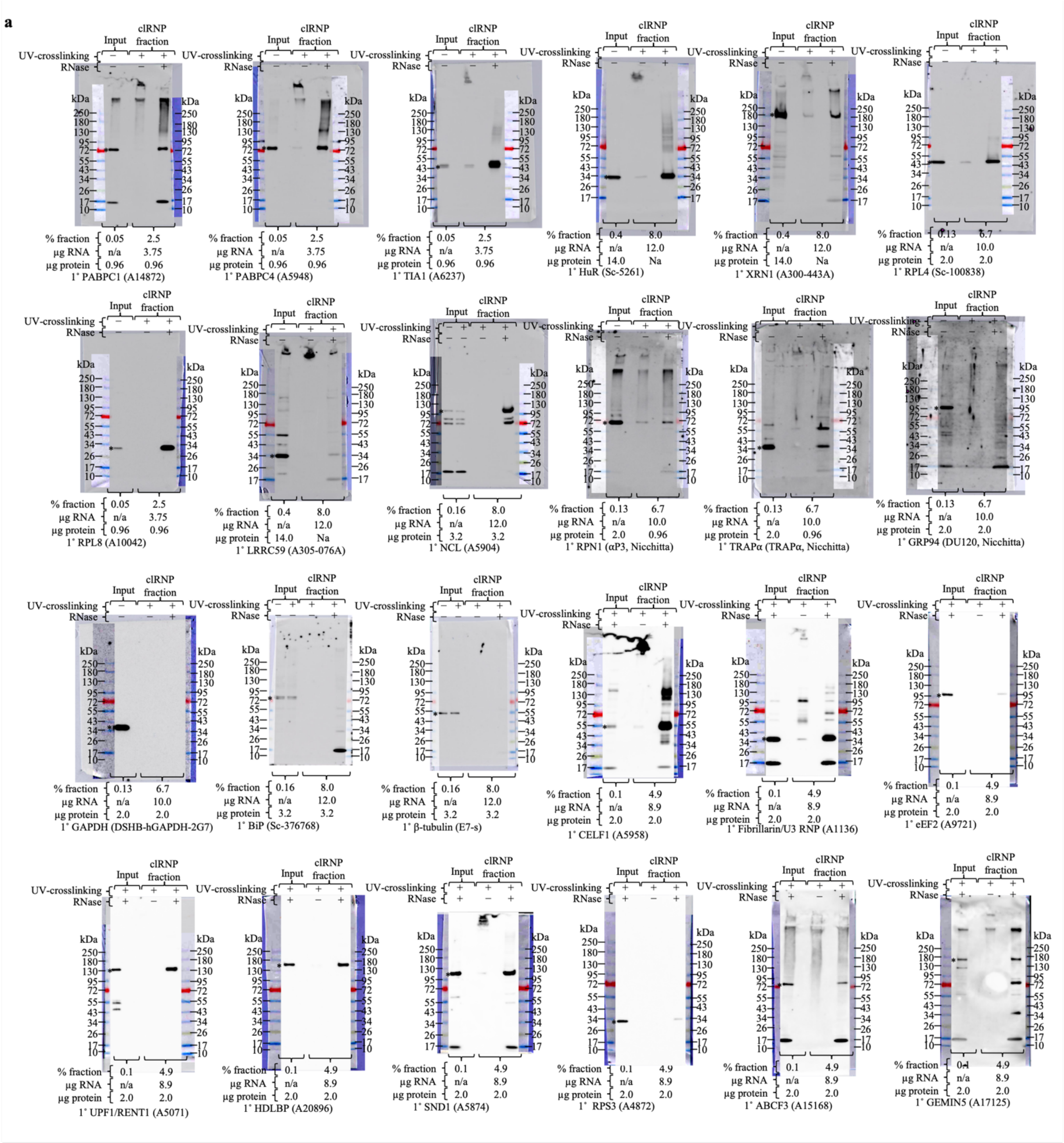

Supplementary Figure 1: Full blots for WB targets used in main figures. See Supplementary Data 1 and Methods for blocking and blotting conditions used for each target. **a** Input samples (total protein) were isolated from AGP input suspensions containing UV-crosslinked (0.4 J/cm^2, 254 nm) or non-crosslinked HeLa cells. LEAP-RBP fractions were isolated from final AGPC interphase suspensions of UV-crosslinked (0.4 J/cm^2, 254 nm) HeLa cells; sample compositions and/or normalization values indicated in figure panels.

a

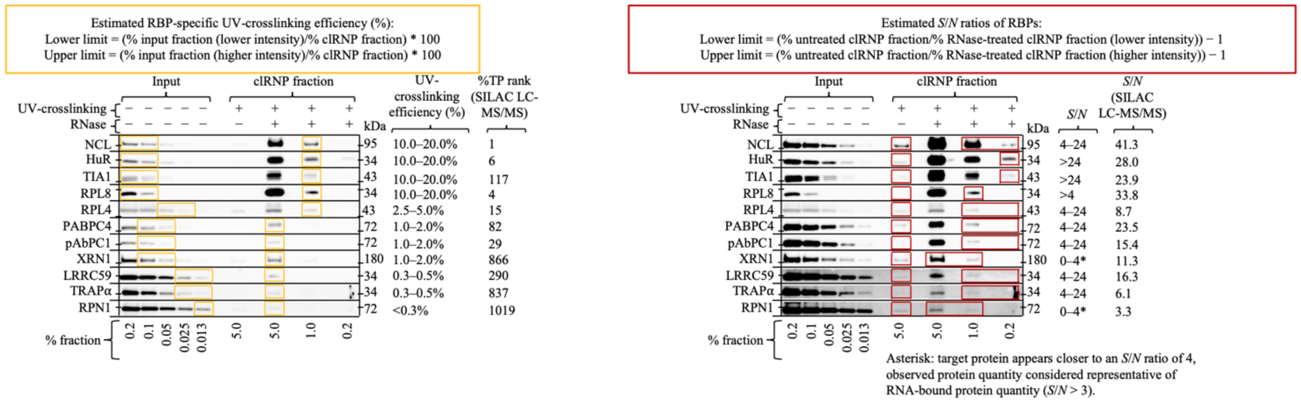

Supplementary Figure 2: LEAP-RBP allows determinations of RBP-specific UV-crosslinking efficiencies and *S*/*N* ratios. a Estimation of RBP-specific UV-crosslinking efficiencies and *S*/*N* ratios by comparing serial dilutions of input and RNase-treated clRNP fractions, or serially diluted clRNP fractions to a corresponding untreated sample with SDS-PAGE and immunoblot respectively; *n* = 3 biologically independent samples, pooled (equivalent % fraction) for SRA analysis. %TP rank or *S*/*N* of proteins (SILAC LC-MS/MS) based on results of LEAP-RBP SILAC LC-MS/MS experiment (Supplementary Data 3). Experiments (**a**) were performed twice with similar results. Source Data are provided as Source Data file.

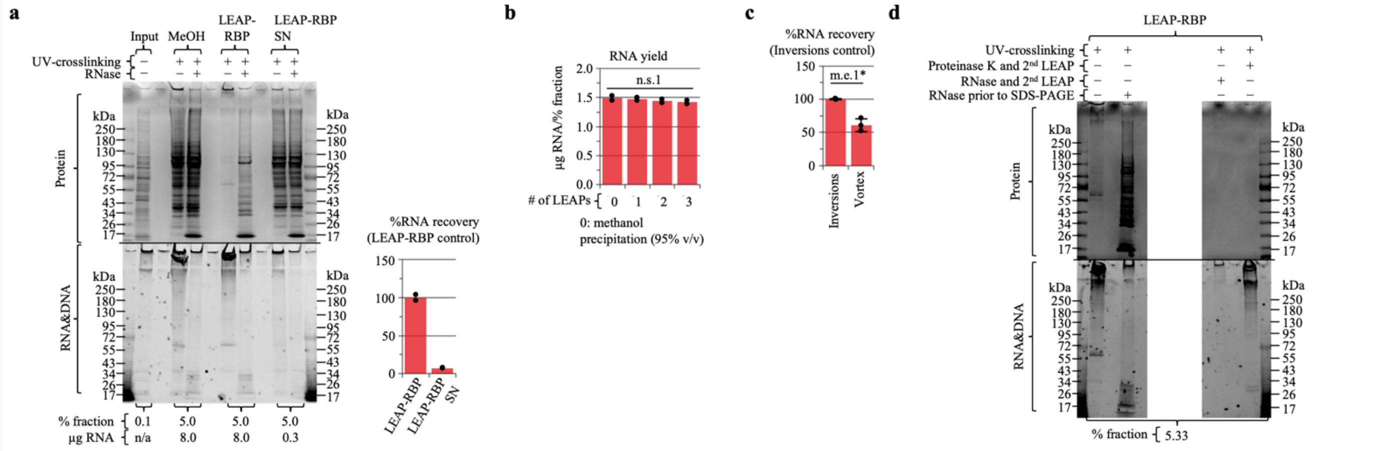

Supplementary Figure 3: Validation of LEAP-RBP clRNP recovery efficiency and RNA-centricity. AGPC: acidic guanidinium thiocyanate-phenol-chloroform. **a** Comparison of methanol, LEAP-RBP, and LEAP-RBP supernatant ("SN") fractions isolated from final AGPC interphase suspensions of UV-crosslinked cells (0.4 J/cm^2, 254 nm) by SRA and Coomassie Blue (protein), SYBR Safe (RNA&DNA) staining. RNA recovery shown in adjoining bar chart represents the mean of 2 independent samples. Most of the observed absorbance during UV-spectrophotometric analysis of LEAP-RBP supernatant fractions is from protein, not RNA (see provided Source Data file). **b** Performing repeated LEAP steps does not decrease RNA recovery (i.e., RNA yield). RNA recovery shown in bar chart represents the mean ± 1 *SD* of 2 independent samples isolated from separate aliquots of the same final AGPC interphase suspension (biological variability was not of interest). Effect of isolation method (methanol, 1–3 LEAPs) on RNA recovery analyzed using one-way ANOVA: non-significant main effect of isolation method (n.s.1), $F(3, 4) = 0.73$, $p = .585$. **c** Gentle inversion(s) and emulsion formation enhances RNA recovery. RNA recovery shown in bar chart represents the mean ± 1 *SD* of 3 independent samples isolated from separate aliquots of the same final AGPC interphase suspension (biological variability is not of interest). Effect of mixing method (inversions, vortex) on RNA recovery analyzed using one-way ANOVA: significant main effect of mixing method (m.e.1), $F(1, 4) = 30.88$, $p = .005$. Note that this is equivalent to an unpaired, two-tailed, homoscedastic *t* test (see provided Source Data file). **d** Validation of LEAP-RBP RNA-centricity. Performing LEAP-RBP on RNase-treated LEAP-RBP fractions results in a loss of detectable protein by SDS-PAGE and Coomassie Blue (protein) staining. Performing LEAP-RBP on proteinase K treated LEAP-RBP fractions results in recovery of visible RNA by SDS-PAGE and SYBR Safe (RNA&DNA) staining but no protein was detected by Coomassie Blue staining. Experiments were performed once (**a**, **d**), or three times with similar results (**b**, **c**). Source data are provided as a Source Data file.

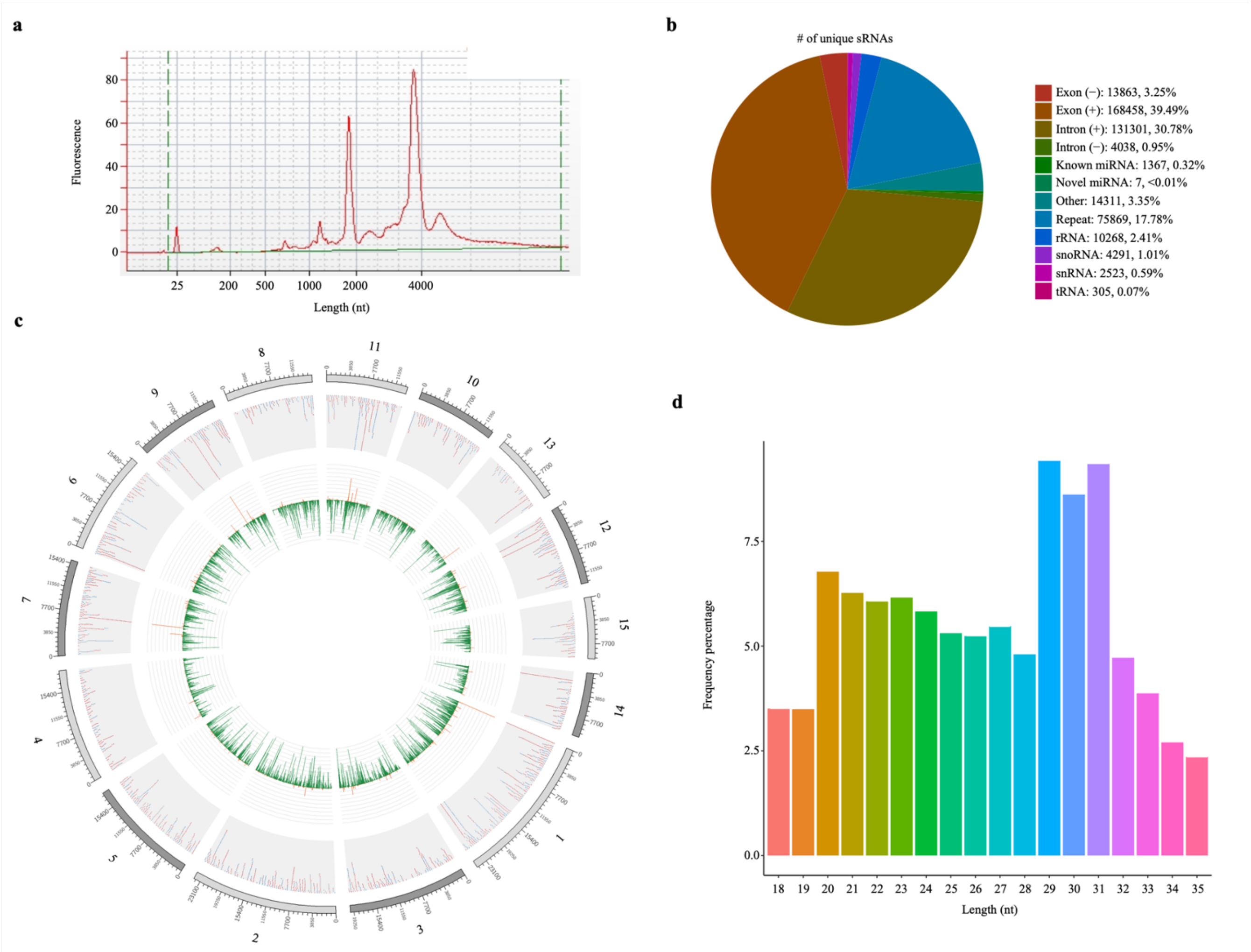

Supplementary Figure 4: Small RNA-species are well represented in LEAP-RBP fractions. NGPC: neutral guanidinium thiocyanate-phenol-chloroform. **a** Representative bioanalyzer trace of RNA samples isolated by NGPC extraction of proteinase K treated LEAP-RBP fractions demonstrating high rRNA integrity (= RIN). **b** Pie chart depicting the number and percentage of unique sRNA reads mapped to sense (+) or antisense (−) intronic or exonic regions, known miRNAs, novel miRNAs, other (mapped to genome but lacking annotation), repeat regions, rRNA, tRNA, snRNA, and snoRNA. Small RNA species were annotated according to the following priority: known miRNA > rRNA > tRNA > snRNA > snoRNA > repeat > gene > novel miRNA. **c** Circus plot depicting sRNA read distribution per chromosome (outer circle). Grey background in the middle area shows the distribution of 10,000 reads on each chromosome; red represents the number of sRNAs on the sense strand while blue represents the number of sRNAs on the antisense strand. All reads are shown in the center area of the circle; yellow represents the number of sRNAs on the sense strand while green represents the number of sRNAs on the antisense strand. **d** length distribution analysis of clean reads depicting composition of small RNA sample: miRNA are expected to be 21–22 nucleotides (nt) in length, siRNA 24 nt, and piRNA 28–30 nt. The sRNA-seq experiment was performed twice using clRNP fractions isolated from biologically independent samples with similar RIN values.

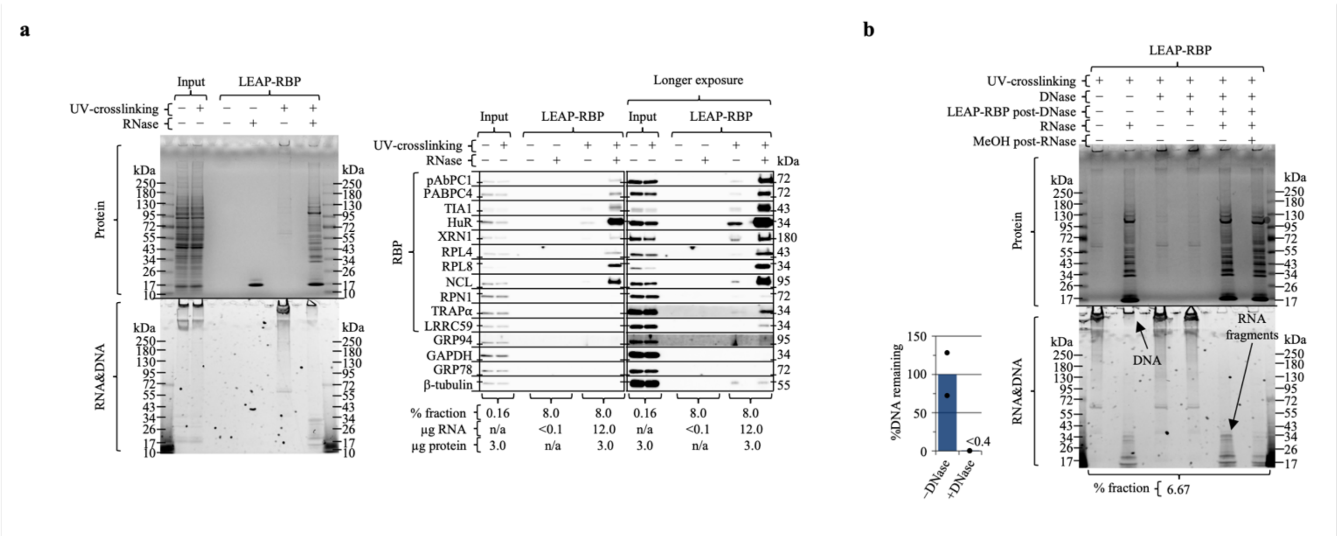

Supplementary Figure 5: LEAP-RBP fraction display high UV-dependent enrichment. AGPC: acidic guanidinium thiocyanate-phenol-chloroform. **a** UV-dependent enrichment of RNA and DNA as well as *S*/*N* of protein isolated by LEAP-RBP from repeat AGPC extraction interphase suspensions were evaluated by SRA with SYBR Safe (RNA&DNA) and Coomassie Blue (protein) staining or immunoblot; $n = 3$ biologically independent samples, pooled (equivalent % fraction) for SRA analysis; sample compositions and normalization values indicated in figure panels. **b** Validation of Turbo DNase digest efficiency and depletion of RNase-digested RNA fragments by methanol precipitation for downstream LC-MS/MS analysis: Turbo DNase digestion of LEAP-RBP fractions depletes visible RNase-insensitive DNA retained in the stacker during SDS-PAGE. Methanol precipitation (95% v/v) of RNase-treated LEAP-RBP fractions depletes RNA fragments visible by SDS-PAGE and SYBR Safe staining. DNA digestion efficiency determined by qPCR of Turbo DNase treated and untreated LEAP-RBP fractions; data represents the mean of two independently prepared samples as a percentage of DNA remaining compared to the average of untreated samples. Experiments (**a**, **b**) were performed three times with similar results. Source data are provided as a Source Data file.

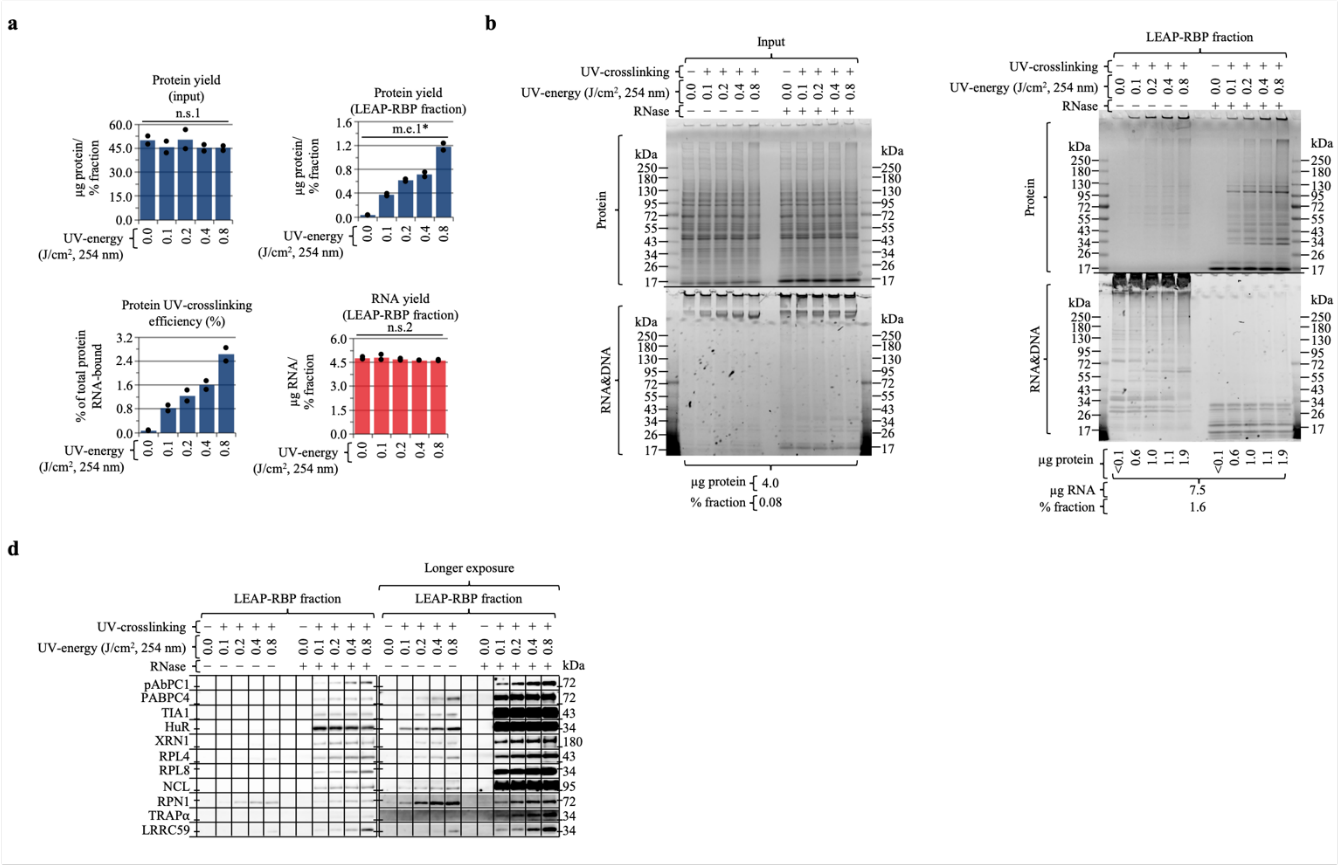

Supplementary Figure 6: UV-dose or signal-dependent recovery recovery of noise. AGP: acidic guanidinium thiocyanate-phenol. **a-c** AGP input suspensions containing equivalent amounts of HeLa cells irradiated with increasing UV-energy (254 nm) were split before isolation of input and LEAP-RBP fractions and thus treated as independent samples ($n = 2$ independent samples for each UV-dose). Biological variability could only be measured across sample groups ($n = 5$ biologically independent samples) thus post-hoc tests between sample groups ($n = 2$) were omitted (**a**). Protein (BCA) and RNA (UV-spectrophotometry) yields shown in bar charts (**a**) represent the mean of two independent samples. Protein UV-crosslinking efficiencies were calculated using protein yields of the corresponding input samples and LEAP-RBP fractions of each independent sample, the bar chart (**a**) shows the mean. **a** Effect of UV-dose and fraction (input, LEAP-RBP) on protein yield analyzed two-way ANOVA: significant interaction, $F(4, 10) = 272.99$, $p < .001$. Subdivided by largest main effect (m.e.), fraction (separate graphs), $F(1, 10) = 17086.32$, $p < .001$. Effect of UV-dose on protein yield of input and LEAP-RBP subgroups analyzed using independent one-way ANOVAs: input, non-significant m.e. of UV-dose (n.s.1), $F(4, 5) = 0.62$, $p = .668$; LEAP-RBP, significant m.e. of UV-dose (m.e.1*), $F(4, 5) = 1015.12$, $p < .001$. To test whether the main effect of UV-dose on protein yield of LEAP-RBP was independent of RNA yield, the effect of UV-dose and macromolecule (protein, RNA) on yield (µg macromolecule/% fraction) of LEAP-RBP was analyzed using two-way ANOVA: significant interaction, $F(4, 10) = 848.30$, $p < .001$. Subdivided by largest m.e., macromolecule (separate graphs), $F(1, 10) = 11902.04$, $p < .001$. Effect of UV-dose on yield of LEAP-RBP for protein subgroup tested above. Effect of UV-dose on yield of LEAP-RBP for RNA subgroup analyzed using one-way ANOVA: non-significant m.e. of UV-dose (n.s.2), $F(4, 5) = 0.89$, $p = .534$. Independent samples from each sample groups were pooled (equivalent % fraction) and analyzed by SRA and SYBR Safe (RNA&DNA), Coomassie Blue (protein) staining (**b**), or immunoblot (**c**); sample compositions indicated in panel **b**. Experiments (**a-c**) were performed three times with similar results. Source data are provided as a Source Data file.

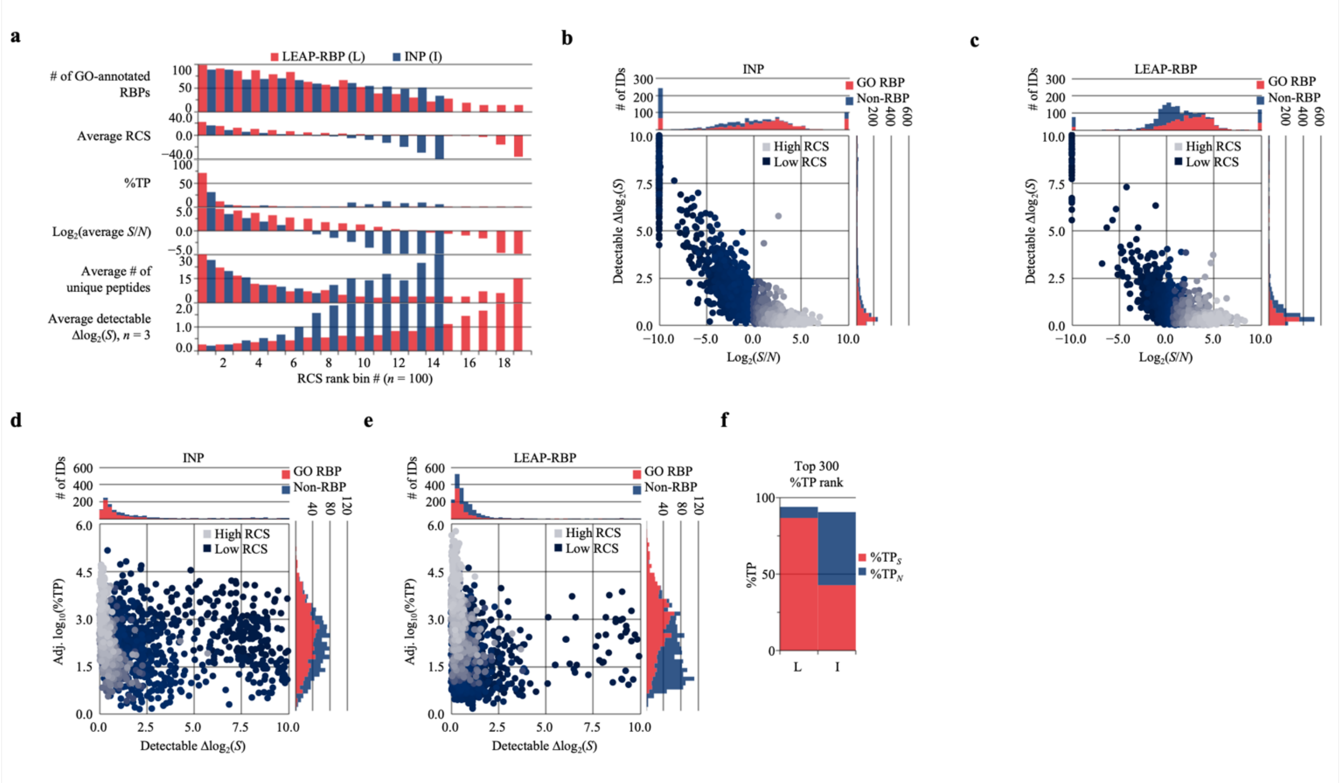

Supplementary Figure 7: Enhanced *S*/*N* and improved specificity of LEAP-RBP method (%TP$_S$) increases detection sensitivity of Δlog$_2$(*S*) as compared to the INP method. a-f Summary of RCS rank analysis (**a**). For methods with high %TP$_S$, RCS rank predicts log$_2$(*S/N*) (**b**,**c**), %TP (**d**, **e**), coverage (# of unique peptides), or detectable Δlog$_2$(*S*). Color overlay based on ordinal RCS rank. **f** Estimated %TP$_S$ and %TP$_N$ contributions of the 300 most abundant proteins in LEAP-RBP or INP fractions. Source data are provided as a Source Data file.

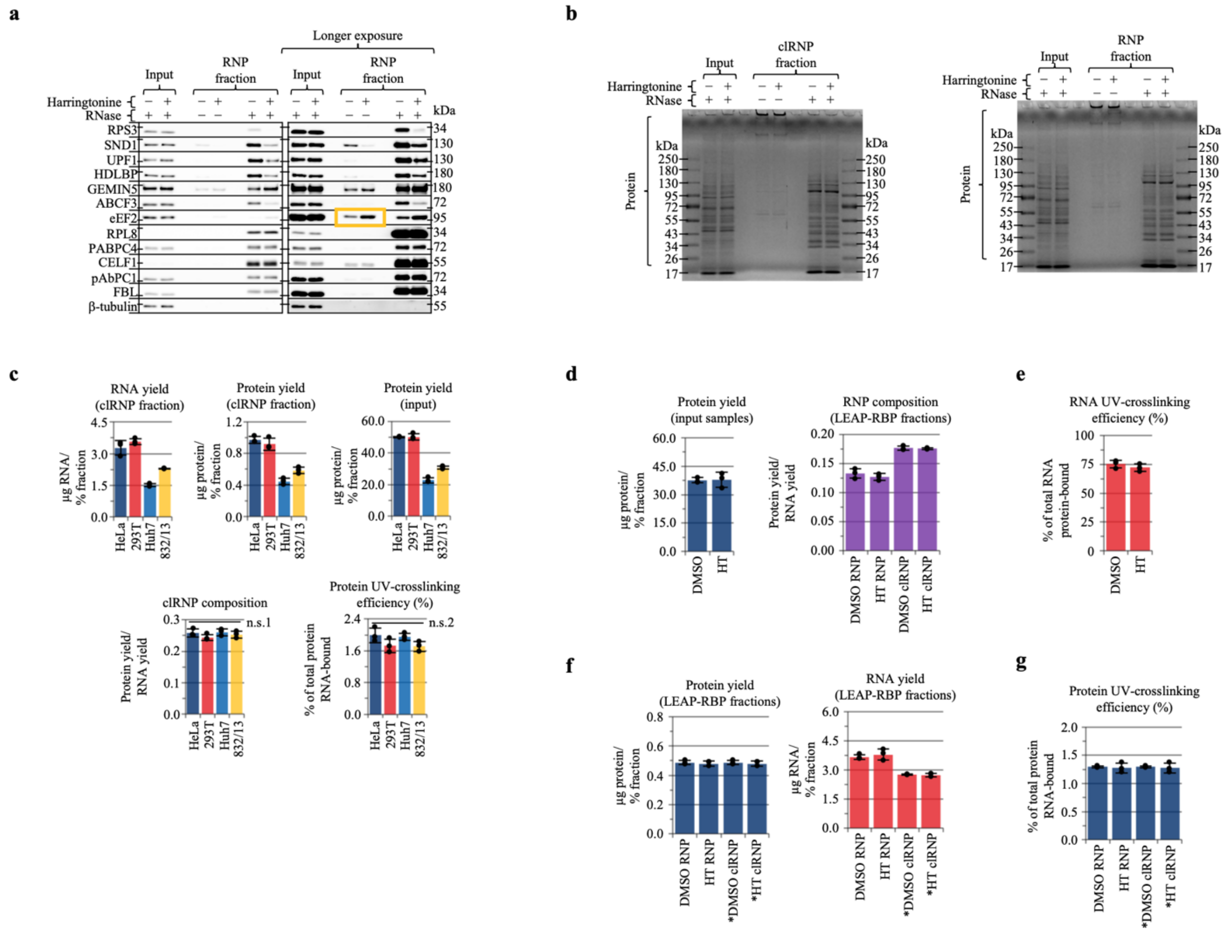

Supplementary Figure 8: LEAP-RBP allows robust and accurate comparisons of RNA-bound proteomes. AGP: acidic guanidinium thiocyanate-phenol; HT: harringtonine. RNA (UV-spectrophotometry) and protein (BCA) quantitation data represents the mean ± 1 *SD* of 3 biologically independent samples. **a** Comparison of input and RNP fractions isolated from corresponding AGP input suspensions of samples shown in Fig. 7g by SRA and immunoblot. **b** Comparisons of samples shown in Fig. 7g and panel **a** by SRA and Coomassie Blue (protein) staining, parallel gels. **c** Effect of cell line on clRNP composition (µg protein/µg RNA) or protein UV-crosslinking efficiency analyzed using independent one-way ANOVAs: clRNP composition, non-significant main effect of cell line (n.s.1), $F(3, 8) = 1.38$, $p = .318$; protein UV-crosslinking efficiency, non-significant main effect of cell line (n.s.2), $F(3, 8) = 2.97$, $p = .097$. **d** Bar charts showing protein yields of input samples or compositions of RNP and clRNP fractions shown in panel **b**. **e** Estimated RNA UV-crosslinking efficiencies of samples shown in panel **b** calculated using compositions of corresponding RNP and clRNP fractions shown in panel **d**. Adjustment of RNA and protein yields for clRNP fractions was performed by comparing the estimated RNA UV-crosslinking efficiency calculated using RNA yields vs composition (µg protein/µg RNA) of RNP and clRNP fractions. Additional information included in the provided Source Data file. **f** Bar charts showing adjusted (*) or non-adjusted protein or RNA yields of cRNP and RNP fractions shown in panel **b**. **g** Estimated protein UV-crosslinking efficiencies of samples shown in panel **b** calculated using protein yield of input samples shown in panel **d** and adjusted (*) or non-adjusted protein yields of clRNP and RNP fractions shown in panel **f**; note how adjustment of protein yields for clRNP fractions results in equivalent comparison of estimated protein UV-crosslinking efficiencies between sample groups (DMSO vs HT) with either fraction (RNP or clRNP). Experiments were performed once (**a**, **b**, **d-g**), or three times with similar results (**c**). Adjustment of clRNP fraction yields using the strategy included in the provided Source Data for panels **d-g** was performed during three independent experiments with similar results (**b**). Source data are provided as a Source Data file.

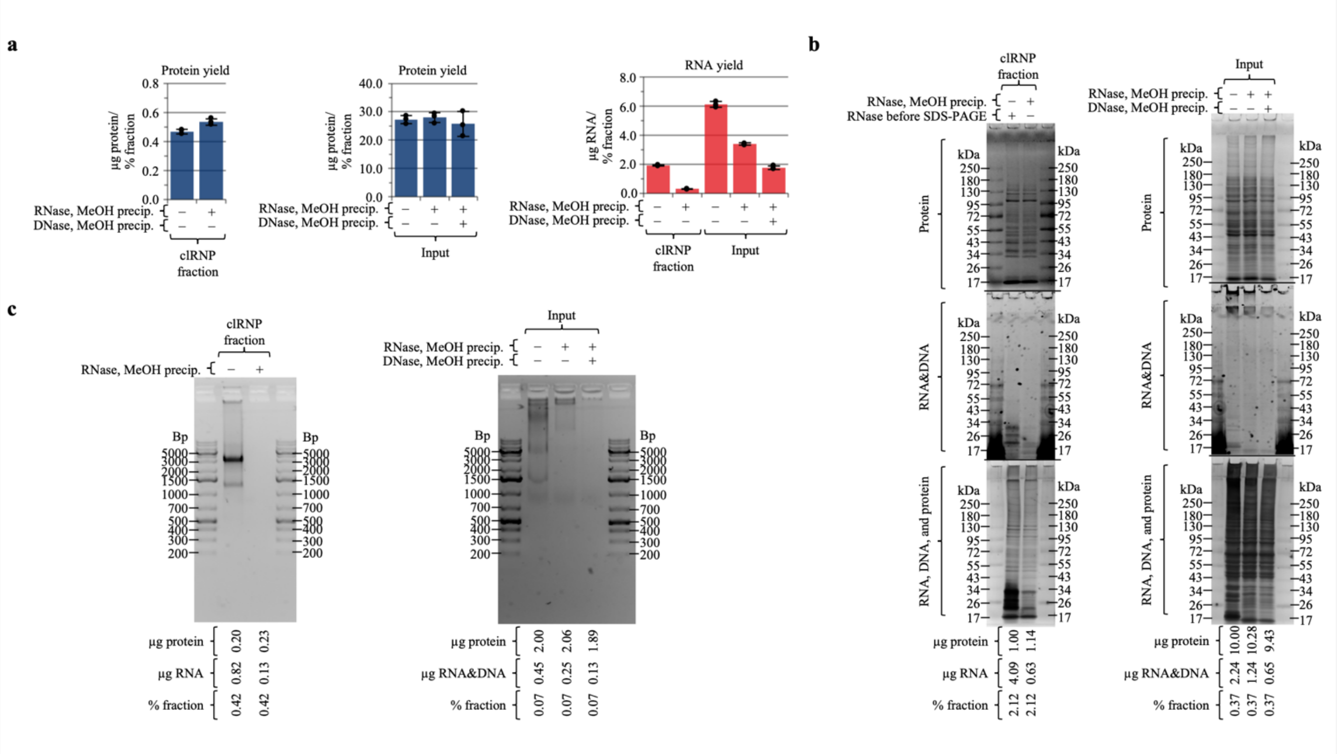

Supplementary Figure 9: Validation of RNA and DNA depletion steps for proteomic sample preparation. AGPC: acidic guanidinium thiocyanate-phenol-chloroform; AGP: acidic guanidinium thiocyanate-phenol. **a** Bar charts showing protein (BCA) or RNA (UV-spectrophotometry) yields of clRNP fractions and/or input samples with or without DNA and/or RNA depletion steps; data represents the mean ± 1 *SD* of 3 independent samples isolated from separate aliquots of the same AGP input suspension (input) and corresponding final AGPC interphase suspension (clRNP). Biological variability was not of interest for this experiment. Replicate samples were pooled (equivalent % fraction) and evaluated by SRA with SYBR Safe (RNA&DNA), Coomassie Blue (protein), and Silver Stain (RNA, DNA, and protein) staining (**b**) or TBE gel analysis (**c**); sample normalization values indicated in figure panels. Experiments (**a**-**c**) were performed three times with similar results. Source data are provided as a Source Data file.

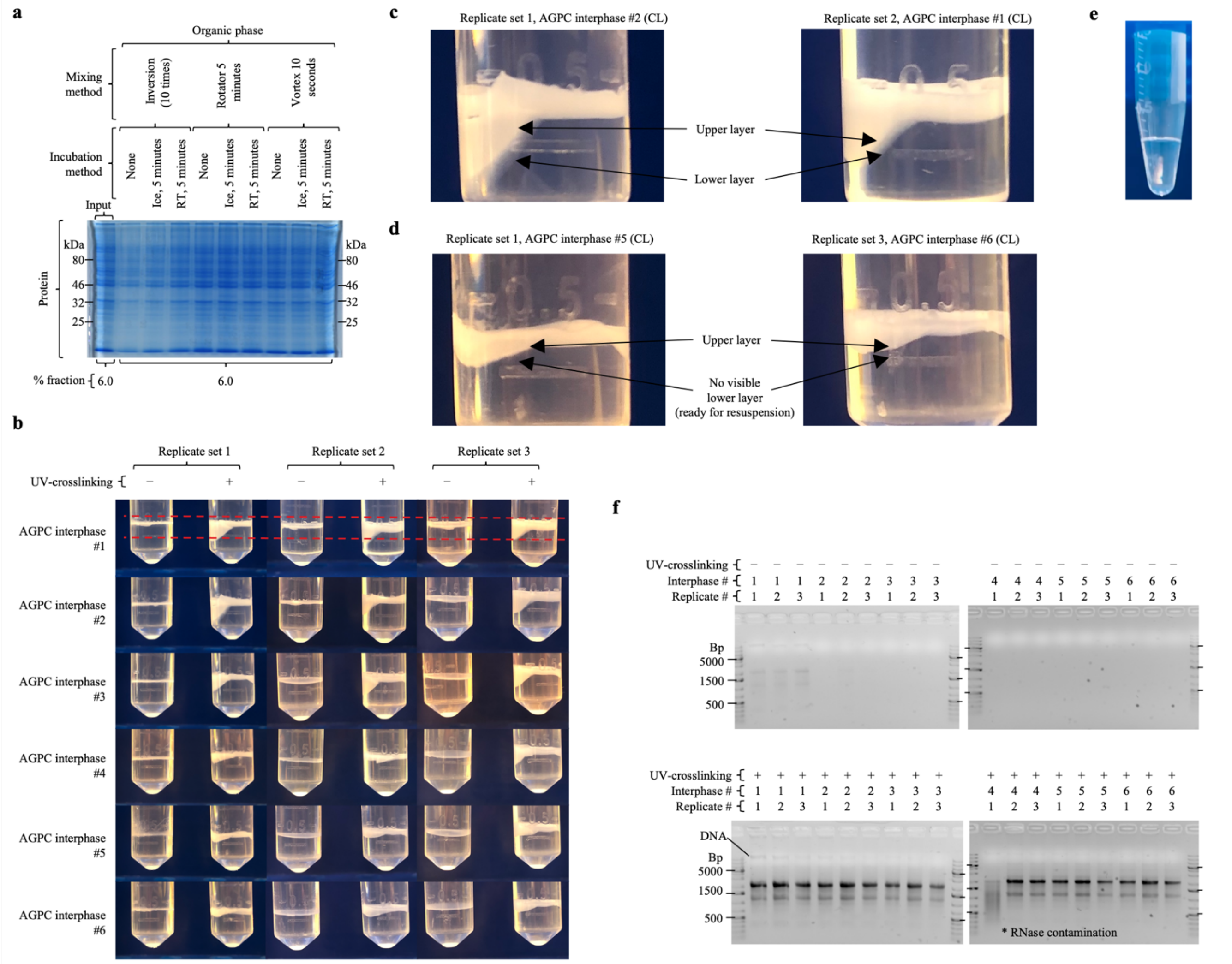

Supplementary Figure 10: Repeated AGPC extraction concentrates clRNPs. A/NGPC: acidic/neutral guanidinium thiocyanate-phenol-chloroform; AGP: acidic guanidinium thiocyanate-phenol. **a-g** Analytical summary: AGP input suspensions contained either 10 million UV-crosslinked (0.4 J/cm^2, 254 nm) or 10 million non-crosslinked HeLa cells. Additional information on sample composition, conditions, and normalization is indicated in the figure panels. **a** Comparison of methanol-precipitated organic phase fractions normalized to % fraction by SDS-PAGE and Coomassie Blue (protein) staining. **b** Pictorial representation of the AGPC interphase following up to six AGPC extractions of UV-crosslinked and non-crosslinked samples from Fig. 1b, c. **c** Select images from panel **b** with arrows pointing to the solid upper and dispersed lower layer of the AGPC interphase. **d** Select images from panel **b** with arrows pointing to the upper and depleted lower layer of the AGPC interphase. **e** Pictorial representation of the final AGPC interphase resuspended in fresh AGP. **f** TBE gel analysis on RNA (equivalent % fraction) isolated by NGPC extractions of proteinase K treated AGPC interphase samples from panel **b** (i.e., Fig. 1b, c); parallel gels. Experiments (**a**, **f**) were performed once. Source data are provided as a Source Data file.

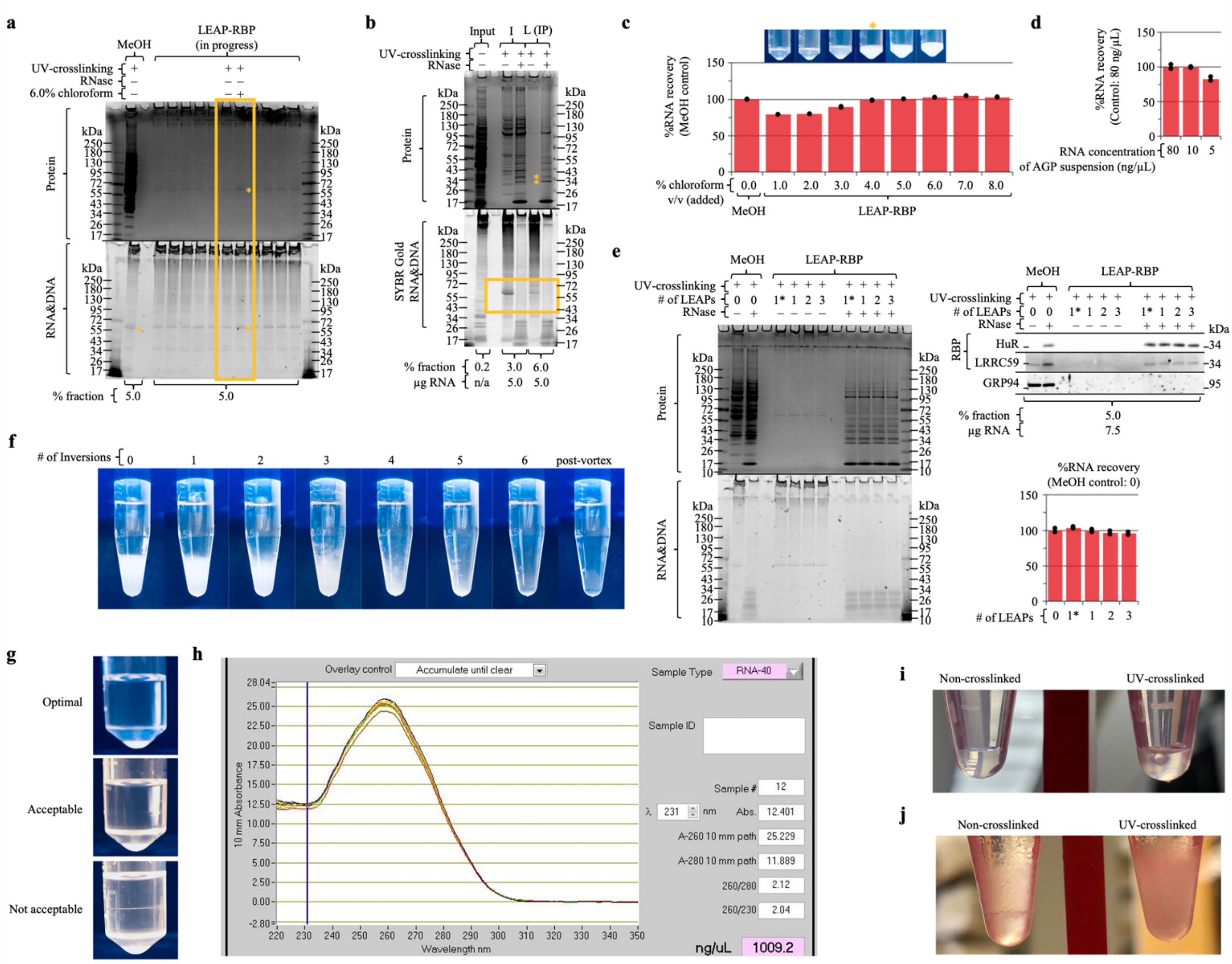

Supplementary Figure 11: Isolation of RNPs from AGP suspensions with LEAP-RBP. AGPC: acidic guanidinium thiocyanate-phenol-chloroform; AGP: acidic guanidinium thiocyanate-phenol; SRA: SRA analysis; CL: of 10 million UV-crosslinked (0.4 J/cm^2, 254 nm); nCL: of 10 million non-crosslinked HeLa cells. **a**-**j** Analytical summary: RNA (UV-spectrophotometry) and protein (BCA) quantitation data shown in bar charts represents the mean of two independent samples. Additional information on sample composition, conditions, and normalization is indicated in the figure panels. **a** Comparison of untreated LEAP-RBP (in progress) fractions isolated from final AGPC interphase suspensions (CL) by SRA and Coomassie Blue (protein), SYBR Safe (RNA&DNA) staining. **b** Comparison of INP and LEAP-RBP (in progress) fractions isolated from final AGPC interphase suspensions (CL) by SRA and Coomassie Blue (protein), SYBR Gold (RNA&DNA) staining. **c** Bar chart showing the effect of chloroform concentration on %RNA recovery from final AGPC interphase suspensions (CL). Pictorial representations illustrating the qualitative change in sample appearance. **d** RNA recovery by LEAP-RBP from AGP suspensions containing varying amounts of protein-bound RNA. **e** %RNA recovery and comparison of RNP fractions isolated by methanol precipitation or 1–3 LEAP steps. **f** Pictorial representations illustrating sample appearance after each inversion during the LEAP-RBP method. **g** Pictorial representations illustrating sample appearance following centrifugation of LEAP-RBP samples using optimal, acceptable, or not acceptable (excessive) volumes of chloroform. **h** UV-spectrophotometric profile of LEAP-RBP fractions isolated from final AGPC interphase suspensions (CL) using 1–3 methanol rinse steps. **i**, **j** Pictorial representations illustrating appearance of LEAP-RBP fractions isolated from AGP input suspensions (CL or nCL) during DNA depletion (**i**) and subsequent LEAP-RBP step (**j**). Experiments were performed once (**d**), twice (**b**), or three times with similar results (**a**, **e**). Source data are provided as a Source Data file.

Supplementary Note 2
This note provides supporting information and technical considerations for the SDS-PAGE RNase-sensitivity Assay.

2a. Validation and technical considerations for RNase-digestion of clRNPs.
Comparison of RNase-treated and untreated clRNPs by SDS-PAGE is a simple and cost-effective method for identification of RBPs based on their RNase-sensitivite mobility in SDS-PAGE. However, the sensitivity, accuracy, and reproducibility of SRA depends both on the quality of the clRNP isolation method and the SRA conditions themselves. See Supplementary Note 1a-c and Supplementary Methods for suggestions and technical information regarding isolation of clRNPs by LEAP-RBP. RNase-digestion reactions were performed in thermocycler tubes as 10 or 12 µL reactions with 4 µL of 1% LiDS TE suspended samples. RNA integrity is maintained in untreated samples at 37 ˚C and 1% LiDS TE does not inhibit RNase when using the recommended digest conditions (red arrow; Supplementary Fig. 12a, b, Methods). LEAP-RBP fractions isolated from AGP input (RNPs), or final AGPC interphase (clRNPs) suspensions were resuspended in 1% LiDS TE, quantified, diluted, and clarified for SRA analysis as described in Supplementary Methods (Supplementary Fig. 12b). Sample tubes were centrifuged briefly with a mini centrifuge prior to adding RNase-digestion components. Master mixes containing either untreated or RNase-digestion components were added to samples (Supplementary Fig. 10b). Samples were briefly vortexed at medium setting and centrifuged again after adding master mixes or 5X LB WS for SDS-PAGE. Samples were stored at −80˚C or used immediately for SDS-PAGE. Frozen samples were thawed and incubated at RT for 30 min prior to denaturation.

2b. Technical considerations for identification of RNase-sensitive RBPs by SRA and immunoblot.
Confident detection of RNase-sensitive RBPs requires protein bands in RNase-treated and untreated samples to have comparable dimensions. Therefore, efforts were made to avoid artifacts that lead to lane narrowing or widening during SDS-PAGE of RNPs; this is mainly an issue when analyzing untreated samples. Following sample denaturation (Methods), samples were quickly moved and centrifuged briefly with a mini centrifuge (3 sec), vortexed for 2 sec at medium setting and centrifuged again before being placed on ice. When adding RNase treated and untreated samples in neighboring lanes, samples were added in one direction across the gel (Fig. 1b); alternating wells allowed samples from one well to diffuse towards neighboring wells before they were filled leading to lane narrowing artifacts (Supplementary Fig. 12c). Loading RNase treated or untreated samples containing >5 µg of RNA in adjacent wells results in separation artifacts (Supplementary Fig. 12d); this can lead to false apparent RNase-sensitivity. Therefore, RNase-treated and untreated samples were loaded in one of two configurations: alternating with gaps (Source Data Fig. 2a, Source Data Fig. 3a), or segregated clustering (Supplementary Figure Fig. 6b, c, 11e). When using the latter configuration, loading RNP fractions with large differences in RNA and/or protein composition adjacent to each other led to lane-narrowing artifacts (Supplementary Fig. 12e).

2c. SDS-PAGE and transfer conditions for SRA and immunoblot.
The composition of the polyacrylamide gel or SDS-PAGE and transfer conditions can affect SRA results. The transfer conditions used in this study were optimized to work for proteins ranging from ~28–180 kDa. After transferring proteins to membranes, polyacrylamide gels were Coomassie Blue stained to assess transfer efficiency. Protein enrichment in RNP fractions was assessed by including input samples containing an equivalent amount of protein (µg) as RNP samples. If the protein of interest was detected in input samples, then it was assumed SDS-PAGE and transfer conditions would allow detection of the same protein in RNase-treated RNP fractions. Gradient polyacrylamide gels were necessary to ensure efficient transfer and simultaneous assessment of RBPs with different molecular weights.

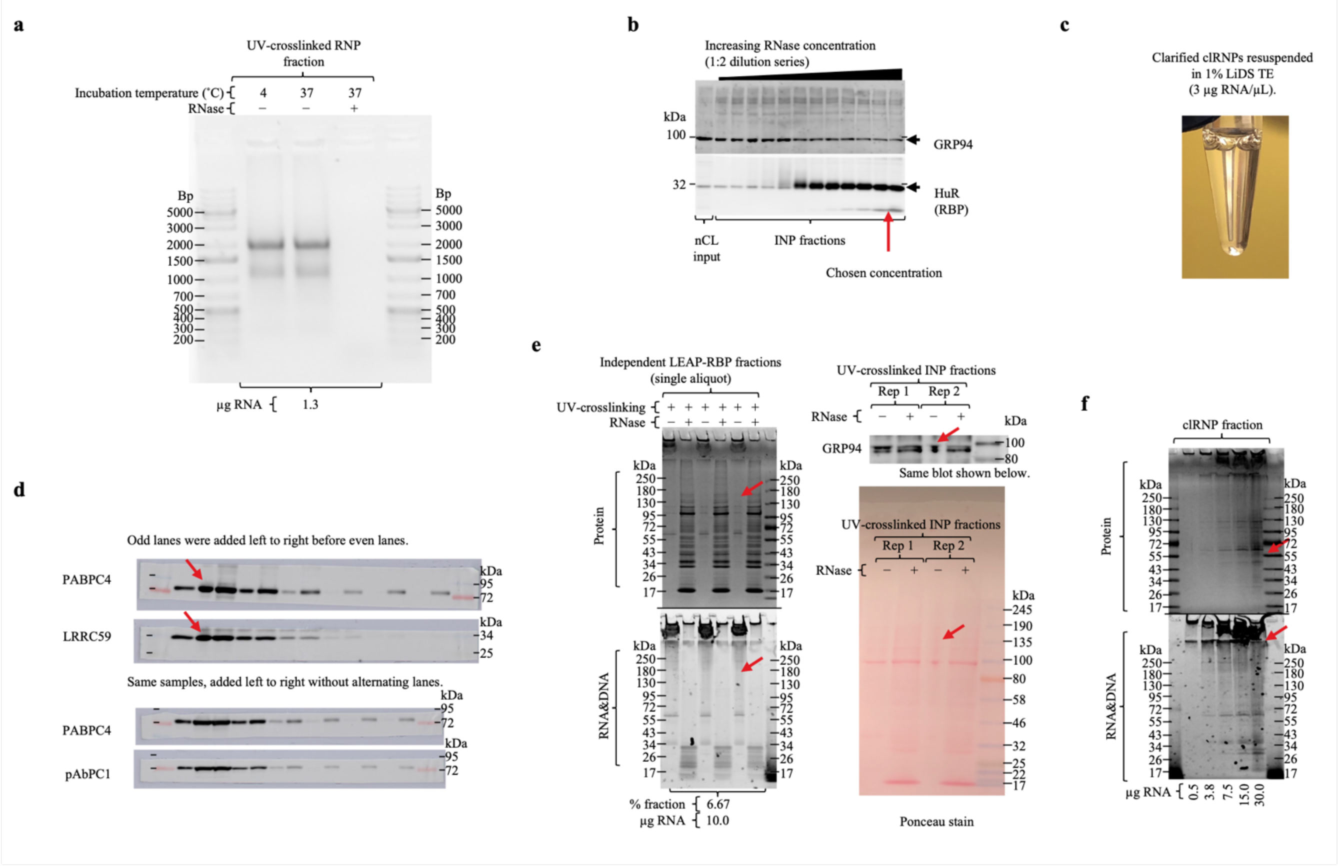

Supplementary Figure 12: Validation of RNase-digestion conditions and technical considerations for SDS-PAGE RNase-sensitivity Assay (SRA). AGPC: acidic guanidinium thiocyanate-phenol-chloroform; AGP: acidic guanidinium thiocyanate-phenol; SRA: SRA analysis. CL: of 10 million UV-crosslinked (0.4 J/cm^2, 254 nm). **a**-**f** Additional information on sample composition, conditions, and normalization is indicated in the figure panels. **a** TBE gel analysis on RNase-treated or untreated RNP fractions isolated from AGP input suspensions with LEAP-RBP and DNA depletion step; RNase digests (−/+) were performed on 4 µL 1% LiDS TE sample suspension in 10 µL format as described in the Methods and diluted 1:3 with TE buffer prior to loading. **b** Comparison of INP fractions isolated from final AGPC interphase suspensions (CL) and treated with increasing amounts of RNase cocktail (Ambion) by SDS-PAGE. **c** Pictorial representation illustrating samples appearance of clarified clRNPs in 1% LiDS TE, isolated by LEAP-RBP from final AGPC interphase suspensions (CL). **d** Representative immunoblots illustrating lane narrowing artifacts caused by improper sample loading. **e** Comparison of replicate LEAP-RBP fractions isolated from final AGPC interphase suspensions (CL) by SRA and Coomassie Blue (protein), SYBR Safe (RNA&DNA) staining, or immunoblot. Red arrows point to lane narrowing artifacts caused by improper sample configurations. **f** Comparison of LEAP-RBP fractions isolated from AGP input suspensions (CL) by SRA and Coomassie Blue (protein), SYBR Safe (RNA&DNA) staining. Red arrows point to lane narrowing artifacts caused by improper sample configuration and/or overloading of samples. Experiments were performed once (**a**, **b**), or three times (**d**-**f**) with similar results. Source data are provided as a Source Data file.

Supplementary Note 3
This note provides supporting information for estimating RNA, protein, and RBP-specific UV-crosslinking efficiencies.

3a. LEAP-RBP allows direct quantitative measurement of total RNA and protein UV-crosslinking efficiency.
UV-crosslinking conditions which maximized the amount of material (μg protein-bound RNA/total RNA) were selected for development of LEAP-RBP. 0.4 J/cm^2 (254 nm) maximized free RNA depletion from the aqueous phase during AGPC extraction (~75–80%) ([13]; Fig.1b). While indirect, the data suggests UV-irradiating cells with 0.4 J/cm^2 (254 nm) crosslinks roughly 75–80% of total RNA to protein. The fraction of RNA that is UV-crosslinked to protein can be used to measure UV-crosslinking efficiency of RNA. Hypothetically, LEAP-RBP and repeated AGPC extraction can directly measure RNA UV-crosslinking efficiency by allowing simultaneous isolation of total protein-bound RNA and total RNA from UV-irradiated samples (RNA UV-crosslinking efficiency = total protein-bound RNA/total RNA * 100; Supplementary Fig. 13a). However, for this to be an accurate assessment, three prerequisites must be met: 1) repeated AGPC extractions must deplete free RNA and maintain protein-bound RNA with near 100% efficiency; 2) LEAP-RBP must recover near 100% of protein-bound and unbound RNA species; 3) DNA must be efficiently depleted without compromising the near 100% recovery of protein-bound and unbound RNA species. For these purposes, near 100% RNA recovery refers to a non-significant difference in RNA yield (μg RNA/% fraction) as quantified by UV-spectrophotometry when compared to a suitable control. Multiple lines of evidence (**below**) support that these prerequisites have been met.

Free RNA is efficiently depleted during repeated AGPC extractions. Two repeated AGPC extractions were sufficient to fully deplete RNA at the AGPC interphase of non-crosslinked cells as quantified by UV-spectrophotometry or analyzed by SYBR Safe (RNA&DNA) staining of AGPC interphase samples separated by SDS-PAGE and TBE (Fig. 1b, Supplementary Fig. 2f). These data demonstrate that free RNA is rapidly depleted during repeated AGPC extraction and is consistent with previously published findings [13].

LEAP-RBP recovers near 100% of protein-bound RNA from final AGPC interphase suspensions. No significant differences in RNA recovery were detected between LEAP-RBP and INP fractions isolated from final AGPC interphase suspensions and methanol (95% v/v) precipitated AGPC interphase samples by UV-spectrophotometry (Fig. 2a). INP is a more traditional RNA-precipitation method (isopropanol and NaCl) utilizing lengthier incubation times and co-precipitants (GlycoBlue) for efficient RNA-centric isolation of protein-bound RNA. Methanol is a highly efficient protein-centric method for orthogonal assessment of RNA-bound protein recovery by INP and LEAP-RBP (Methods). Methanol precipitated LEAP-RBP supernatants do not contain quantifiable RNA by UV-spectrophotometry and are devoid of RNase-sensitive proteins by SRA and Coomassie Blue (protein) staining (Supplementary Fig. 3a); this includes the ~65 kD clRNP indicative of small RNA-bound protein recovery SYBR Safe (RNA&DNA) stained gel (Fig. 3a, Supplementary Fig. 6b, Supplementary Fig. 11a, b). Lastly, LEAP-RBP fractions isolated from final AGPC interphase suspensions of UV-crosslinked cells can be subjected to 2 additional LEAP steps without a statistically significant decrease in RNA yield as quantified by UV-spectrophotometry, one-way ANOVA, $F(3, 4) = 0.73$, $p = .584$ (Supplementary Fig. 3b), or apparent change in RNase-sensitive protein profiles evaluated by SRA with Coomassie Blue (protein) staining (Supplementary Fig. 11e).

LEAP-RBP recovers near 100% of protein-bound and unbound RNA species from AGP input suspensions. LEAP-RBP was performed on AGP input suspensions (without repeated AGPC extraction) containing equivalent amounts of UV-crosslinked or non-crosslinked cells and RNA yield was quantified by UV-spectrophotometry (Fig. 3a). No significant effect of UV-crosslinking on RNA yield (with or without DNA depletion step) was detected demonstrating unbiased recovery of protein-bound and unbound RNA species by LEAP-RBP; two-way ANOVA: non-significant main effect of UV-crosslinking on RNA yield, ($F(1, 8) = 0.59$, $p = .463$. This finding was further validated in a UV-dose response experiment and one-way ANOVA to analyze the effect of UV-dose (independent variable) on RNA yield (dependent variable) of LEAP-RBP w/ DNA depletion step: non-significant main effect of UV-dose (0.0, 0.1, 0.2, 0.4, and 0.8 J/cm^2; 254 nm) on RNA yield (Supplementary Fig. 6a), $F(4, 5) = 0.89$, $p = .534$.

RNA-bound proteins exhibit efficient partitioning to the AGPC interphase. AGPC interphase samples isolated by methanol (95% v/v) precipitation following up to 6 AGPC extractions display comparable RNase-sensitive protein profiles by SRA and immunoblot (Fig. 1c). Furthermore, LEAP-RBP fractions isolated from AGP input and final AGPC interphase suspensions from the same UV-crosslinked sample display comparable RNase-sensitive protein profiles and signal recovery by SRA with Coomassie Blue (protein) staining or immunoblot (Fig. 3a, b). These data indicate that RNA-bound proteins exhibit unbiased partitioning to the AGPC interphase and are efficiently maintained during repeated AGPC extractions.

The LEAP-RBP DNA depletion step efficiently depletes DNA contaminants. Comparisons of DNase-treated and untreated samples by SRA shows near-complete depletion of RNase-insensitive SYBR Safe (RNA&DNA) stained species in the stacker of polyacrylamide gels during SDS-PAGE (Supplementary Fig. 5b). The DNA depletion step removed >99% of DNA present in LEAP-RBP fractions isolated from final AGPC interphase suspensions of UV-crosslinked cells as quantified by qPCR (Supplementary Fig. 5b). These data indicate that the SYBR Safe stained RNase-insensitive species in the stacker of polyacrylamide gels during SDS-PAGE is DNA; their higher intensity in LEAP-RBP fractions isolated from AGP input suspensions compared to final AGPC interphase suspensions supports this assessment (Fig. 3a) as repeated AGPC extraction depletes DNA relative to RNA (Supplementary Fig. 10f).

The second LEAP step following the DNA depletion step recovers near 100% of protein-bound and unbound RNA species. AGP input suspensions containing UV-crosslinked or non-crosslinked cells were subjected to LEAP-RBP with or without the DNA depletion step which includes a second LEAP step; RNA yield (dependent variable) was quantified by UV-spectrophotometry and analyzed by two-way ANOVA with DNA depletion and UV-crosslinking status as the independent variables (Fig. 3a); non-significant interaction between UV-crosslinking status and DNA depletion step on RNA yield, $F(1, 8) = 0.01$, $p = .915$; non-significant main effect of UV-crosslinking status on RNA yield, $F(1, 8) = 0.59$, $p = .463$. However, there was a statistically significant main effect of DNA depletion step on RNA yield; $F(1, 8) = 294.98$, $p < .001$. DNA contamination contributes to overall absorbance values during quantitation of RNA yield (UV-spectrophotometry). Therefore, the effect of DNA depletion step on RNA yield should not be taken as a decrease in RNA recovery. As evidence of this, comparison of these fractions by SRA with Coomassie Blue (protein) staining or immunoblot shows comparable RNase-sensitive protein profiles despite differences in RNA yield as quantified by UV-spectrophotometry (nanodrop) (Fig. 3a, c). The non-significant main effect of UV-crosslinking status on RNA yield indicates LEAP-RBP recovers both protein-bound and unbound RNA species in an unbiased manner, $F(1, 8) = 0.59$, $p = .463$.

Cumulatively, these data demonstrate that comparing the RNA yield of LEAP-RBP from AGP input suspensions with DNA depletion step (total RNA) and final AGPC interphase suspension with or without DNA depletion step (total protein-bound RNA) allows accurate and direct assessment of RNA UV-crosslinking efficiency (Supplementary Fig. 13a). Using this approach, UV-irradiating HeLa cells with 0.4 J/cm^2 (254 nm) crosslinks ~70% of RNA species (Fig. 3a, b). These results are close to indirect estimates obtained by the OOPs method (75%–80%) [13]. While total RNA yield was not quantitated for other experiments in this study, the ratio of total RNA/total protein (~0.11 mg RNA/1.0 mg total protein) from the experiment above (Fig. 3a, b) can be used to make a rough estimate for other experiments (Supplementary Fig. 13b): Fig. 2a: 77.32% (HeLa); Supplementary Fig. 8c: 79.7% (HeLa), 73.6% (293T), 78.4% (Huh7), and 70.3% (832/13). Alternatively, RNA UV-crosslinking efficiency were estimated by dividing the RNA/protein ratio of RNP fractions (i.e., RNP composition) by the RNA/protein ratio of corresponding clRNP fractions. This was demonstrated to be a valid approach for correction of signal loss which may occur during repeated AGPC extractions if both fractions display comparable RNA-bound protein profiles by SRA with Coomassie Blue (protein) staining when normalized to µg of protein (strategy is detailed in the provided Source Date for Supplementary Fig. 8b, d-g). Protein UV-crosslinking efficiency refers to the % of total protein that is recovered as RNA-bound (protein UV-crosslinking efficiency = total RNA-bound protein/total protein * 100). Roughly 91% of the protein in LEAP-RBP fractions isolated from final AGPC interphase suspensions is estimated to be RNA-bound by SILAC LC-MS/MS analysis (Fig. 5g, h). No significant difference in protein yield was detected between LEAP-RBP fractions isolated from AGP input (with DNA depletion step) and

final AGPC interphase suspensions (Fig. 3a), Fisher's PLSD (two-tailed, unpaired, homoscedastic t test), $p = .998$. Cumulatively, these data suggest that comparing the protein yield of LEAP-RBP from AGP input suspensions with DNA depletion step or final AGPC interphase with or without DNA depletion (total RNA-bound protein), and methanol (95% v/v) precipitated input samples (total protein), provides a good approximation of protein UV-crosslinking efficiency (Supplementary Fig. 13a). However, comparing protein yields of LEAP-RBP from AGP input suspensions with DNA depletion step and methanol (95% v/v) precipitated input samples (total protein) is the preferred approach for reasons noted above. Compared to estimated RNA UV-crosslinking efficiencies, estimated protein UV-crosslinking efficiencies are considerably lower (Supplementary Fig. 13c). However, this is somewhat expected: not all proteins interact with RNA, but all RNA species are expected to contain one or more protein interactors. From this perspective, the difference between RNA and protein UV-crosslinking efficiencies is caused by the inclusion of proteins that don't interact with RNA.

3b. LEAP-RBP allows rapid and comprehensive assessment of UV-crosslinking conditions.

Together, RNA and protein UV-crosslinking efficiencies provide a way to evaluate UV-crosslinking conditions. Because these metrics are normalized to total RNA and protein yields, a relative assessment can be made by comparing LEAP-RBP fractions containing total protein-bound RNA and/or total clRNPs isolated from replicate samples subjected to different UV-crosslinking conditions. As an example, 10 cm plates containing ~10 million HeLa cells were UV-crosslinked with 0.1, 0.2, 0.4, and 0.8 J/cm^2 (254 nm) and total clRNPs were isolated from final AGPC interphase suspensions. The effect of UV-dose on RNA UV-crosslinking efficiency was evaluated by comparing RNA yields (Supplementary Fig. 13d). As expected, maximum RNA UV-crosslinking efficiency was obtained by UV-irradiating cells with at least 0.4 J/cm^2 (254 nm) (gold box) [13]. As another example, LEAP-RPP fractions (with DNA depletion step) were isolated from AGP input suspensions containing equal amounts of HeLa cells UV-irradiated with 0.0, 0.1, 0.2, 0.4, and 0.8 J/cm^2 (254 nm). The effect of UV-dose on protein UV-crosslinking efficiency was evaluated by comparing protein yields (Extended Data Fig. 6a). Unlike RNA UV-crosslinking efficiency, maximum protein UV-crosslinking efficiency was obtained by UV-irradiating cells with 0.8 J/cm^2 (254 nm); protein UV-crosslinking efficiency was estimated as a percentage of total protein for comparison.

For the experiments in this study, cells were washed twice with ice-cold PBS and UV-crosslinked on ice to remove media components which might interfere with UV-crosslinking and to prevent excessive heating of samples, respectively. However, the effect of these sample preparation measures on UV-crosslinking efficiency has not been evaluated in the way that is afforded by LEAP-RBP. Therefore, 10 cm plates containing ~10 million HeLa cells were UV-irradiated with 0.4 J/cm^2 (254 nm) with or without media removal and/or on or off ice and/or with or without ice-cold PBS washes and/or with or without extended incubation on ice (15 minutes) (Supplementary Fig. 13e). Maximum RNA UV-crosslinking efficiencies were obtained using the prescribed sample preparation method (gold box). Interestingly, placing cells on ice during UV-irradiation increases RNA UV-crosslinking efficiency with or without media-removal. RNase-sensitive protein profiles appear similar by SRA and Coomassie Blue (protein) staining but differ in total intensity. However, proper evaluation would require more thorough analysis by SRA and immunoblot as shown in Supplementary Fig. 6c. These results suggest consistent sample processing and UV-crosslinking conditions are necessary for reproducible results. Inconsistent sample processing and UV-crosslinking (i.e., batch effects) impact RBP-specific UV-crosslinking efficiency and change the amount of total RNA-bound protein in starting samples (total $|S|$). The effects of UV-crosslinking conditions and/or efficiencies on the ability to detect $\Delta\log_2(S)$ should be considered (i.e., those that potentially effect dynamic range).

3c. RBP-specific UV-crosslinking efficiencies as a reproducible metric for RBP studies.

The UV-crosslinking efficiencies of individual RBPs range from less than 0.3% for non-canonical RBPs such as RPN1, and upwards of ~20% for canonical RBPs such as nucleolin (NCL) or HuR (Supplementary Fig. 2a). The estimated protein UV-crosslinking efficiency for HeLa cells UV-irradiated with 0.4 J/cm^2 (254 nm) is estimated between 1.5–2.0% (Supplementary Fig. 6a, 13c). For SRA analysis, input samples containing total protein and LEAP-RBP fractions representative of total clRNPs are normalized to µg of protein (~50x % of LEAP-RBP fraction vs % of input samples) for evaluation of protein enrichment. Because the UV-crosslinking efficiency of XRN1 is comparable to protein UV-crosslinking efficiency under these conditions (Supplementary Fig. 2a), it displays comparable intensity in both fractions normalized in this way (Supplementary Fig. 13f). Curiously, RBP-specific UV-crosslinking efficiencies were found to vary in their dose-responsiveness (Supplementary Fig. 6c). We speculate this is caused by differences in their RNA-binding mechanisms and/or kinetics *in vivo* [10, 14-17]; however, the mechanism of UV-crosslinking in macromolecular systems and how to interpret changes in RNA-bound protein abundance are still poorly understood [17-20]. These findings illustrate the importance of consistent UV-crosslinking practices for generation of reproducible data and demonstrate the ability of LEAP-RBP to provide comprehensive assessment of UV-crosslinking conditions.

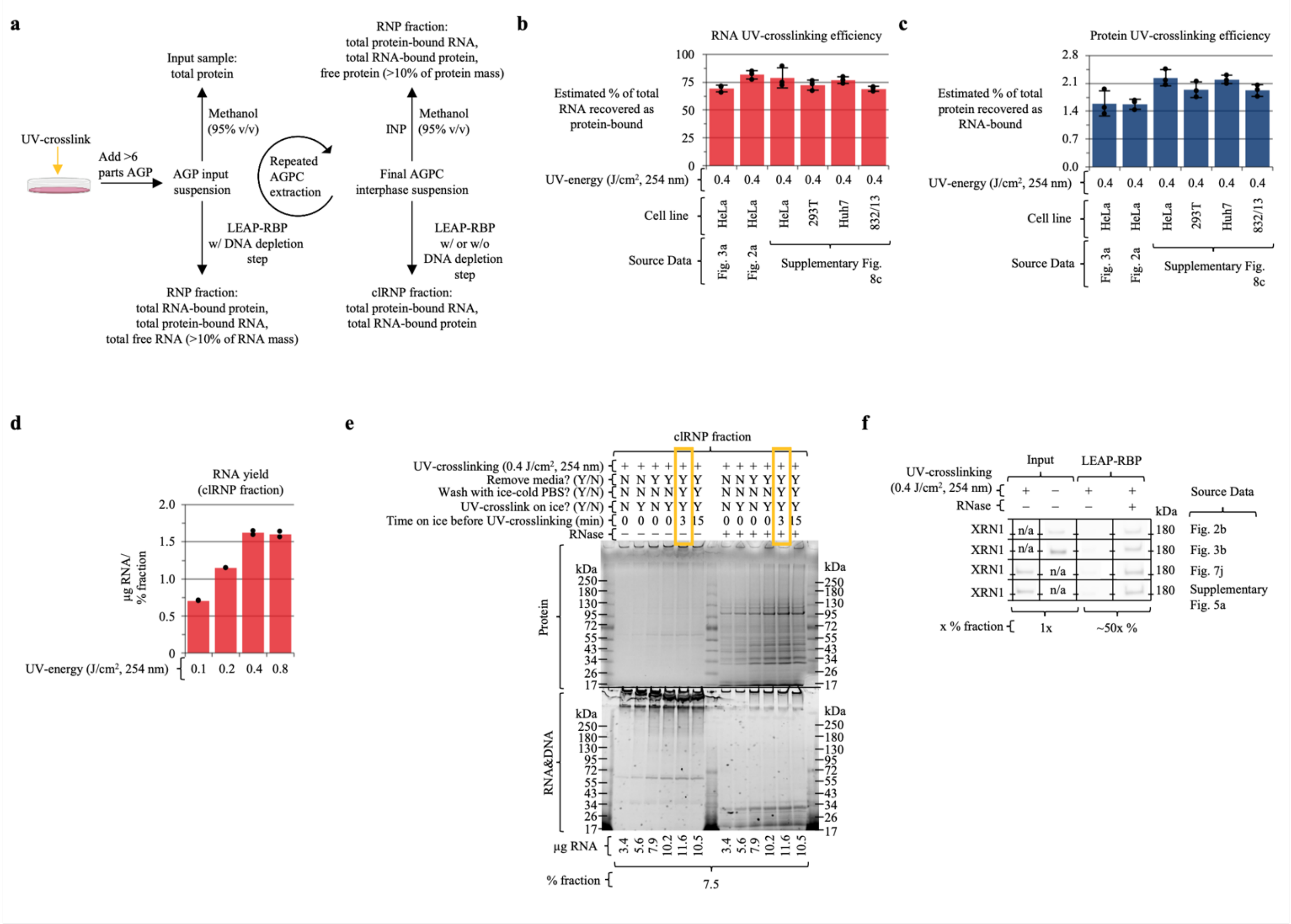

Supplementary Figure 13: Evaluation of UV-crosslinking conditions and estimating RNA and protein UV-crosslinking efficiencies with the LEAP-RBP method. AGPC: acidic guanidinium thiocyanate-phenol-chloroform; AGP: acidic guanidinium thiocyanate-phenol; SRA: SRA analysis. **a** Graphical representation depicting the RNA populations recovered by LEAP-RBP from the corresponding AGP input or final AGPC interphase suspensions of UV-crosslinked samples. Graphic was prepared in BioRender. **b**, **c** Bart charts showing estimated RNA UV-crosslinking efficiencies (**b**) or protein UV-crosslinking efficiency (**c**) of cellular samples from different experiments presented in this study. Protein (BCA) and RNA (UV-spectrophotometry) quantitation data shown in bar charts represents the mean ± *SD* of 3 biologically independent samples. **d** Bar chart showing RNA yield of clRNP fractions isolated from final AGPC interphase of 10 million HeLa cells irradiated with increasing UV-energy (254 nm). RNA (UV-spectrophotometry) quantitation data shown in bar chart represents the mean of 2 independent replicates isolated from the same final AGPC interphase suspension (n = 1 biologically independent sample for each UV-dose). **d** Comparison of RNA yields between LEAP-RBP fractions isolated from final AGPC interphase suspensions of cells irradiated with varying doses of UV-energy (254 nm). **e** Comparison of LEAP-RBP fractions isolated from final AGPC interphase suspensions of cells irradiated under different UV-crosslinking conditions by SRA; sample composition indicated in figure panel (n = 1 biologically independent sample). **f** Select immunoblots from referenced figures demonstrating comparable UV-enrichment of XRN1 in LEAP-RBP fractions. Experiments were performed once (**d**, **e**), see referenced experiments for panels **b**, **c**, **f**. Source data are provided as a Source Data file.

Supplementary Note 4
This note provides additional observations and rationale for distinguishing UV-dependent enrichment of free protein and signal-dependent recovery of noise. Supporting documentation illustrating the importance of method robustness and specificity for comparative studies is also included.

4a. UV-dependent enrichment of free protein vs signal-dependent recovery of noise.
UV-dependent enrichment of free protein refers to increased recovery of free RBPs and non-RBPs from UV-crosslinked samples as compared to non-crosslinked controls. Signal-dependent recovery of noise refers to the recovery of unbound proteins during RNA-centric enrichment of their RNA-bound counterparts. Both are observable by SDS-PAGE when comparing equivalent amounts (% fraction) of RNase-treated and untreated RNP fractions isolated from UV-crosslinked and non-crosslinked samples (Supplementary Fig. 14a). Increased recovery of RNase-insensitive protein is considered UV-dependent enrichment of free protein (red and blue boxes). UV-dependent recovery of unbound RBPs (noise) which is dependent on the presence of RNA-bound protein (signal) is considered signal-dependent recovery of noise (red box).

4b. UV-dependent enrichment of free protein is a widely observed phenomenon.
UV-dependent enrichment of free protein has been noted by others but the explanation for its occurrence has varied. In certain situations, UV-dependent enrichment of free protein appears to be a technical artifact. For example, when performing AGPC extraction on UV-crosslinked and non-crosslinked cells, the AGPC interphase of UV-crosslinked samples is larger than that of non-crosslinked cells (Supplementary Fig. 10b). The authors of the AGPC-based method, XRNAX, reported the same observation [5]. These authors also reported that the AGPC interphase of non-crosslinked samples is “fluid-like” whereas the AGPC interphase of UV-crosslinked samples is “one sticky blob in addition to the fluid-like regular interphase contents”. Continuing, “this blob and its fluid-like surrounding are not clearly distinct but seem more like a sponge, which soaks up the fluid it sits in” (https://www.xrnax.com/faq/2018/12/30/interphase-1-why-have-a-background-control-why-silica-enrichment-after-xrnax). Noting the significance of this observation, these authors emphasized the need for SILAC-based approach to avoid underestimating free protein recovered from UV-crosslinked samples [5]. In this instance, UV-dependent enrichment of free protein is thought to be an artifact caused by UV-dependent sample physical properties. However, while this likely contributes to the UV-dependent enrichment of free protein at the first AGPC interphase, repeated AGPC extraction would be expected to reduce or eliminate this protein fraction. For some RNase-insensitive proteins (e.g., β-tubulin and GAPDH), this appears to be true (Fig. 1c). For others (e.g., GRP94 or RPN1), the amount of free protein at the AGPC interphase reaches an apparent UV-dependent enrichment limit, resulting in the apparent UV-enrichment of “RNase-insensitive” or free proteins at the final AGPC interphase (Fig. 1c).

Similar observations were also reported by the authors of the OOPs method which utilizes 3 AGPC extractions as part of their methodological approach [13]. In this method, interphase samples are precipitated with methanol following 3 AGPC extractions, RNase-treated, and subjected to a fourth AGPC extraction; untreated samples are processed in parallel as a control. Proteins are methanol precipitated from the organic phases and RBPs are then identified by their RNase-dependent enrichment in the organic phase. Roughly 96% of proteins were found to exhibit RNase-dependent enrichment in the 4th organic phase ([13]; Fig. 2i). These authors also noted that “Proteins that migrated to the organic phase included those that were CL independent, suggesting their presence in the interface is RNA dependent, but their interaction with RNA was stable even in the absence of CL” ([13]; Fig. 2f, Supplementary Fig. 2i). Here, UV-dependent enrichment of crosslink-independent (free protein) at the AGPC interphase is thought to be a result of stable interactions with the RNA components of protein-bound RNA at the AGPC interphase. Alternatively, we postulated that insoluble clRNPs at the AGPC interphase might provide additional interaction surfaces for association of free protein. A prediction from this hypothesis is that the amount of protein recovered from the AGPC interphase suspensions is dependent on the amount of RNA-bound protein (total $|S|$), rather then the starting amount of protein. To test this prediction, samples containing 10 million UV-crosslinked (0.4 J/cm^2, 254 nm) or 10 million UV-crosslinked and 10 million non-crosslinked HeLa cells were subjected to 6 AGPC extractions. While the AGPC interphase of the pooled samples were larger than the non-pooled samples during the initial few AGPC extractions, they were of comparable size by the final AGPC extraction. Following precipitation of protein-bound RNA by INP, RNA and protein yields were quantified by UV-spectrophotometry and BCA respectively. No discernible

differences in RNA or protein recovery were observed and both fractions displayed comparable signal recovery (e.g., XRN1, HuR, and RPN1) and free protein recovery (e.g., GRP94) by SRA and immunoblot (Supplementary Fig. 14b). These results suggest UV-dependent enrichment of free protein at the AGPC interphase is dependent on the amount of RNA-bound protein in the sample (total $|S|$), not the amount of total protein in starting samples.

4c. High method specificity for RNA-bound RBPs (high %TP$_S$) reveals signal-dependent recovery of noise.

After development of LEAP-RBP, it was realized that UV-dependent enrichment of free protein at the AGPC interphase was not an isolated phenomenon. As shown in Fig. 3a, b and Supplementary Fig. 6a-c, performing LEAP-RBP (with DNA depletion step) on AGP input suspensions containing UV-crosslinked or non-crosslinked cells resulted in UV-dependent recovery of unbound RBPs despite comparable (UV-independent) recovery of RNA. Therefore, UV-dependent enrichment of unbound RBPs was dependent on RNA-bound protein, not RNA itself. These results thus appeared to invalidate both prior explanations for UV-dependent enrichment of free protein at the AGPC interphase. For example, it was proposed that UV-enrichment of non-crosslinked protein at the AGPC interphase occurs through stable interactions with RNA which exhibits high UV-dependent enrichment at the AGPC interphase [13]. A direct prediction of this hypothesis is that if the presence of RNA at the AGPC interphase is UV-independent, then free protein would be recovered in equal quantities under the same conditions. The harsh chaotropic conditions used during the LEAP-RBP method are comparable to AGPC extraction. Furthermore, AGP input suspensions containing equal amounts of UV-crosslinked or non-crosslinked cells have comparable amounts of RNA. Yet, recovery of unbound RBPs is clearly UV-dependent despite comparable (UV-independent) recovery of RNA (Fig. 3a, b, Supplementary Fig. 6a, c). Alternatively, and as noted above, we hypothesized that insoluble clRNPs at the AGPC-interphase of UV-crosslinked cells provide additional interaction surfaces for co-precipitation of free protein resulting in their apparent UV-dependent recovery. However, the UV-dependent selective recovery of unbound RBPs (noise) over non-RBPs (background) from AGP input suspensions as shown in Fig. 3b alludes to a different phenomenon at work: RNA-bound protein (signal) -dependent recovery of noise. This phenomenon likely evaded prior recognition because repeated AGPC extractions and the INP method have insufficient specificity to distinguish RNase-insensitive RBPs with low S/N from non-RBPs which partition to the AGPC interphase (Fig. 1c, 2b).

The physicochemical basis for signal-dependent recovery of noise is enigmatic. The harsh chaotropic conditions of AGPC mixtures denatures RBPs and disrupts RNA-protein interactions preventing their effective RNA-dependent recovery from non-crosslinked samples. Yet, they are recovered in appreciable quantities from UV-crosslinked cells. Additionally, the recovery of free proteins via non-covalent interactions with RNA-bound proteins under the harsh conditions would, at most, be expected to result in non-selective recovery of free RBPs and non-RBPs. Yet, unbound RBPs are selectively recovered over non-RBPs.

4d. UV-dependent enrichment of free protein and signal-dependent recovery of noise operate under different rules.

Despite their apparent similarities, signal-dependent recovery of noise is more appropriate for describing UV- and signal-dependent phenomena than UV-dependent enrichment of free protein. These differences are illustrated in the following hypothetical example: Consider a starting population of various RBPs and non-RBPs in a cell. Upon UV-irradiation, some RBPs will be UV-crosslinked to RNA interactors but non-RBPs will not. Because UV-crosslinking is not 100% efficient, RBPs will comprise two populations: RNA-bound (signal) and unbound (noise) counterparts. Theoretically, RNA-centric enrichment methods will enrich RNA-bound proteins over their unbound counterparts (enhance S/N). However, RNA-centric enrichment methods, by definition, do not enrich unbound RBPs over unbound non-RBPs. Thus, UV-dependent enrichment of non-RBPs likely results from low method specificity, UV-dependent changes in sample physical properties, or non-specific UV-crosslinking. For example, UV-independent partitioning of the unbound RBP nucleolin (NCL) to the AGPC interphase and the inability of INP to discernibly enhance the S/N of NCL are examples of low method specificity (gold boxes; Fig. 1c, 2b). The apparent UV-dependent enrichment of RNase-insensitive protein (eg., GAPDH, GRP78, and β-tubulin) at the fourth AGPC interphase despite their eventual depletion is likely a result of UV-dependent changes in sample physical properties (Fig. 1c). Lastly, non-RBPs

which appear UV-enriched* by SILAC LC-MS/MS but remain undetectable or RNase-insensitive by SDS-PAGE and immunoblot are likely examples of low abundance non-specific UV-crosslinking. The experimental ideal would be a method capable of absolute specificity for RNA-bound proteins ($N = 0$), but this is not evident in current methodologies. Such a method would yield no detectable peptides from non-crosslinked samples during a SILAC-based LC-MS/MS experiment (Fig. 4a). Based on these principles, recovery of noise will always be observed during RNA-centric isolation of their RNA-bound counterparts while enrichment of background protein is not guaranteed. Consequently, when evaluating methods with varying $\%TP_S$ but comparable recovery of RNA-bound protein, background proteins will often go from observable to undetectable while noise simply varies in abundance.

Comparison of proteins identified by SILAC LC-MS/MS analysis of INP and LEAP-RBP demonstrates the difference between background and noise. For example, the INP method recovers many background proteins displaying $\log_2$(CL/nCL) ratios around 0 (Fig. 4f); comparatively, the LEAP-RBP method identified little to no background proteins, as these would display a similar distribution with mean $\log_2$(CL/nCL) ratio of 0. As mentioned previously, background proteins are expected to go from observable to undetectable with increasing $\%TP_S$. Indeed, 392 proteins displaying $\log_2$(CL/nCL) ratios with a mean distribution centered around 0 in INP fractions are not re-identified in LEAP-RBP fractions, supporting their designation as background (red bars x-axis histogram; Supplementary Fig. 14c). Unlike proteins identified exclusively in LEAP-RBP fractions which are found almost entirely within the lower range of detection (red bars y-axis histogram; Supplementary Fig. 14d), many of the background proteins identified exclusively in INP fractions are found within the middle range of detection (red bars y-axis histogram; Supplementary Fig. 14c). This is consistent with observed differences in their abundances (exclusive; Supplementary Fig. 14e). More than half of the proteins identified exclusively in INP fractions are GO-annotated glycoproteins (200/391) which are poorly selected against by repeated AGPC extraction [21]. These background proteins are undetectable in LEAP-RBP fractions by SILAC LC-MS/MS despite acquiring a limit of detection (%TP range) nearly one order of magnitude lower than the INP method (Fig. 6a, b). While the SILAC LC-MS/MS approach accurately identifies background proteins by their $\log_2$(CL/nCL) ratios (~0), non-SILAC comparisons between INP fractions isolated from independent UV-crosslinked and non-crosslinked cellular samples would result in their ubiquitous UV-enrichment (blue boxes; Supplementary Fig. 14f). This would be an example of low method specificity resulting in UV-dependent enrichment of free proteins. Background proteins UV-enriched in this way can be highly variable and sensitive to slight differences in sample processing and method specificity (Supplementary Fig. 15a-c). We attribute the recovery of different background protein profiles to interactions between UV-crosslinking, method-specific RNA-centric enrichment conditions, and protein-specific physicochemical properties (red arrows). These discrepancies during non-SILAC LC-MS/MS experiments may appear as method-specific RNA-binding proteins and hinder meaningful meta-analyses of UV-enriched* proteins identified by different RNA-centric methods.

4e. Signal-dependent recovery of noise is the primary source of free protein for RNA-centric enrichment methods with high specificity for RNA-bound RBPs.

Compared to background proteins ($S = 0$) which can change from observable to undetectable with increasing ($\%TP_S$), unbound RBPs (noise) are only expected to vary in their abundance when recovery of signal is comparable (Fig. 2a, b). Indeed, 93% of RBPs identified in INP fractions are re-identified in LEAP-RBP fractions (813/875) but display higher S/N ratios resulting in higher $\%TP_S$ contributions (89.2 vs 46.1) and lower $\%TP_N$ contributions (7.7 vs 42.8) (Supplementary Fig. 14e). Contrarily, only 39% of non-RBPs identified in INP fractions are re-identified in LEAP-RBP fractions (209/538). While their $\%TP_S$ contributions are roughly 2-fold higher in LEAP-RBP fractions compared to INP fractions (1.6 vs 0.7), a 6-fold decrease in their $\%TP_N$ contributions (1.5 vs 10.5) results in a roughly 4-fold net decrease in their abundance in LEAP-RBP fractions. SILAC LC-MS/MS analysis of LEAP-RBP fractions identified two different populations of free proteins (RBPs and non-RBPs) exhibiting signal-dependent recovery as evidenced by their positive $\log_2$(CL/nCL) ratios (Fig. 4f). Compared to RBPs, non-RBPs displayed lower enrichment efficiencies (S/N ratios) and were less abundant (%TP) (Fig. 6e). This might be due to differences in their physicochemical properties and/or UV-crosslinking efficiencies. For example, many RBPs displaying lower S/N ratios were also found to have lower UV-crosslinking efficiencies (e.g., RPN1; Supplementary Fig. 2a). Nonetheless, the average S/N ratios of non-RBPs (~1) in LEAP-RBP fractions suggests an equivalent amount of free protein

(N) is present and detectable by immunoblot. Indeed, when large quantities of clRNPs were evaluated by SRA, a small amount of RNase-insensitive protein was observed for the cytoskeletal protein β-tubulin identified as UV-enriched* in LEAP-RBP fractions by SILAC LC-MS/MS (Supplementary Fig. 5a). This supports the idea that formation of such complexes is exceedingly rare while demonstrating the critical importance of this orthogonal validation method and SILAC LC-MS/MS to accurately quantify free protein recovery.

The percentage of total protein in the sample that is RNA-bound (%TP_S) can define enrichment limits when repeated utilization of a given enrichment method fails to further increase %TP_S (maximum %TP_S). The ability of a method to achieve maximum %TP_S despite differences in protein UV-crosslinking efficiency (starting %TP_S) and total $|S|$ demonstrates method robustness. For individual RNA-binding proteins, enrichment limits are more appropriately described by the protein-specific metric S/N. In either case, enrichment limits are more readily evaluated when a method is capable of depleting free protein (N) without or signal loss (Supplementary Fig. 16a). Repeated AGPC extraction, INP, LEAP-RBP, and methanol precipitation were demonstrated to recover near 100% of protein-bound RNA (Supplementary Note 3a). Methanol precipitation is a protein-centric method that is unbiased towards RNA-bound (S) and free protein (N) and so it doesn't contribute to %TP_S. INP and LEAP-RBP are RNA-centric enrichment methods which increase %TP_S compared to methanol, but LEAP-RBP achieves higher maximum %TP_S (Fig. 2a, b). Enrichment of clRNPs at the AGPC interphase during repeated AGPC extractions is RNA-centric or protein-centric depending on which interactor is considered responsible for their partitioning at the AGPC interphase. Maximum %TP_S for AGPC extraction typically requires multiple AGPC extractions depending on the %TP_S and total $|S|$ of starting samples. Curiously, the AGPC mixing method affects both the rate of free protein partitioning into the organic phase (Supplementary Fig. 10a), and the maximum %TP_S of the final AGPC interphase (Supplementary Fig. 15a, b). Illustrating these points, the following results were obtained by UV-irradiating cellular samples with 0.4 J/cm^2 (254 nm); RNA and protein UV-crosslinking efficiency is expected to be between 70.0–80.0% and 1.5–2.0% respectively (Supplementary Note 3). Under these UV-crosslinking conditions, maximum %TP_S for INP fractions requires starting from final AGPC interphase suspensions (i.e., maximum %TP_S of repeated AGPC extraction); additional INP steps have minimal impact on %TP_S (Supplementary Fig. 16b). Notably, AGP input suspensions under these UV-crosslinking conditions contain up to ~70x the amount of free protein than the final AGPC interphase suspension. Yet, LEAP-RBP fractions isolated from either suspension display comparable RNase-sensitivity by SRA and Coomassie Blue (protein) staining (Fig. 3a, b); the RNase-sensitivity of total protein in the sample by SRA and Coomassie Blue (protein) staining is considered directly related to the %TP_S of the fraction. This represents a roughly 580-fold enrichment of RNA-bound (total SPI_S) over free (total SPI_N) protein in the starting sample: $(\%TP_S/\%TP_N)_{RNP}/(\%TP_S/\%TP_N)_{input}$; Supplementary Fig. 16c. Most RBPs display comparable RNase-sensitivity (S/N) by SRA and immunoblot in both RNP fractions (Fig. 3b). This indicates that maximum %TP_S for LEAP-RBP fractions does not require starting from final AGPC interphase suspensions. Relating to points noted above, this suggests that recovery of noise for most RBPs is dependent on the amount of RNA-bound protein, not the amount of free protein. This finding was validated by performing a UV-dose response experiment and analyzing LEAP-RBP fractions (with DNA depletion step) by SRA and immunoblot (Supplementary Fig. 6a-c). Assuming 91% of the total protein in LEAP-RBP fractions is RNA-bound in all UV-dose conditions, the fraction isolated from HeLa cells irradiated with 0.1 J/cm^2 represents a roughly 2,500-fold enrichment of RNA-bound protein over free protein in the starting sample. Interestingly, RPN1 displays low S/N in LEAP-RBP fractions isolated from cells irradiated with all UV-doses by SRA and immunoblot unless starting from the final AGPC interphase (Fig. 3b, Supplementary Fig. 6c). Therefore, starting from AGP input suspensions is suitable for most RBPs, but not all. Starting from the final AGPC interphase ensures the maximum %TP_S and sensitivity for LC-MS/MS and SRA analysis regardless of %TP_S and total $|S|$ of input samples. Additional LEAP-RBP steps don't discernibly increase %TP_S when starting from final AGPC interphase suspensions (Supplementary Fig. 11e).

4f. Method robustness and high %TP_S facilitates rigorous assessment of RNA-bound protein abundance.

Like S/N ratios, method robustness and high %TP_S are important for comparative LC-MS/MS studies aimed at identifying differences in RNA-bound protein abundance by limiting the contribution of free protein towards total MS signal (%TP_N) regardless of %TP_S and total $|S|$ of input samples (robustness). This is particularly important for non-SILAC LC-MS/MS experiments where samples are normalized to total SPI.

For example, RNA-bound RBPs in INP and LEAP-RBP fractions display comparable RNA-bound abundance by SRA and immunoblot when they are normalized to µg of protein-bound RNA (Fig. 2b). This approach works because the quantity of RNA-bound protein is considered directly proportional to the µg of protein-bound RNA in the sample regardless of $\%TP_S$ when UV-crosslinking conditions (i.e., total $|S|$ of input samples) and signal recovery are comparable (Supplementary Note 3). However, if INP and LEAP-RBP fractions were normalized to µg of protein (total SPI) for SRA and immunoblot, there would be more visible RNA-bound protein in LEAP-RBP fractions because INP recovers nearly twice as much quantifiable protein (Fig. 2a).

To evaluate how differences in free protein recovery affects accurate assessment of RNA-bound protein abundance ($\%TP_S$), we simulated a label-free LC-MS/MS experiment comparing INP and LEAP-RBP fractions. Because both methods recover near 100% of RNA-bound protein and only differ in the amount of free protein recovered, the absolute quantity of RNA-bound protein in the sample (total $|S|$) is expected to be the same (I vs L; Fig. 2a). For this example, the observed quantities for 357 proteins displaying S/N ratios >3 in both INP and LEAP-RBP fractions by SILAC LC-MS/MS were considered representative of their RNA-bound quantities (>75% RNA-bound). Many of the RBPs which display comparable RNA-bound abundance in INP and LEAP-RBP fractions normalized to signal quantity by SRA and immunoblot fall into this category (e.g., HuR, RPL8, RPL4, TIA1, pAbPC1, PABPC4, XRN1, and LRRC59). The observed quantities (SPI values) for proteins displaying high S/N ratios are predicted to be more comparable between INP and LEAP-RBP fractions when normalizing samples to total RNA-bound protein quantity (total SPI_S) vs total protein quantity (total SPI). To test this prediction, the $\log_2(S/N)$ ratios of proteins identified in LEAP-RBP fractions by SILAC LC-MS/MS were plotted as a function of their relative quantities ($\Delta\log_{10}(SPI)$) as compared to INP fractions. As expected, the relative quantities of proteins dispaying S/N ratios >3 in both fractions appear increased when samples are normalized to total protein in the sample (total SPI) (Supplementary Fig. 16d), but comparable when samples are normalized to total RNA-bound protein in the sample (total SPI_S) (Supplementary Fig. 16e, Supplementary Methods). The relative quantities of proteins when samples are normalized to total SPI is equivalent to their relative abundances ($\Delta\log_{10}(SPI) = \Delta\log_{10}(\%TP)$). As such, the observed increase in relative quantities of proteins displaying S/N ratios >3 may be interpreted as an increase in RNA-bound abundance ($\%TP \approx \%TP_S$ and $\Delta\log_{10}(\%TP) \approx \Delta\log_{10}(\%TP_S)$). These data demonstrate how differences in free protein recovery, emulated here by comparing methods with varying $\%TP_S$ and comparable signal recovery, can appear as proteome-wide changes in RNA-bound protein abundances ($\%TP_S$).

By SILAC LC-MS/MS, an estimated 91% of the total protein in LEAP-RBP fractions is RNA-bound (total SPI $\approx$ total SPI_S). Therefore, observed abundances for proteins displaying S/N ratios >3 ($\%TP \approx \%TP_S$), can be considered representative of their RNA-bound abundances estimated as a percentage of total RNA-bound protein in the sample ($\%TP \approx \%TP_S \approx \%TP_{(S)}$). An increase in the observed abundance (%TP) of RBPs relative to non-RBPs with increasing $\%TP_S$ reflects decreased contributions from their unbound counterparts (i.e., $\%TP_N$). Because LEAP-RBP recovers near 100% of total RNA-bound protein—or herein total cellular RNA-bound protein—observed %TP for proteins displaying S/N ratios >3 is considered representative of their total cellular RNA-bound abundances; notably, total RNA-bound protein abundance = $\%TP_{(S)}$ only when total RNA-bound protein "in the sample" is considered representative of total RNA-bound protein (Supplementary Methods).

The high $\%TP_S$ (>90%) of LEAP-RBP fractions makes it possible to perform label-free comparative LC-MS/MS experiments aimed at identifying differences in RNA-bound protein abundance (Fig. 7b-h). For this, we suggest normalizing samples to total SPI as in the previous example. Most label-free quantification (LFQ) algorithms, such as the MaxQuant LFQ algorithm MaxLFQ, assumes samples are relatively similar and do not include weighting factors that prioritize protein abundance [22]. This is problematic, as LEAP-RBP fractions contain many low-abundance proteins with significant free protein contributions (i.e., low S/N). Because they only contribute 3% of total SPI, weighting them equally contributes additional sources variance that is unlikely to reflect the biological activity of interest (i.e., direct RNA-binding). Conversely, the most abundant proteins in LEAP-RBP fractions display high S/N and appear well-conserved (RNase, clRNP fractions; Supplementary Fig. 8b). Therefore, they should be given more weight when normalizing samples for comparative LC-MS/MS studies. While it has been reported that LFQ algorithms can be successfully

applied to samples following protein enrichment steps (e.g., co-immunoprecipitation), it was also noted that these studies benefit from a dominant and reproducible population of background proteins exhibiting minimal changes between experimental conditions [22]. In contrast, RNA-centric enrichment of RNA-bound protein is arguably more of an indirect form of protein enrichment. As noted previously, recovery of free protein during RNA-centric enrichment of RNA-bound protein is enigmatic. The potential interactions between the UV-crosslinking, RNA-enrichment conditions, and protein-specific physicochemical properties currently cannot be predicted with certainty (Supplementary Fig. 15a-c, Supplementary Note 4c-e). Recognizing this limitation, normalizing to total SPI makes the least assumptions about sample identities and leverages the high %TP_S and robustness of the LEAP-RBP method.

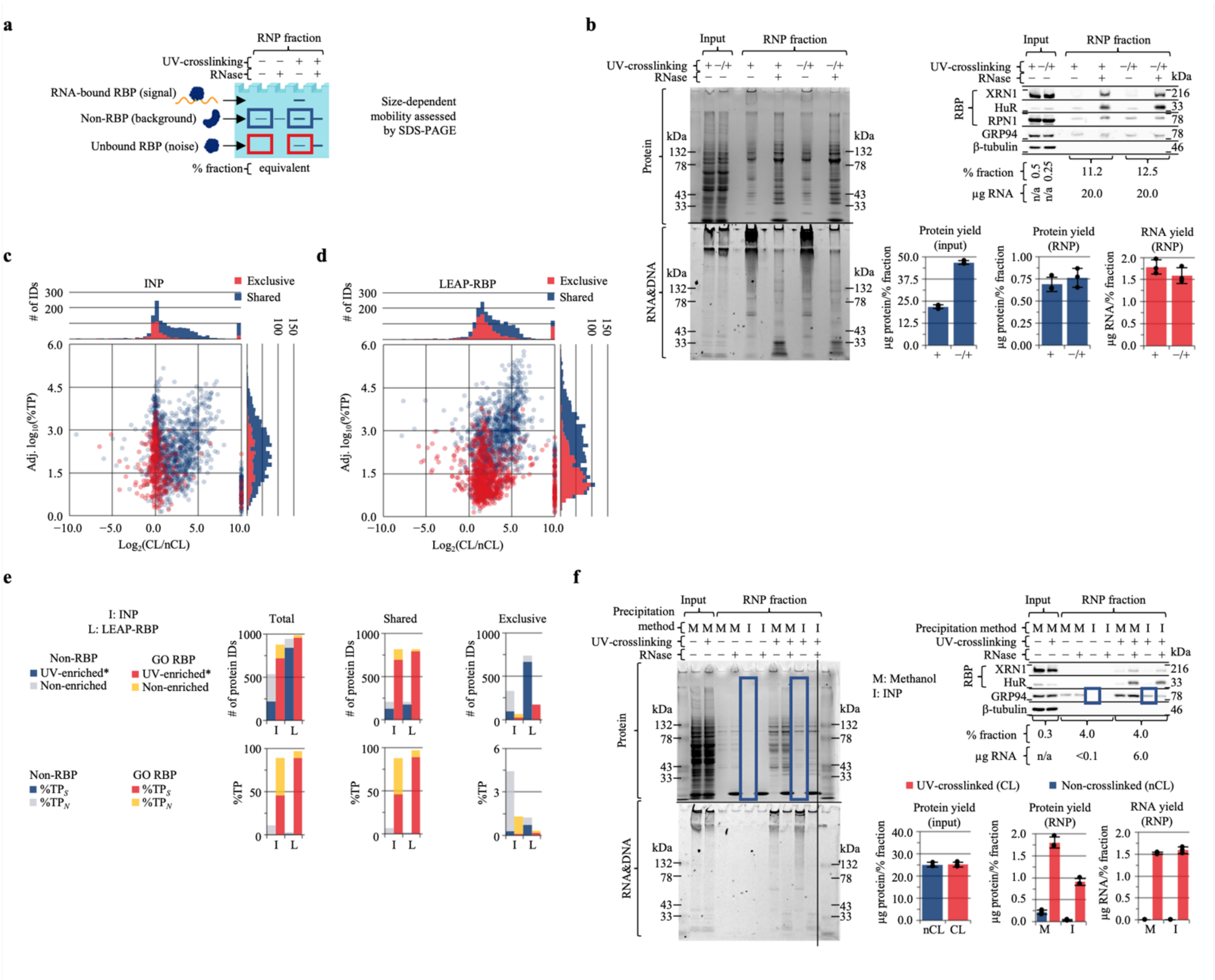

Supplementary Figure 14: UV-dependent enrichment of free protein vs signal-dependent recovery of noise. AGPC: acidic guanidinium thiocyanate-phenol-chloroform; AGP: acidic guanidinium thiocyanate-phenol; SRA: SRA analysis. **a** Graphical representation illustrating the predicted behavior of free protein exhibiting UV-dependent enrichment or unbound RBPs exhibiting signal-dependent recovery during SRA analysis. Graphic prepared in BioRender. **b** Comparison of INP fractions isolated from final AGPC interphase suspensions of cellular samples containing 10 million UV-crosslinked (denoted by +) or 10 million UV-crosslinked and non-crosslinked (denoted by −/+) HeLa cells by SRA and Coomassie Blue (protein), SYBR Safe (RNA&DNA) staining, or western blot. RNA (UV-spectrophotometry) and protein (BCA) quantitation data shown in adjoining bar charts represent the mean ± 1 *SD* of 3 biologically independent samples, pooled (equivalent % fraction) for SRA. Sample normalization values indicated in the figure panel. **c**, **d** Scatterplots illustrating the difference in protein abundance ($\log_{10}$(%TP)) and UV-enrichment ($\log_2$(CL/nCL)) of proteins identified exclusively in INP (**c**) or LEAP-RBP (**d**) fractions by SILAC LC-MS/MS (Fig. 4a). **e** Stacked bar charts showing the number of RBPs and non-RBPs identified as UV-enriched* or non-enriched by each method, or the estimated $\%TP_S$ and $\%TP_N$ contributions of RBPs and non-RBPs in each RNP fraction. Additional stacked bar charts show the contributions of proteins identified by INP and LEAP-RBP methods (shared), or those identified exclusively by INP or LEAP-RBP methods (exclusive). **f** Comparison of methanol (95% v/v) precipitated AGPC interphase samples and INP fractions isolated from final AGPC interphase suspensions of 10 million UV-crosslinked (0.4 J/cm^2, 254 nm) or non-crosslinked HeLa cells by SRA and Coomassie Blue (protein), SYBR Safe (RNA&DNA) staining or western blot. Protein (BCA) and RNA (UV-spectrophotometry) quantitation data shown in the adjoining bar charts represent the mean ± 1 *SD* of 3 biologically independent samples, pooled (equivalent % fraction) for SRA. Sample normalization values indicated in the figure panel. Experiments were performed twice with similar results (**b**, **d**). Source data are provided as a Source Data file.

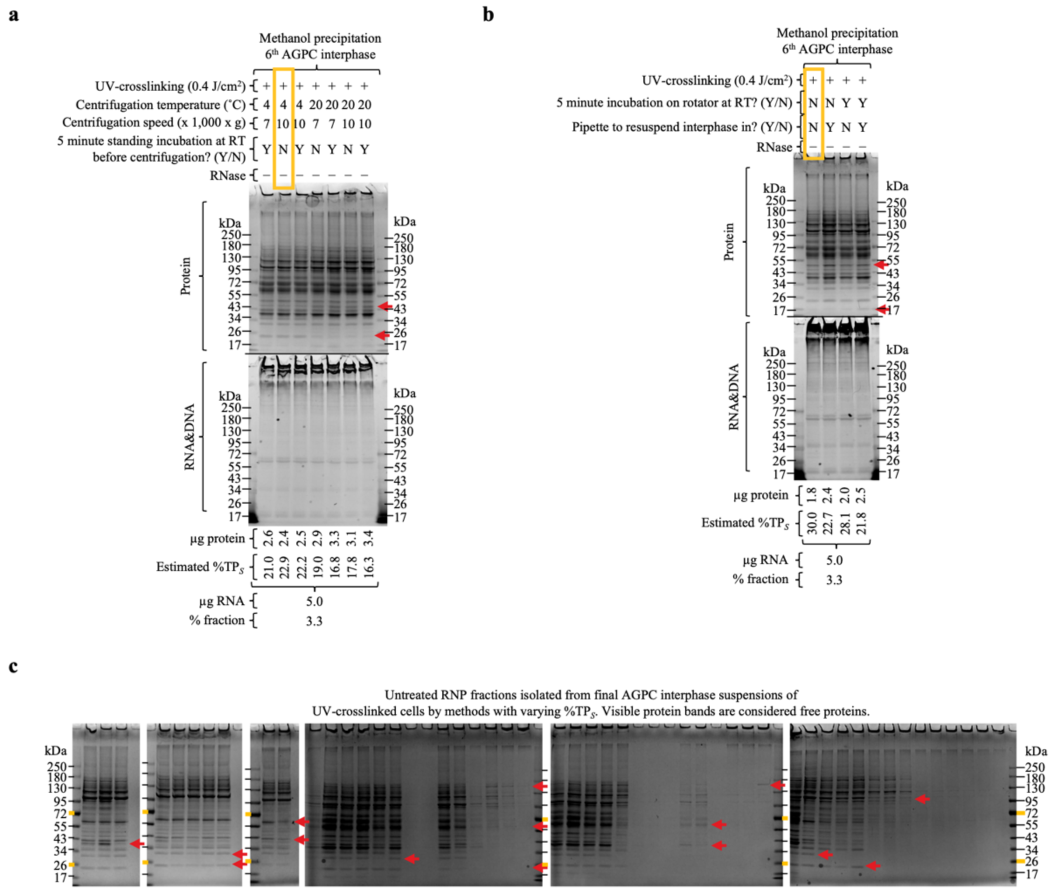

Supplementary Figure 15: Recovery of background proteins during RNA-centric enrichment of clRNPs is dependent on RNA enrichment conditions. AGPC: acidic guanidinium thiocyanate-phenol-chloroform; AGP: acidic guanidinium thiocyanate-phenol. **a**, **b** Comparison of untreated AGPC interphase samples isolated from the final AGPC interphase or final AGPC interphase suspension of 10 million UV-crosslinked (0.4 J/cm^2, 254 nm) HeLa cells by SDS-PAGE and Coomassie Blue (protein), SYBR Safe (RNA&DNA) staining. Protein (BCA) and RNA (UV-spectrophotometry) quantitation data represents mean of 1 independent sample, normalized to µg RNA for SDS-PAGE. Red arrows point to free proteins exhibiting AGPC extraction condition-dependent recovery. **c** Comparison of untreated RNP fractions isolated from final AGPC interphase suspensions of 10 million UV-crosslinked (0.4 J/cm^2, 254 nm) HeLa cells using different RNA enrichment conditions by SDS-PAGE and Coomassie Blue (protein) staining. Samples were normalized to % fraction for SDS-PAGE, n = 1 independent sample. Red arrows point to free protein exhibiting RNA-centric enrichment condition-dependent recovery. Experiments were performed once (**a**-**c**). Source data are provided as a Source Data file.

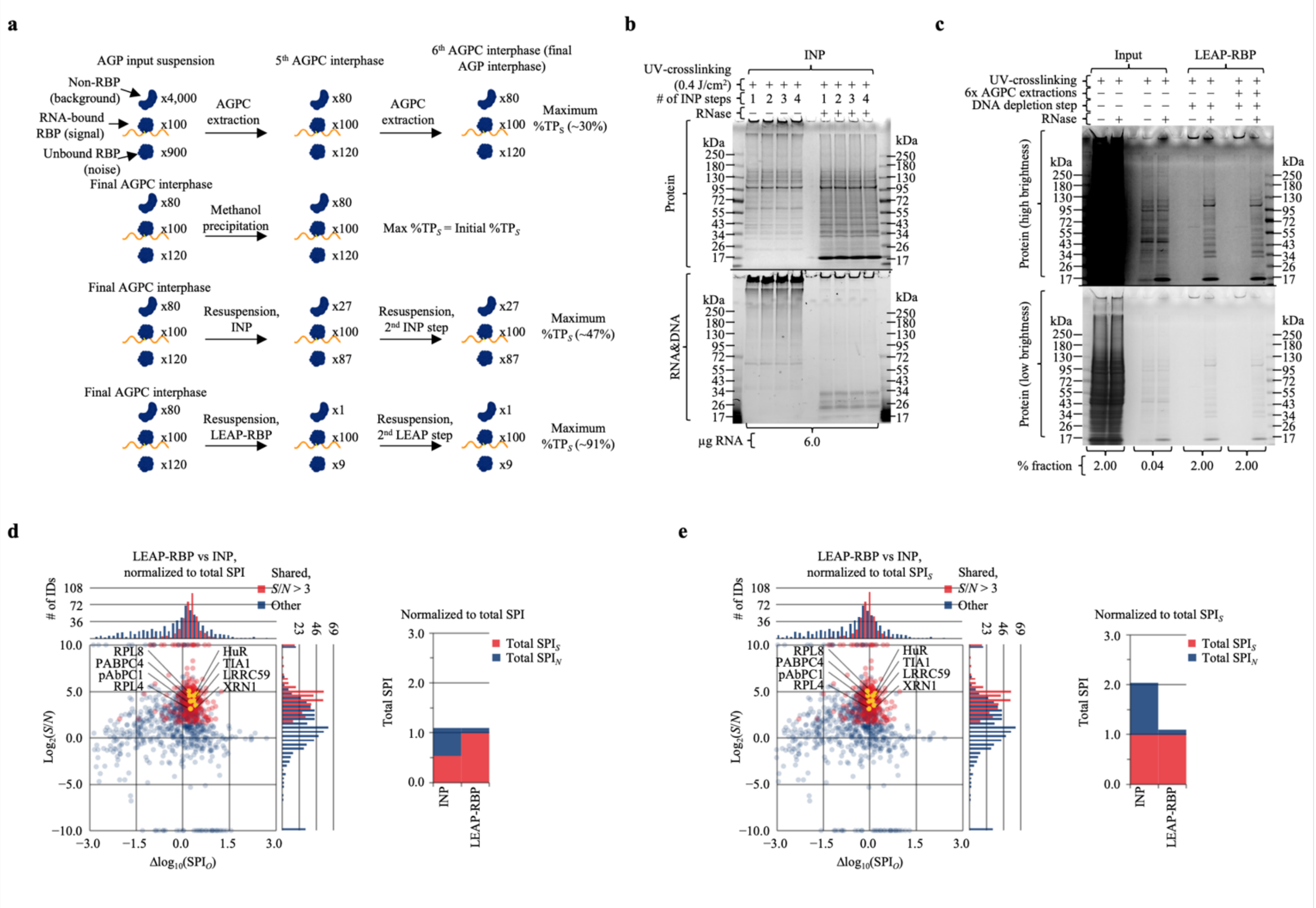

Supplementary Figure 16: Method robustness and high $\%TP_S$ facilitates rigorous assessment of RNA-bound protein abundance. AGPC: acidic guanidinium thiocyanate-phenol-chloroform; AGP: acidic guanidinium thiocyanate-phenol; SRA: SRA analysis. **a** Graphical representation illustrating how $\%TP_S$ can be used to define enrichment limits when repeated utilization fails to further increase the percentage of total protein isolated that is RNA-bound ("maximum $\%TP_S$"). The maximum $\%TP_S$ of INP and LEAP-RBP fractions were estimated by SILAC LC-MS/MS (Fig. 5h). The maximum $\%TP_S$ of the final AGPC interphase was estimated using the relative amount of protein recovered from the final AGPC interphase by methanol (95% v/v) precipitation as compared to LEAP-RBP (Fig. 2a). Graphic prepared in BioRender. **b** Comparison of RNP fractions isolated from final AGPC interphase suspension of 10 million UV-crosslinked (0.4 J/cm^2, 254 nm) HeLa cells using 1–4 repeated INP steps by SRA with Coomassie Blue (protein), SYBR Safe (RNA&DNA) staining. RNA (UV-spectrophotometry) quantitation data represents mean of 1 independent sample. Samples were normalized to µg RNA for SDS-PAGE. **c** Comparison of LEAP-RBP fractions isolated from AGP input or final AGPC interphase suspensions (CL), and input samples (total protein) by SRA and Coomassie Blue (protein) stain; n = 3 biologically independent samples, pooled (equivalent % fraction) for SRA. **d**, **e** Scatterplots illustrating how the observed quantities ($\log_{10}(SPI_O)$) of proteins considered representative of their RNA-bound quantity in both LEAP-RBP and INP fractions ($S/N > 3$) appear increased in LEAP-RBP fractions when samples are normalized to total SPI (**d**), but comparable when samples are normalized to total SPI_S (**e**). Corresponding bar charts show the effect of sample normalization method on relative amount of total RNA-bound (total SPI_S) and free (total SPI_N) protein in the sample. Experiments were performed once (**b**), or four times with similar results (**c**). Source data are provided as a Source Data file.

Supplementary Note 5
This note provides supporting information and rationale for protein-specific *S*/*N* ratios, SILAC LC-MS/MS, and MS data handling.

5a. Rationale behind protein *S*/*N* ratios.
In this study, *S*/*N* of proteins represents the ratio of RNA-bound to unbound counterparts, while proteins without RNA-bound counterparts represent background proteins ($S = 0$) (Supplementary Fig. 17a). These designations were chosen because LC-MS/MS analysis will not differentiate whether a tryptic peptide originated from RNA-bound or unbound counterparts (red box; Supplementary Fig. 17a). Notably, peptide UV-crosslinked to RNA moieties can be distinguished [5, 21], but most tryptic peptides are not expected to be directly crosslinked to RNA. Conceptually, *S*/*N* of proteins is similar to the *S*/*N* of MS-peak intensities. Therefore, comparisons between the two helps illustrate the scientific rationale behind *S*/*N* ratios as a protein-specific metric herein.

In a typical LC-MS/MS experiment, peptides derived from proteolytic digestion generate peak ion intensities over their expected time window (arrows; Supplementary Fig. 17b) [23]. All mass spectrometers detect a background signal in the absence of peptides which fluctuates over time (blue bracket; Supplementary Fig. 17b). The background signal is indistinguishable from the signal generated by ionized peptides during their expected time window. Therefore, accurate quantification of peptide intensities (*S*) requires estimating the contributions of background noise (*N*) to the total peak ion intensities ($S + N$). To do this, background noise (*N*) is estimated "off-peak" as the distance between peak background signal and the average background noise $(X\text{-bar})_B$ (blue bar; Supplementary Fig. 17c). Integrated peptide intensities (*S*) are generated by subtracting the integrated noise intensities (*N*) from the integrated peak ion intensity ($S + N$) over the same time window (Supplementary Fig. 17d). The *S*/*N* ratio can be estimated by dividing the integrated peptide intensities by the integrated noise intensities [9]. When $S = 0$, $N - N = 0$ (background) and *S*/*N* is undefined.

The same basic *S*/*N* principles were used herein to represent the ratio of RNA-bound to unbound counterparts. Necessarily, this represents an additional noise contribution above the background noise of the mass spectrometer (Supplementary Fig. 17e). Quantified peptide intensities of protein (*O*) represent the sum of peptide intensities from RNA-bound (*S*) and unbound (*N*) counterparts. However, the contributions from unbound protein cannot be estimated "off-peak" because the peptides from RNA-bound counterparts have the same retention time and will map to the same protein. Importantly, estimating noise contributions in UV-crosslinked samples by performing LC-MS/MS on independent non-crosslinked samples will underestimate the amount of free protein (Supplementary Note 4d). Therefore, noise is more accurately estimated using a SILAC-based approach as shown in Supplementary Fig. 17f, where SILAC-labeled UV-crosslinked (CL, red) and non-crosslinked (nCL, blue) samples are pooled prior to RNA-centric enrichment. Because (red) peptides from UV-crosslinked cells will have longer retention times than (blue) peptides from non-crosslinked cells, they can now be independently quantified (Supplementary Fig. 17g). The peptide intensities observed in the non-crosslinked SILAC channel provide an "off-peak" equivalent to background noise from the previous example by assuming equal noise-partitioning between SILAC channels (Supplementary Fig. 17h, Supplementary Note 6a).

5b. Strategies for estimating noise contributions.
Estimating background noise contributions of the mass spectrometer benefits from a large sampling size (# of background signal peaks; Supplementary Fig. 17b). However, estimating noise contributions for individual peptides in the UV-crosslinked SILAC channels based on a single peak from the non-crosslinked SILAC channel would introduce additional variance or fail when peptides in the non-crosslinked SILAC channel are undetected above the background instrument noise. Supplementary Fig. 18a details different strategies for data sets with missing peptide intensities in the non-crosslinked SILAC channel, starting from quantified peptide intensities (red box). The concerning issue of missing peptide in the non-crosslinked SILAC channel has been previously noted. For example, the authors of the TRAPP (purple arrows & boxes) and XRNAX (blue arrows & boxes) methods used a comparable SILAC-based approach for identification of UV-enriched* proteins and provide solutions for absent peptides in non-crosslinked samples [4, 5]. In one approach, missing peptide data is imputed computationally, via random selection of a peptide intensity value from the bottom

percentile of all peptide intensities. This approach rarely introduces additional variance if sum peptide intensities (SPI) are used for calculating log(CL/nCL) ratios because the SPI values mainly reflect the more abundant peptides identified across all samples and in both SILAC channels (purple vs gold boxed SPI bar charts). However, if UV-enriched* proteins are identified by calculating the log(CL/nCL) ratio of each peptide, as done for XRNAX, imputing values can introduce unmeaningful variance. This strategy is used for experiments where replicates are limited (n = 1–2) and treating peptides as independent observations enables hypothesis testing [5, 8, 13, 24]. While peptide ratios are generally more variable, the larger sampling size (# of peptides) compensates by increasing statistical power (SEM). Nonetheless, the additional variance from log(CL/nCL) ratios calculated using imputed peptide values is unmeaningful [5]. Noting this, the authors of XRNAX filter for peptides only detected in the UV-crosslinked SILAC channel and use the same pseudo-count as the denominator for all "super-enriched" peptides ([5]; Fig. S1G). This approach is equivalent to subtracting the identical background noise from all peak intensities so that only peptide intensities in the UV-crosslinked SILAC channel contributes variance.

Both strategies have merit and for different reasons. The strategy used by the authors of the TRAPP method avoids underestimating the amount of free protein in the sample (%TP_N), and the strategy used by the authors of the XRNAX method avoids unmeaningful variance introduced by free proteins observed in the non-crosslinked SILAC channel. In the current study, protein quantities in each SILAC channel were estimated as the sum of their identified peptide intensities without imputing missing peptide intensities (gold arrows & boxes; Supplementary Fig. 18a). Estimating relative protein quantities using sum peptide intensities does not require that the same number of peptides are quantified in each sample [25]; SPI values mostly reflect the most abundant peptides identified across all samples and in both SILAC channels. After normalizing samples to total SPI, SPI_{nCL} values equal to 0 were replaced with the average non-zero SPI_{nCL} value (Supplementary Methods). Log_2(CL/nCL) and $log_2(S/N)$ ratios were generated using SPI_{CL} values and average SPI_{nCL} values according to equations (2) and (3) respectively. This analytical approach has the benefit of avoiding unmeaningful variance introduced by free proteins observed in the non-crosslinked SILAC channel as shown in Supplementary Fig. 19a, b, Supplementary Note 6b.

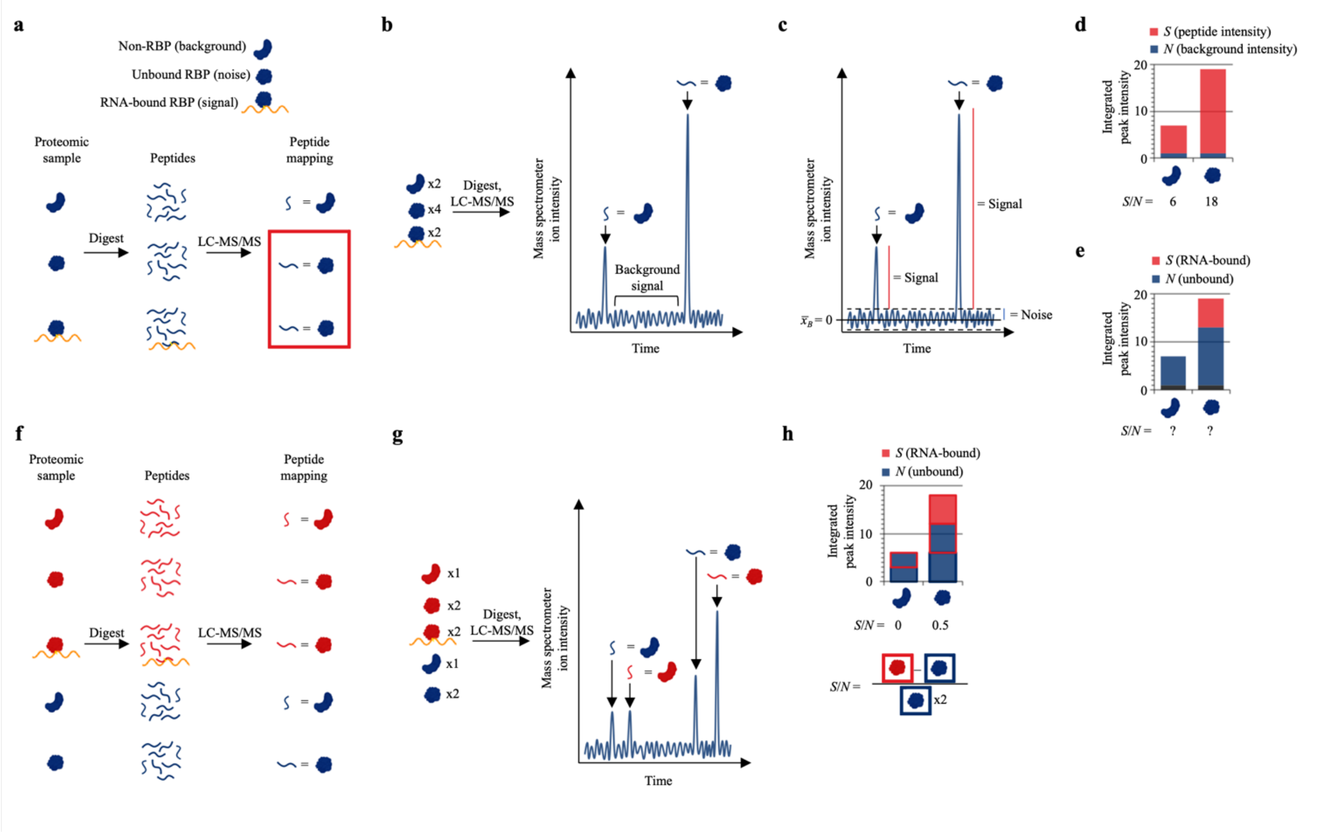

Supplementary Figure 17: Estimating noise contributions during LC-MS/MS analysis requires a SILAC-based approach. a Graphical representation illustrating how most tryptic peptides from unbound RBPs (noise) will map to the same protein as those from RNA-bound counterparts (signal) during LC-MS/MS. **b** Graphical representation showing the expected retention time of the tryptic peptides shown in panel **a** (red box) originating from RNA-bound RBPs (signal), their unbound counterparts (noise), and non-RBPs (background) during LC-MS/MS analysis. **c** Same chromatograph from panel **a** with red bars representing peptide intensities (signal) and a blue bar representing background intensities (noise). **d** Stacked bar chart showing the expected contributions of integrated peptide intensities (*S*) and background intensities (*N*) towards the observed MS-peak intensities (*O*); *S*/*N* can be calculated by dividing *S* by *N*. **e** Stacked bar chart showing the expected contributions of integrated peptide intensities from RNA-bound (*S*) and unbound (*N*) protein towards the observed integrated peptide intensities (*S* + *N*); *S*/*N* can't be calculated without being able to estimate noise contributions "off-peak". **f** Graphical representation illustrating how tryptic peptides originating from unbound RBPs (*N*) in the non-crosslinked SILAC channel (blue peptides) will be distinguished from tryptic peptides originating from both RNA bound and unbound RBPs (*S* + *N*) in the UV-crosslinked channel (red peptides) during LC-MS/MS. **g** Graphical representation showing the difference in retention time of tryptic peptides shown in panel **f** originating from UV-crosslinked (red peptides) or non-crosslinked samples (blue peptides). **h** Stacked bar chart showing the expected contributions of integrated peptide intensities from RNA-bound (*S*) and unbound (*N*) protein towards the observed integrated peptide intensities (*S* + *N*) across both SILAC-channels (red and blue peptides). *S*/*N* can be calculated using integrated peptide intensities observed in the non-crosslinked SILAC channel (blue peptides) to estimate noise contributions towards the observed integrated peptide intensities in the UV-crosslinked SILAC channel (red peptides). Notably, this assumes equal noise-partitioning between SILAC channels. Graphics (**a**-**h**) were prepared in BioRender. Source data are provided as a Source Data file.

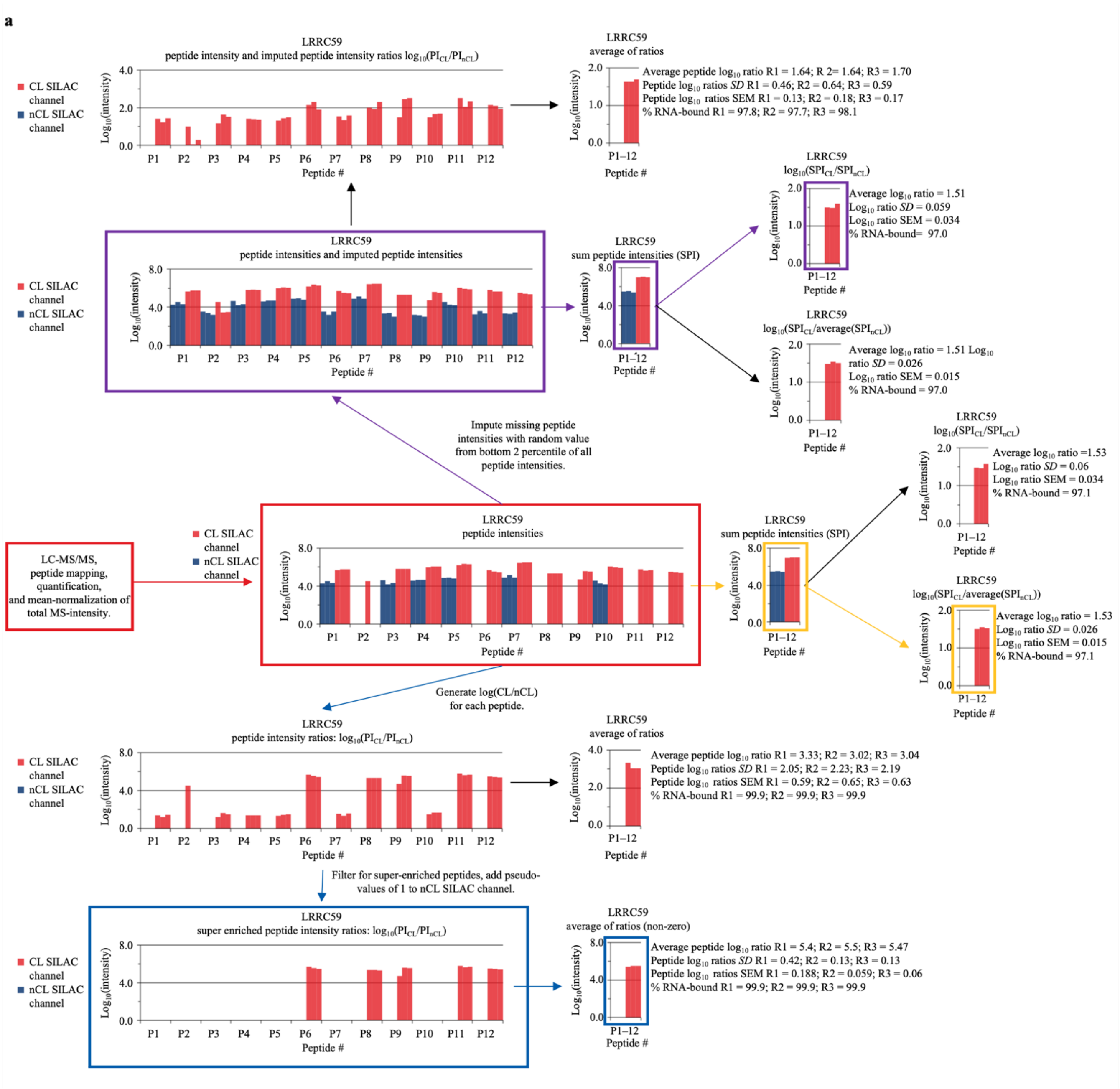

Supplementary Figure 18: Alternative strategies for dealing with missing peptide intensities from non-crosslinked samples. a Bar charts show the $\log_{10}$ transformed integrated peptide intensities for peptides that were mapped to LRRC59 during SILAC LC-MS/MS analysis of LEAP-RBP fractions (*n* = 3 biologically independent samples). In this study, the sum of individual peptide intensities (red box) or sum peptide intensities (SPI) were used to calculate log(CL/nCL) ratios using the average SPI_{nCL} values (gold arrows and boxes). In the referenced TRAPP study [4], missing peptide intensities were imputed prior to generation of sum peptide intensities (SPI) and calculation of log(CL/nCL) ratios (purple arrows and boxes). In the referenced XRNAX study [5], super-enriched peptides (PI_{nCL} = 0) were used to calculate the average of peptide log(CL/nCL) ratios by setting PI_{nCL} values to 1 (blue arrows and boxes). Source data are provided as a Source Data file.

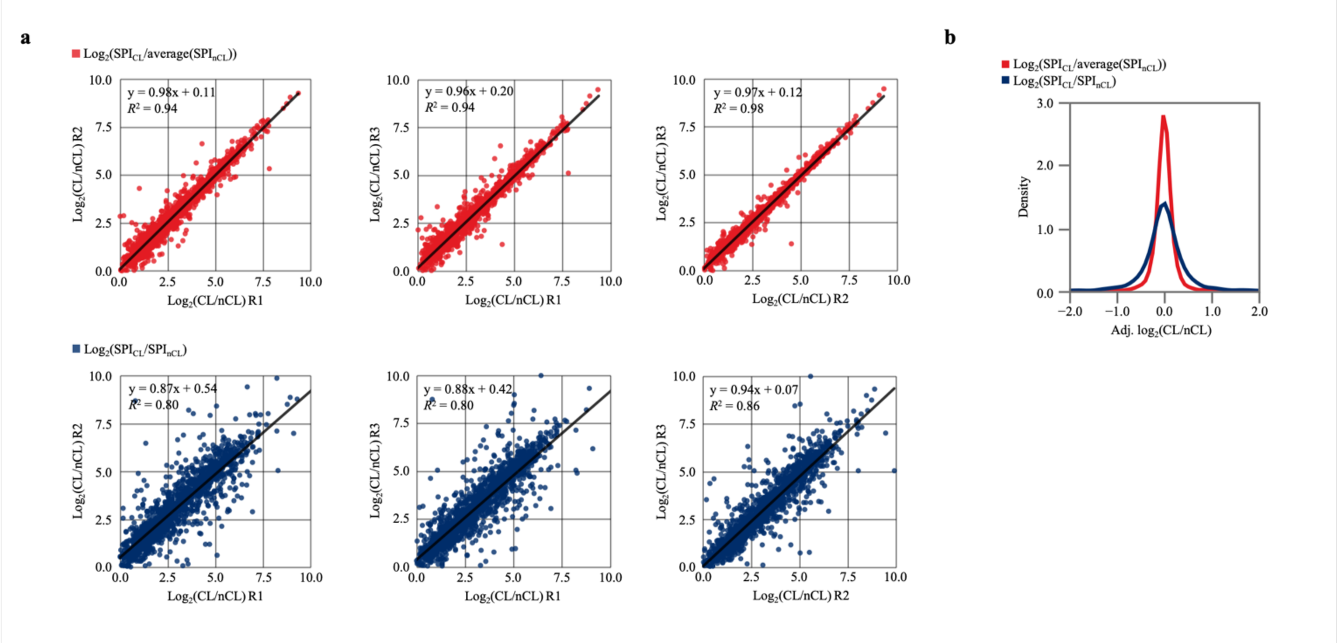

Supplementary Figure 19: Alternative strategies for calculating $\log_2$(CL/nCL) ratios. Analyses were performed with MS data generated by SILAC LC-MS/MS analysis of LEAP-RBP fractions (Supplementary Data 3, Methods). **a** Scatterplots comparing $\log_2$(CL/nCL) ratios between replicate samples using the average SPI_{nCL} value (red) or SPI_{nCL} value of each replicate (blue). **b** Probability density curves comparing the standard deviation of $\log_2$(CL/nCL) ratios generated using the alternative strategies shown in **a**. Source data are provided as a Source Data file.

Supplementary Note 6
This note provides supporting information for *S*/*N*-based analyses and additional considerations for comparative LEAP-RBP experiments and other downstream applications.

6a. Accurate evaluation of *S*/*N* by LC-MS/MS analysis requires a SILAC-based approach.
If only RNA-bound proteins exhibit UV-dependent enrichment, then *S*/*N* can be readily quantified by label-free LC-MS/MS analysis of independent UV-crosslinked and non-crosslinked samples (Supplementary Fig. 20a). However, UV-dependent enrichment of free protein is a widely observed phenomenon (Supplementary Note 4b, c). As an example, performing repeated AGPC extraction and LEAP-RBP on independent UV-crosslinked and non-crosslinked samples only yields detectable protein from UV-crosslinked samples (Supplementary Fig. 5a). If the assumption that only RNA-bound proteins exhibit UV-dependent enrichment is true, then all the isolated protein is RNA-bound. However, when differentially SILAC-labeled non-crosslinked and UV-crosslinked cells are pooled prior to isolation of the clRNP fraction and analyzed by LC-MS/MS, many peptides originating from non-crosslinked cells were observed (Fig. 4a). As these peptides do not originate from UV-crosslinked RNA-bound protein, the assumption that only RNA-bound proteins exhibit UV-dependent enrichment is false. Because the recovery of free protein in LEAP-RBP fractions is dependent on signal quantity and is independent of total protein and RNA (Fig. 3a, b, Supplementary Fig. 6a-c), *S*/*N* ratios were calculated assuming equal noise-partitioning between SILAC channels (Supplementary Fig. 20b).

The analyses presented in this study considered protein quantities observed in both SILAC channels. However, if $SPI_{\text{-CL}}$ values were used to estimate free protein contributions in the UV-crosslinked SILAC channel, but ignored during comparative analyses, this would effectively increase the $\log_2(S/N)$ ratio of all proteins by 1 ($\log_2(S/N_{(\text{CL})}) = \log_2(S/N) + 1$); Supplementary Fig. 20c). Additionally, if protein abundances were estimated as a percentage of total protein in the UV-crosslinked SILAC channel (total SPI_{CL}), this would effectively increase the observed abundance of RNA-bound proteins by halving free protein contributions ($\%TP_{(\text{CL}),\, S} = \%TP_S + \%TP_N/2$). Because the $\%TP_{(S)}$ contributions of RBPs (98.3) are higher than their $\%TP_{(N)}$ contributions (83.4), and $\%TP_{(S)}$ contributions of non-RBPs (1.7) are lower than their $\%TP_{(N)}$ contributions (16.6), the observed abundance of RBPs relative to non-RBPs will increase (Source Data Fig. 5h).

$$\%TP_{(S),\,\text{RBPs}}/\%TP_{(S),\,\text{non-RBPs}} > \%TP_{(\text{CL}),\,\text{RBPs}}/\%TP_{(\text{CL}),\,\text{non-RBPs}} > \%TP_{\text{RBPs}}/\%TP_{\text{non-RBPs}}$$

Indeed, performing this type of data handling results in the expected transformations (Supplementary Fig. 20d-f). We propose this as an analytical strategy for comparative LEAP-RBP experiments utilizing a SILAC-labeling approach to further enhance *S*/*N*. However, normalizing samples to total SPI_{CL} as compared to total SPI is not expected to provide significant benefits (Supplementary Note 6b).

6b. *S*/*N* ratios serve as a key metric for identifying $\Delta\log_2(S)$ and avoiding $\Delta\log_2(N)$.
From an *S*/*N* perspective, UV-enrichment* indicates there is more protein recovered from UV-crosslinked ($S + N$) than non-crosslinked samples (N). This is comparable to testing whether a given MS peak intensity ($S + N$) can be distinguished from background noise (N) of the mass spectrometer (Supplementary Note 5a); and is often referred to as the "limit of detection" or LOD [26]. However, the point at which a change in peptide intensity (S) can be reliably detected is much higher and often called the "limit of quantification" or LOQ [27, 28]; here, *S*/*N* describes the relative contributions of peptide intensity (S) and background noise (N) towards the observed MS peak intensity (O). Because they have different sources of variance, the *S*/*N* ratio also describes their relative contributions towards the observed variance. Similarly, the *S*/*N* ratio of proteins describes the relative contributions of their RNA-bound (SPI_S or S) and unbound counterparts (SPI_N or N) towards their observed quantities (SPI_O, SPI, or $S + N$). $\log_2(S/N)$ ratios therefore provide a means to evaluate their contributions in a way that $\log_2(\text{CL/nCL})$ ratios can't by providing a total function (Supplementary Fig. 21a, b). Theoretically, proteins with $\log_2(\text{CL/nCL})$ ratios less than 0 cannot contain signal, just as MS peak intensities ($S + N$) below background noise (N) cannot contain peptide intensities (S). At a $\log_2(S/N)$ ratio of 0, RNA-bound and unbound counterparts contribute equally to the observed quantity and variance of proteins (Supplementary Fig. 21c, d). In this study, we emphasize the importance of having sufficient *S*/*N* to detect a change in $\log_2(S + N)$ in response to $\Delta\log_2(S)$ (Fig. 5d, e). Conversely, having sufficient *S*/*N* is important for avoiding a change in $\log_2(S + N)$ in response to $\Delta\log_2(N)$ (Supplementary Fig. 21e). Therefore, *S*/*N* is a key

metric for statistical analysis during comparative experiments because it helps identify differences in protein recovery more likely to reflect $\Delta\log_2(S)$.

The relationships shown in Supplementary Fig. 21e assume RNA-bound and unbound counterparts have similar variability. To test this assumption, $\log_2(SPI_{nCL})$ and $\log_2(SPI_{CL})$ values generated during SILAC LC-MS/MS analysis of LEAP-RBP fractions (n = 3) were used to assess the variability of $\log_2(N)$ and $\log_2(S)$ values respectively. For LEAP-RBP fractions, the variability of $\log_2(SPI_{CL})$ values provide a good approximation for the variability of $\log_2(S)$ values because an estimated 95% of the total protein observed in the UV-crosslinked SILAC-channel is RNA-bound ($\%TP_{(CL), S}$ = 95; Supplementary Fig. 20f, Supplementary Note 6a). Only proteins detected in all three LEAP-RBP fractions and across both SILAC-channels were included (n = 1743, ~90% of protein IDs). SPI values were $\log_2$ normalized and adjusted by subtracting the mean $\log_2$ normalized value of all three replicates for each protein ID. Values for each replicate were treated as independent observations (n = 5229). As expected, the probability density distribution of $\log_2(SPI_{nCL})$ values is wider (SD = ~0.5) than the probability density distribution of $\log_2(SPI_{CL})$ values (SD = ~0.3) regardless of the normalization method used (Supplementary Fig. 21f). Furthermore, the density distribution of $\log_2(SPI_{nCL} + SPI_{CL})$ values is more comparable to the density distribution of $\log_2(SPI_{CL})$ values. This reflects the larger contribution of UV-crosslinked samples towards total SPI ($SPI_{nCL} + SPI_{CL}$), and the larger contribution of RNA-bound protein towards the observed variance of $\log_2(SPI_{CL})$ values. Indeed, Levene's test for equality of variances did not detect a significant difference in variance between $\log_2(SPI_{nCL} + SPI_{CL})$ and $\log_2(SPI_{CL})$ values: $F(1, 10456) = 0.54, p = .464$, but there was a significant difference in variance between $\log_2(SPI_{CL})$ and $\log_2(SPI_{nCL})$ values $F(1, 10456) = 748.96, p < .001$. Based on these data, normalizing samples to total SPI_{CL} as compared to total SPI during SILAC LC-MS/MS experiments is not expected to provide significant benefits. Additionally, the variability of observed quantities $\log_2(S + N)$ is expected to increase with decreasing S/N (Supplementary Fig. 21g).

6c. Setting *S/N* limits for comparative LEAP-RBP experiments.

RNA-bound proteins and their unbound counterparts have different physicochemical properties and sources of variance. While UV-crosslinking conditions are the main source of variance for RNA-bound proteins (Supplementary Note 3), the sources of variance for their unbound counterparts are enigmatic (Supplementary Note 4). For example, more unbound RPL4 is recovered by LEAP-RBP from HeLa cells than the other three cell lines examined without a discernible difference in RNA-bound abundance (untreated (N) vs RNase ($S + N$), clRNP fraction; Fig. 7j). Furthermore, less unbound TIA-1a (upper band) is recovered from 832/13 cells than the other three cell lines without a discernible difference in RNA-bound abundance (Fig. 7j). Because both RPL4 and TIA-1 display high S/N in LEAP-RBP fractions, however, differences in noise recovery is less likely to obscure changes in RNA-bound quantity. By setting rigorous criteria for comparative experiments, signal-dependent recovery of noise can be reconciled. In this study, S/N ratios of proteins were estimated by SILAC LC-MS/MS analysis of LEAP-RBP fractions; however, S/N ratios can also be estimated by SRA and immunoblot (red boxes; Supplementary Fig. 1a). The limit of quantification (LOQ) for MS-peak intensities represents the acceptable S/N for detecting a change in peptide intensity (S). For reliable measurement of analyte concentration, a S/N ratio of 10 or higher is recommended as the LOQ [27]. However, a S/N between 3–10 has been proposed as a more practical quantification limit for simply detecting a significant difference in analyte concentration when comparing experimental and control samples [28]. For RBPs, an S/N ratio of 3–10 indicates 75–91% of observed protein quantities are RNA-bound (Source Data Fig. 5c, Supplementary Note 6b). Based on SILAC LC-MS/MS analysis of LEAP-RBP fractions, 911 proteins display S/N ratios >3 (Supplementary Data 3); 770 of these were re-identified during the comparative LEAP-RBP experiment utilizing a label-free LC-MS/MS approach (Fig. 7b-h, Supplementary Data 9).

6d. Application and utility of LEAP-RBP.

Because of its high selectivity for RNA-bound species, LEAP-RBP is a valuable tool for RBP studies. Principally, LEAP-RBP and the methods herein allow quantitative recovery of total protein, RNA, and clRNPs and estimation of protein, RNA, and RBP-specific UV-crosslinking efficiencies (Supplementary Note 3a-c, Supplementary Methods), which provides a sound basis for optimization of UV-crosslinking conditions and provides useful metrics to verify reproducibility. The robustness and high specificity of LEAP-RBP supports confident validation of RNA-binding and identification of conditions that alter RNA-bound protein abundance (Supplementary Note 4e, f). While LEAP-RBP can be paired with MS-based proteomic approaches to analyze global RNA-bound protein dynamics, SRA and immunoblot provides a cost-effective way to analyze regulation of *in vivo* RNA-binding for individual RBPs of interest. For example, we observed that the TIA-1a isoform (top band) exhibits higher UV-crosslinking efficiency (i.e., total RNA-bound abundance) than the TIA-1b isoform (bottom band) *in vivo* despite comparable total abundance (gold boxes; Supplementary Fig. 2a, Supplementary Note 3c). These data contrast with in vitro studies where it was previously reported that that the RNA-binding properties of TIA-1a and TIA-1b do not differ significantly [29]. These prior studies were performed *in vitro* using established electrophoretic mobility shift assays (EMSA) to analyze interactions between recombinant GST-tagged proteins and two RNA substrates and thus highlight the value in orthogonal experimental approaches to evaluating RNA binding function [30-32]. Curiously, TIA-1a displayed comparable RNA-bound abundance in rat pancreatic insulinoma cell line (832/13) and three human cell lines (i.e., HeLa, 293T, and Huh7) despite it being nearly undetectable in 832/13 input samples (untreated (*N*) vs RNase (*S* + *N*) clRNP fraction, or RNase input; Fig. 7j). These findings suggests that TIA-1a performs comparable RNA-binding functions in 832/13 cells despite its relatively lower total abundance. Like TIA-1, nucleolin (NCL) displays isoform-specific differences in UV-crosslinking efficiency (Source Data Supplementary Fig. 2a). These results illustrate how LEAP-RBP and SRA provide a rapid and cost-effective way to assess the impact of protein modifications and/or alternative splicing isoforms on *in vivo* RNA-binding [33].

LEAP-RBP provides a new methodological approach to the orthogonal validation of observed differences in RNA-bound abundance. As an example, previous studies have reported global changes in RNA-binding activity during cellular stress-responses based on differences in protein recovery (performed by TRAPP, OOPs, XRNAX, RIC/eRIC approaches) [4, 5, 8, 13]. However, the inclusion of proteins with significant free protein contributions and the lack of orthogonal validation showing that observed differences in protein recovery are due to a change in their RNA-bound abundance hampers meaningful interpretation. LEAP-RBP enables the use of SRA as a cost-effective and robust orthogonal validation approach to traditional validation methods such as CLIP-seq or radioisotopic T4 PNK assays [34-36]. Indeed, neither CLIP-seq or radioisotopic T4 PNK assays have been demonstrated to accurately assess changes in RNA-bound protein abundance, thereby compromising their utility for validating observed RBP dynamics. The utility of the LEAP-RBP method and SRA extends to RIP- or CLIP-seq experiments which are typically performed without existing validation of RNA-binding [37, 38]. By providing a cost-effective means to validate *in vivo* RNA-binding activity of putative RBPs, LEAP-RBP is a valuable tool for focusing investigations on high confidence candidates. As a useful example for this suggestion, β-tubulin was identified as UV-enriched by LEAP-RBP, INP, XRNAX, OOPs, Ptex, TRAPP, RIC, and eRIC methods (Supplementary Data 1). Traditionally, this high degree of overlap suggests it's a good candidate for CLIP studies [39]. However, SRA and immunoblot analysis of LEAP-RBP fractions suggests this is a widely observed false positive, perhaps reflecting RNP cargo/motor protein complex association with the microtubule cytoskeleton (Supplementary Fig. 5a).

Beyond these applications, we anticipate LEAP-RBP fractions could serve as a useful starting point for downstream interrogation of RNA-protein interactions. In these approaches, free RNA and protein components of lysates contribute substantial background contamination [40]. LEAP-RBP overcomes these difficulties by removing free RNA, protein, and DNA while allowing scaling of clRNPs in an optimized buffer of choice (Supplementary Note 1).

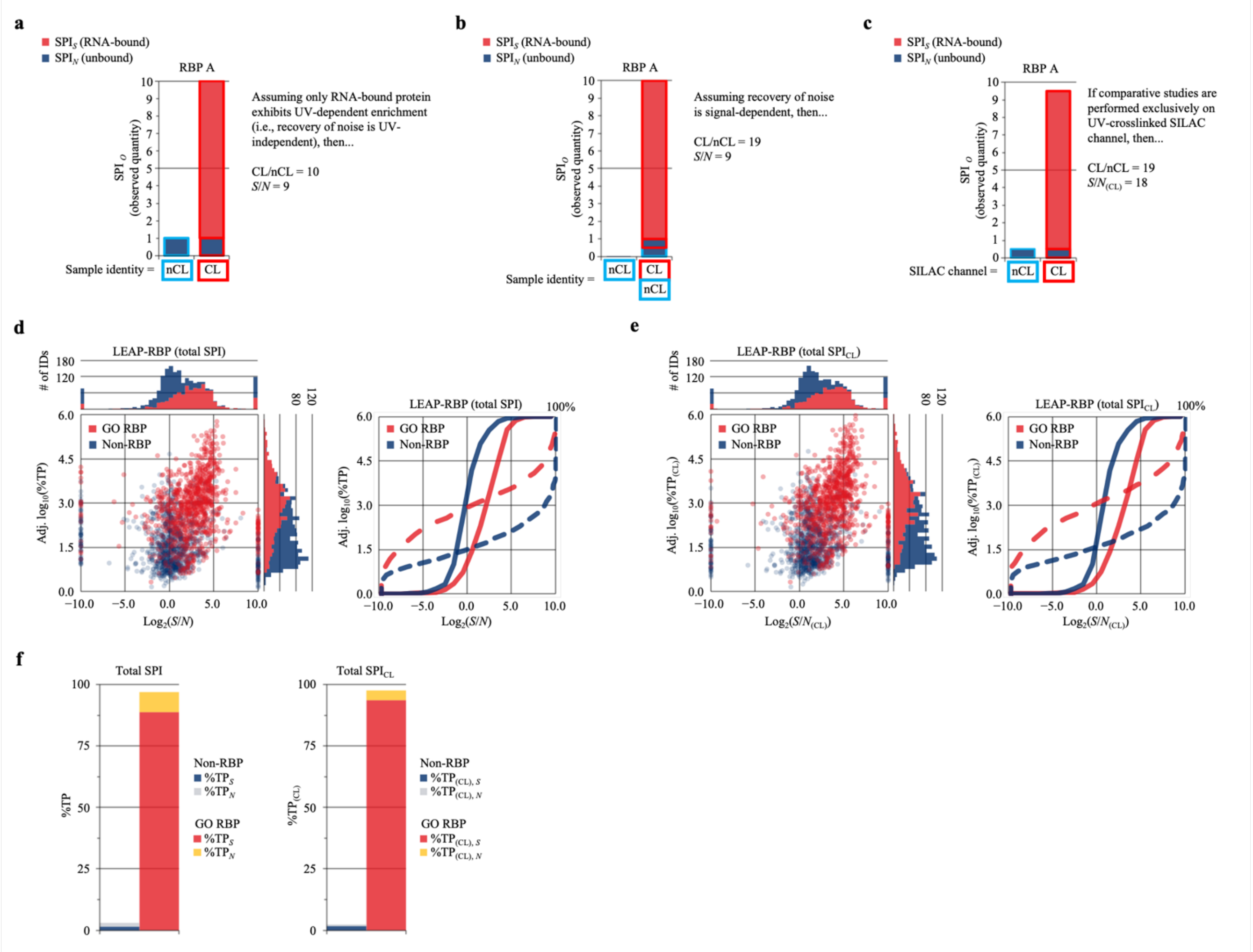

Supplementary Figure 20: Relationship between CL/nCL and *S*/*N* ratios. a, **b** Stacked bar chart showing the expected contributions of RNA-bound (SPI_S) and unbound (SPI_N) counterparts towards the observed quantity (SPI_O) of "RBP A" in UV-crosslinked (CL) and non-crosslinked (nCL) samples assuming recovery of noise is UV-independent (**a**), or signal-dependent (**b**). **c** Stacked bar chart showing the expected change in *S*/*N* of "RBP A" from **b** when performing comparative studies exclusively on the UV-crosslinked SILAC channel assuming recovery of noise is signal-dependent. **d**-**f** Analyses were performed with MS data generated by SILAC LC-MS/MS analysis of LEAP-RBP fractions. **d**, **e** Scatterplots illustrating the difference in protein abundances estimated as a percentage of total SPI (**d**) or total SPI_{CL} (**e**) and *S*/*N* ratios when considering noise contributions from both SILAC channels (**d**) or only the UV-crosslinked SILAC channel (**e**); corresponding frequency curves plotted as a function of $\log_2(S/N)$ (solid) or $\log_{10}(\%TP)$ (dashed) (**d**), or $\log_2(S/N_{(CL)})$ (solid) or $\log_{10}(\%TP_{(CL)})$ (dashed) (**e**). **f** Stacked bar charts showing the estimated $\%TP_S$ and $\%TP_N$ contributions of RBPs and non-RBPs in both SILAC channels (total SPI), or the estimated $\%TP_{(CL),\ S}$ and $\%TP_{(CL),\ N}$ contributions of RBPs and non-RBPs in the UV-crosslinked SILAC channel (total SPI_{CL}). Source data are provided as a Source Data file.

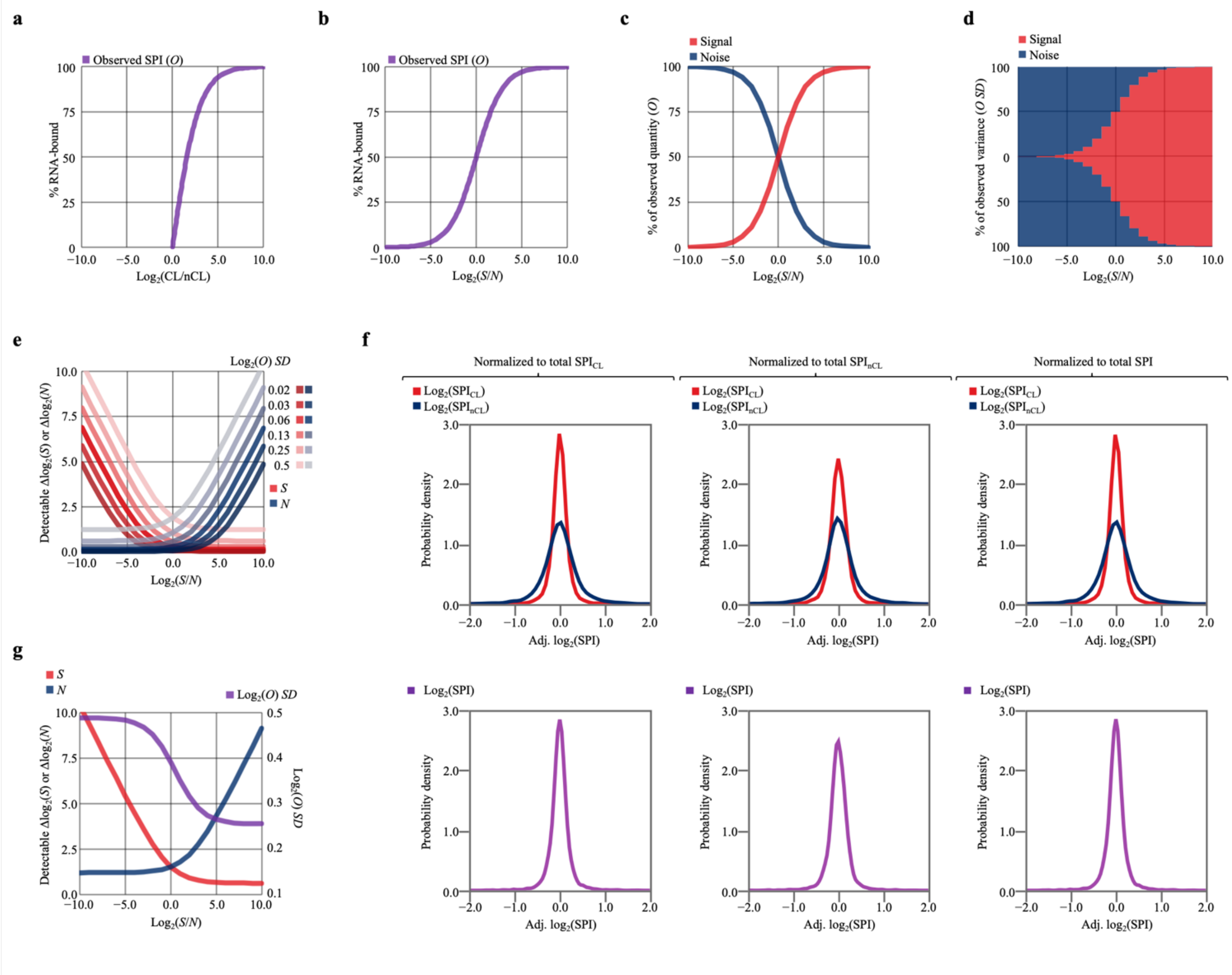

Supplementary Figure 21: *S*/*N* ratios serve as a key metric for identifying differences in observed protein quantity that are more likely to represent a change in $\log_2(S)$. a, **b** Predicted relationship between $\log_2$(CL/nCL) (**a**) or $\log_2(S/N)$ (**b**) and % of observed protein quantity that is RNA-bound. **c**, **d** Predicted relationship between $\log_2(S/N)$ and the % contributions of RNA-bound (*S*) and unbound (*N*) counterparts towards the observed (*O*) protein quantity (**c**) and variance (**d**). **e** Predicted relationship between protein $\log_2(S/N)$ ratios and the detectable $\Delta\log_2(S)$ or $\Delta\log_2(N)$ assuming constant signal or noise respectively; null hypothesis that $\Delta\log_2(O) = 0$. **f** Probability density curves showing the standard deviation (*SD*) of observed protein quantities ($\log_2$(SPI)) in the UV-crosslinked SILAC channel (red), non-crosslinked SILAC channel (blue), or both SILAC channels (purple) when normalizing samples to total MS-intensity of the UV-crosslinked SILAC channel (total SPI_{CL}), non-crosslinked SILAC channel (total SPI_{nCL}), or both SILAC channels (total SPI); analyses were performed with data generated by from SILAC LC-MS/MS analysis of LEAP-RBP fractions. **g** Same as **e**, but detectable $\Delta\log_2(S)$ and $\Delta\log_2(N)$ were estimated with the predicted contributions of RNA-bound and unbound counterparts towards the observed variability of $\log_2(O)$ values (*SD*). Source data are provided as a Source Data file.

Supplementary Note 7

This note provides comparative analysis of proteins identified as UV-enriched* by LEAP-RBP and reference RNA-centric methods.

7a. LEAP-RBP fractions contain many previously identified UV-enriched* proteins.

Many of the proteins identified as UV-enriched* in LEAP-RBP fractions by SILAC LC-MS/MS analysis were identified previously by XRNAX [5], OOPs [13], and pTEX [7] methods as being UV-enriched* (Supplementary Fig. 22a). Most of the proteins with shared UV-enrichment* status (~78%) are GO-annotated as RBPs (Supplementary Fig. 22b). Over 86% of proteins identified as UV-enriched* by RIC and/or eRIC methods were re-identified in LEAP-RBP fractions as UV-enriched* (Supplementary Fig. 22c) [8]. Because GO-annotations of RBPs are largely based on UV-enrichment* status in prior RIC-like experiments [24, 41], the overlap of GO-annotated RBPs largely mirrors the overlap of total UV-enriched* proteins (Supplementary Fig. 22d, Supplementary Note 8b). Remarkably, 94% of GO-annotated mRNA binders (n = 157) identified by RIC and/or eRIC as UV-enriched* were identified in LEAP-RBP fractions as UV-enriched* (Supplementary Fig. 22e). An additional 57 GO-annotated mRNA-binders not identified in the referenced RIC and/or eRIC study were identified as UV-enriched* in LEAP-RBP fractions. These data demonstrate LEAP-RBP can achieve broad UV-enrichment*.

7b. Enhanced *S*/*N* decreases UV-enrichment* specificity for RBPs.

SILAC LC-MS/MS analysis of INP and LEAP-RBP fractions demonstrated that enhanced enrichment of RNA-bound protein (S/N) increases the percentage of total protein in the sample that is RNA-bound (%TP_S) but decreases UV-enrichment* specificity for GO-annotated RBPs (Fig. 5h). This was attributed to a decrease in free protein recovery (%TP_N), lower limit of detection, and UV-enrichment* of low-abundance non-RBPs (Fig. 6c, Supplementary Note 4d, e).

LEAP-RBP displays increased %TP_S compared to other RNA-centric methods (Fig. 5h, 8e, 9c). Therefore, proteins with exclusive LEAP-RBP UV-enrichment* status are predicted to be less abundant (%TP), display lower S/N ratios, and be underrepresented by GO-annotated RBPs. Conversely, proteins with shared UV-enrichment* status are expected to be more abundant (%TP), display higher S/N ratios, and be overrepresented by GO-annotated RBPs. To test these predictions, proteins identified as UV-enriched* in LEAP-RBP fractions were binned based on the number of additional (0–5) RNA-centric methods that identified them as UV-enriched* (Supplementary Fig. 23a); these include the INP method and referenced XRNAX [5], OOPs [6], Ptex [7], and RIC [8] studies. The abundance ($\log_{10}$(%TP)) of protein IDs from each bin were analyzed as a function of their $\log_2(S/N)$ ratios (Supplementary Fig. 23b). As expected, proteins with shared UV-enrichment* status were of higher abundance, displayed higher S/N ratios, and were overrepresented by RBPs (+5 methods; Supplementary Fig. 23a, b). Similar trends were observed when evaluating the abundances and enrichment efficiencies (S/N) of proteins with shared UV-enriched* status in any of the referenced MS datasets (Supplementary Fig. 24a, b). Conversely, those with exclusive LEAP-RBP UV-enrichment* status were less abundant, displayed lower S/N ratios, and were underrepresented by RBPs (+0 methods; Supplementary Fig. 23a, b). While proteins identified exclusively by other methods as being UV-enriched* were underrepresented by RBPs (Supplementary Fig. 25a), they were not appreciably less abundant (Supplementary Fig. 25b). These data support the view that increased method specificity results in significant UV-enrichment* of low-abundance RBPs, and reillustrates the main points of Supplementary Note 4e discussed below (Supplementary Note 7c). Comparative analyses of total protein IDs and total UV-enriched* protein IDs identified by each method are shown in Supplementary Fig. 26 and Supplementary Fig. 27, respectively. Full MS datasets for LEAP-RBP, INP, XRNAX [5], OOPs [6], Ptex [7], TRAPP [4], and RIC [8] can be found in Supplementary Data 3, 5, 10-15 respectively. Uniprot IDs for each of the protein groups analyzed in Supplementary Note 7b are included in Supplementary Table 7. Additional information on the analyses presented in Supplementary Fig. 23-27 were provided in the Supplementary Methods. Summary statistics (e.g., average $\log_2(S/N)$ ratios, # of protein IDs, # of UV-enriched* IDs, %TP contributions, etc.) for each of the categories (e.g., unique UV-enriched*, shared UV-enriched*) were made available in the provided MS datasets. For additional analyses of MS datasets, see Supplementary Note 7a, b.

7c. GO-analysis of protein IDs with exclusive LEAP-RBP UV-enrichment* status identifies many metabolic enzymes.

GO-analysis was performed on proteins with exclusive LEAP-RBP UV-enrichment* status ($n = 293$) or those with shared UV-enrichment* status ($n = 257$) (Supplementary Fig. 28a-d). As expected, proteins with shared UV-enrichment* status were highly enriched for RNA-related functions and processes (Supplementary Fig. 28a, **c**). Conversly, proteins with exclusive LEAP-RBP UV-enrichment* status were enriched for catalytic activities and metabolic processes (Supplementary Fig. 28b, d). Indeed, many proteins with exclusive LEAP-RBP UV-enrichment* status are metabolic enzymes (Supplementary Fig. 29a, b). Although their observed UV-enrichment* merits consideration as bona fide RNA-binding proteins, their low enrichment (*S*/*N*) and abundance (%TP) should also be considered. Indeed, many non-RBPs identified as UV-enriched* in LEAP-RBP fractions by SILAC LC-MS/MS were undetected by SRA and immunoblot (Supplementary Fig. 5a). To this point, there has been a recent expansion in the number of UV-enriched* putative RBPs including metabolic enzymes, regulatory kinases, and others without previously known RNA-binding functions and/or which lack canonical RNA binding domains [42-44]. Intriguing as these findings may be, without rigorous assessment of their enrichment efficiencies (*S*/*N*) and abundances (%TP), it remains unclear if these findings are biologically relevant. In this study, we speculate that low-abundance, non-specific UV-crosslinking of non-RBPs to non-RNA substrates may result in their significant UV-enrichment* in LEAP-RBP fractions. In principle, RNA-centric enrichment of RNA-bound protein can be viewed as a purification process aimed at the selective enrichment of proteins bound to sizable polyanions. However, UV-crosslinking of non-RBPs to other negatively charged small molecules (e.g., nicotinamide- and flavin- bearing dinucleotides) which are common co-factors of dehydrogenases could endow similar physicochemical properties [36, 45]; given the high enrichment efficiency and sensitivity of the LEAP-RBP method, this may result in their significant, albeit lowly efficient, UV-enrichment*.

Current high throughput methods for validation of UV-enriched* putative RBPs involve partial trypic-digestion of RNP fractions and TiO_2/SiO_2 or affinity-based enrichment of RNA-bound peptides [5, 13, 21, 46]. However, UV-enriched* proteins identified using these approaches include those which were undetectable in LEAP-RBP fractions by SRA and immunoblot (e.g., GRP78, GRP94, GAPDH; Supplementary Fig. 5a, Supplementary Data 1). While considerably lower throughput, LEAP-RBP and SRA provide necessary orthogonal evidence of direct RNA-binding; RNase-dependent mobility shifts to the molecular weight of the unbound counterpart during SDS-PAGE is only expected for proteins bound to sizable RNA substrates through a single UV-crosslinking event (RNA-protein). Although the probability of multiple UV-crosslinked events is presumably lower than single UV-crosslinking events, rigorous testing of this assumption *in vivo* is lacking and likely depends on the RBP of interest.

7d. LEAP-RBP and SRA reveal discordance with *in vitro* validation methods.

Despite several studies demonstrating its RNA-binding potential, we were unable to validate RNA-binding activity for GAPDH [44, 47]. In prior studies, GAPDH was found to bind wild type $tRNA^{Met}$ in HeLa cells but not a mutant $tRNA^{Met}$ version defective in nucleocytoplasmic transport [48]. GAPDH was later found to exhibit increased binding to AU-rich elements on colony-stimulating factor-1 (CSF-1) mRNA in malignant (Hey) ovarian epithelial cells compared to normal (NOSE.1) ovarian epithelial cells [49]. However, the interactions between GAPDH and RNA species were all observed post-lysis, in non-cellular contexts. Given the critical importance of buffer conditions for maintaining RNA-protein complexes during *in vitro* mobility-shift assays, RNA-protein interactions observed in vitro provide supportive but not conclusive evidence of an *in situ* RNA-binding function [50, 51]. In contrast, UV-crosslinking provides a way to stabilize physiologically relevant RNA-protein interactions occurring *in vivo* at zero-order distances [18, 52]. As demonstrated in this study, direct UV-crosslinking of RNA to protein via a single UV-crosslinking events is highly specific for RBPs. Because LEAP-RBP recovers near 100% of RNA-bound protein (Supplementary Note 3), our inability to detect GAPDH-RNA complexes is unlikely to reflect a unique bias in the isolation of RNA-bound proteins. Consistent with this view, we observed that GAPDH behaved similarly to other non-RBPs during repeated AGPC extraction (Fig. 1c). While some amino acids are known to be less reactive towards RNA during UV-crosslinking, all should be considered susceptible to UV-crosslinking [52, 53]. We were able to identify RBPs in LEAP-RBP fractions displaying UV-crosslinking efficiencies <0.3% by SRA analysis (e.g., RPN1; Supplementary Fig. 2a). Because many well-established RBPs were found to have UV-

crosslinking efficiencies between 1-20% (0.4 J/cm^2, 254 nm), the physiological relevance of proteins in LEAP-RBP fractions undetected by SRA and immunoblot is, in our view, questionable. Lastly, LEAP-RBP fractions isolated from HeLa cells that were UV-crosslinked under different conditions (e.g., without media removal and/or incubation on ice) display comparable RNase-sensitive profiles by SRA and Coomassie Blue (Protein) staining (Supplementary Fig. 13e, Supplementary Note 3b). Therefore, the inability to detect GAPDH as RNase-sensitive by SRA and immunoblot is unlikely to be caused by UV-crosslinking or methodological artifacts. Indeed, if GAPDH were detected in LEAP-RBP fractions, it's not expected to appear RNase-sensitive ($S/N = 0.1$). While GAPDH is routinely scored as UV-enriched* during RNA-centric RBP-capture experiments, it typically displays lower S/N (Supplementary Data 1). For example, while GAPDH was identified as UV-enriched* in the original RIC publication [24], it was not identified as UV-enriched* during the RIC experiment referenced in this study (non-SILAC, $S/N = 0.5$) [8]. These data again demonstrate the utility of S/N-based analysis to assess RBP confidence and the ability of LEAP-RBP and SRA to rigorously validate RNA-binding occurring *in vivo*.

7e. Non-SILAC comparison of RNP fractions isolated from UV-crosslinked and non-crosslinked cells results in UV-enrichment of free proteins evidenced by non-specific %TP$_{(S)}$ contributions.

Overlap analysis of proteins identified in INP and LEAP-RBP by SILAC LC-MS/MS analysis showed many background proteins exclusively identified in INP fractions and displaying a $\log_2$(CL/nCL) ratios with a mean distribution of 0 (Supplementary Note 4d). Comparison of INP fractions isolated from UV-crosslinked or non-crosslinked cells showed high UV-dependent enrichment of these background proteins which appear as "RNase-insensitive" bands by SRA and Coomassie Blue (protein) staining (blue boxes; Supplementary Fig. 14f). Therefore, non-SILAC comparison of INP fractions isolated from independently processed UV-crosslinked and non-crosslinked cells would likely result in their apparent UV-enrichment*. Because most of these background proteins are non-RBPs (329/391), this is expected to decrease UV-enrichment* specificity (Supplementary Fig. 14e). However, unlike the decrease in UV-enrichment* specificity caused by enhanced S/N and high %TP$_S$, a decrease in UV-enrichment* specificity caused by UV-dependent enrichment of free protein is evidenced by non-specific (non-RBP) %TP$_{(S)}$ contributions. This can be explained by the following:

1) UV-crosslinking is inefficient, with an estimated 0.5–2.5% of the total protein being crosslinked to RNA depending on the UV-dose (0.1–0.8 J/cm^2, 254 nm; Supplementary Note 3a-c); even for the most abundant RBPs identified in LEAP-RBP fractions by SILAC LC-MS/MS (%TP rank), UV-crosslinking (0.4 J/cm^2, 254 nm) efficiency was estimated between 10–20% (gold boxes; Supplementary Fig. 2a). Therefore, relative %TP contributions of RBPs vs non-RBPs in input samples (i.e., total protein) is more reflective of their relative free protein abundances than relative RNA-bound abundances.

Input (total protein):

$\%TP_{(N),\ RBPs}/\%TP_{(N),\ non\text{-}RBPs} \approx \%TP_{RBPs}/\%TP_{non\text{-}RBPs} < \%TP_{(S),\ RBPs}/\%TP_{(S),\ non\text{-}RBPs}$

2) Based on SILAC LC-MS/MS analysis of INP (%TP$_S$ = 47) and LEAP-RBP (%TP$_S$ = 91) fractions, an estimated 98.3–98.6% of the total RNA-bound protein (%TP$_{(S)}$) in either fraction is contributed by GO-annotated RBPs (%TP$_{(S),\ non\text{-}RBPs}$ < 2; Source Data Fig. 5h). Similar non-specific %TP$_{(S)}$ contributions were observed for other RNA-centric methods utilizing SILAC LC-MS/MS approaches: 2.6 for XRNAX and ~5.0 for TRAPP (Source Data Fig. 8e, 9c, Supplementary Note 8b) [4, 5]. Therefore, differences in relative %TP$_{(S)}$ contributions of RBPs and non-RBPs is likely indicative of high UV-crosslinking specificity.

Input (total protein):

$\%TP_{(N),\ RBPs}/\%TP_{(N),\ non\text{-}RBPs} < \%TP_{(S),\ RBPs}/\%TP_{(S),\ non\text{-}RBPs}$

3) UV-dependent enrichment of free protein and signal-dependent recovery of noise are widely observed phenomenon; both can be observed by SDS-PAGE when comparing equivalent amounts (% fraction) of RNase-treated and untreated RNP fractions isolated from UV-crosslinked and non-crosslinked samples (Supplementary Note 4); repeated AGPC extraction: Fig. 1b; INP: Supplementary Fig. 9f; LEAP-RBP: Supplementary Fig. 6c; XRNAX, OOPs, and Ptex: Fig. 8b, c; RIC and TRAPP: gold boxes, Source Data Fig.

8c. Free protein exhibiting UV-dependent enrichment during non-SILAC comparison will be perceived as false signal. This is expected to overestimate *S*/*N* ratios and $\%TP_S$ contributions of both RBPs and non-RBPs:

False $\%TP_{(S)}$ = false signal; (false $\%TP_{(S), \text{RBPs}}$ + $\%TP_{(S), \text{RBPs}}$)/(false $\%TP_{(S), \text{non-RBPs}}$ + $\%TP_{(S), \text{non-RBPs}}$)

Depending on method specificity ($\%TP_S$) and interactions between UV-crosslinking, method-specific RNA-enrichment conditions, and protein-specific physicochemical properties (Supplementary Note 4c), the relative false $\%TP_{(S)}$ contributions of RBPs vs non-RBPs is expected to vary. However, given the large difference between their relative $\%TP_{(S)}$ and $\%TP_{(N)}$ contributions in input samples, the following is likely:

$\%TP_{(N), \text{RBPs}}/\%TP_{(N), \text{non-RBPs}}$ < false $\%TP_{(S), \text{RBPs}}$/false $\%TP_{(S), \text{non-RBPs}}$ < $\%TP_{(S), \text{RBPs}}/\%TP_{(S), \text{non-RBPs}}$

Affirmatively, non-specific $\%TP_{(S)}$ contributions for non-SILAC experiments were discernibly higher: 24.4 for OOPs and 28.4 for Ptex fractions [6, 7]. However, non-specific $\%TP_{(S)}$ contributions for the referenced RIC study (non-SILAC) were only 1.5% [8]. This was attributed to high $\%TP_S$ of the RIC method and the observation that current GO-annotations of RBPs are largely based on their UV-enrichment* status in prior RIC-like (non-SILAC) experiments (Supplementary Note 8b).

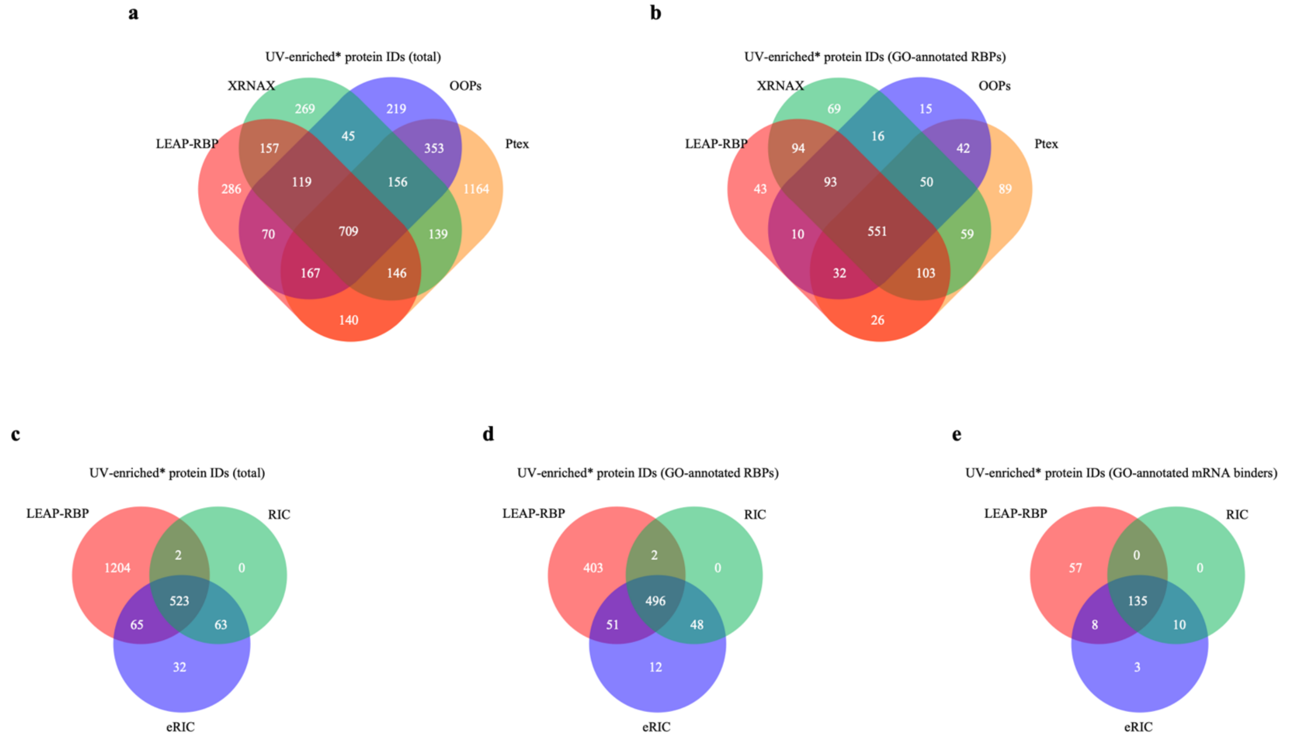

Supplementary Figure 22: LEAP-RBP fractions contain many proteins previously identified as UV-enriched*. a, **b** Venn diagrams showing overlap of total proteins (**a**) or GO-annotated RBPs (**b**) identified as UV-enriched* by LEAP-RBP and RNA-centric methods: XRNAX [5], OOPs [13], and Ptex 1.5 [7]. **c**-**e** Venn diagrams showing overlap of total protein IDs (**c**), GO-annotated RBPs (**d**), or GO-annotated mRNA binders (**e**) identified as UV-enriched* by LEAP-RBP and referenced RIC and eRIC studies [8]. Source data are provided as a Source Data file.

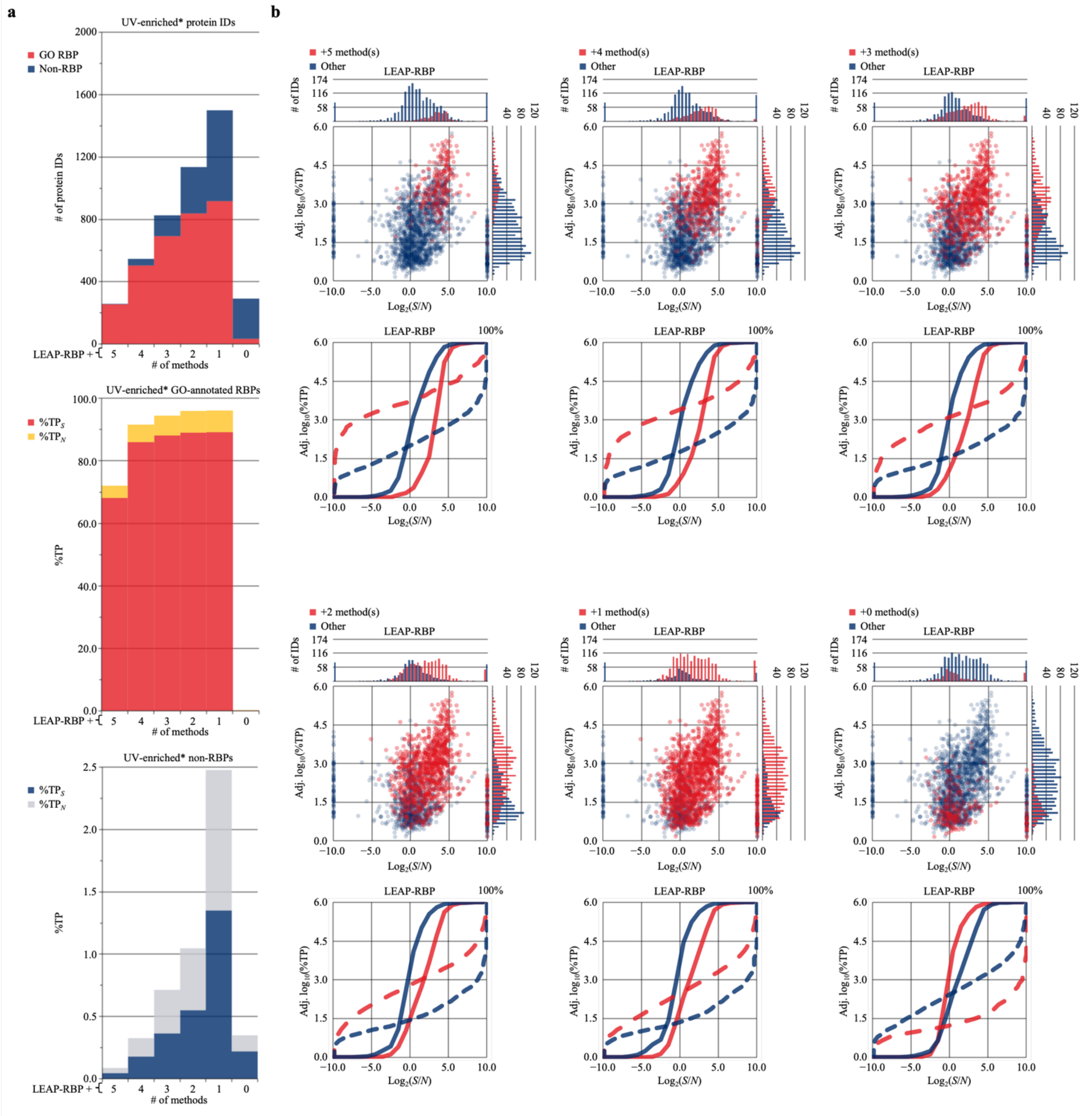

Supplementary Figure 23: Enhanced S/N results in significant UV-enrichment* of low abundance non-RBPs. a, **b** Analyses were performed with MS data generated by SILAC LC-MS/MS analysis of LEAP-RBP fractions, and the reported UV-enrichment* status of protein IDs identified by the INP method and referenced XRNAX [5], OOPs [6], Ptex [7], and RIC [8] studies (Methods). **a** Stacked bar charts showing the number of RBPs and non-RBPs identified as UV-enriched* by LEAP-RBP + 0–5 additional methods, or the estimated $\%TP_S$ and $\%TP_N$ contributions of RBPs, or non-RBPs, identified as UV-enriched* by LEAP-RBP + 0–5 additional methods in LEAP-RBP fractions. **b** Scatterplots illustrating the difference in protein abundance ($\log_{10}$(%TP)) and $\log_2(S/N)$ ratios of proteins from each group shown in **a.** Corresponding frequency curves plotted as a function of $\log_2(S/N)$ (solid) or $\log_{10}$(%TP) (dashed). Source data are provided as a Source Data file.

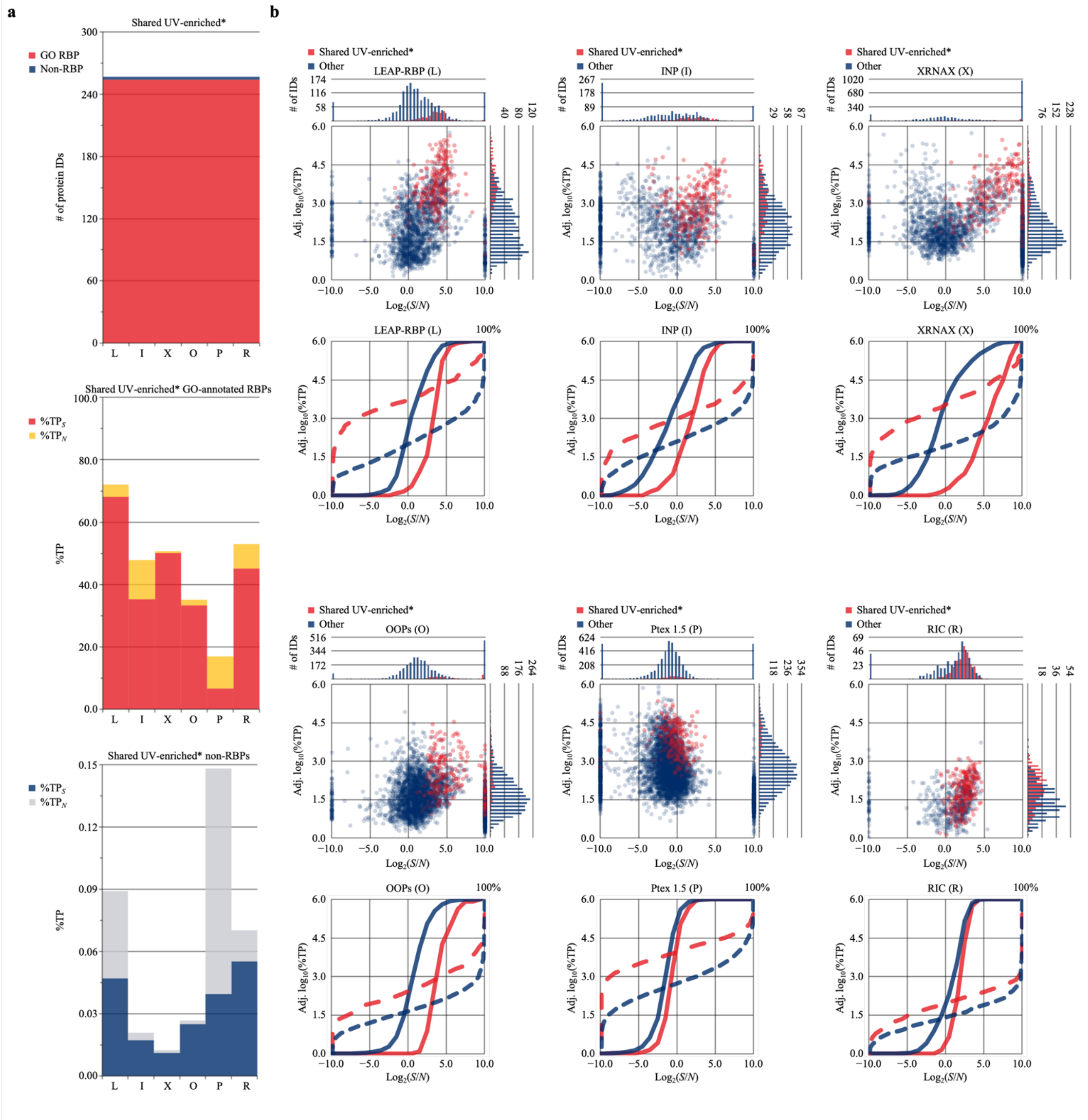

Supplementary Figure 24: Proteins with shared UV-enrichment* status are more abundant and display higher *S*/*N* ratios in RNP fractions. a, **b** Analyses were performed with MS data from LEAP-RBP or INP SILAC LC-MS/MS experiments or with available MS data from referenced XRNAX (SILAC) [5], OOPs (non-SILAC) [6], Ptex (non-SILAC) [7], and RIC (non-SILAC) [8] studies (Method**s**). **a** Stacked bar charts showing the number of RBPs and non-RBPs with shared UV-enrichment* status, or the estimated %TP$_S$ and %TP$_N$ contributions of RBPs, or non-RBPs, with shared UV-enrichment* status in each RNP fractions. **b** Scatterplots illustrating the higher relative abundance (log$_{10}$(%TP)) and enrichment (log$_2$(*S*/*N*)) of proteins with shared UV-enrichment* status in LEAP-RBP, INP, XRNAX, OOPs, Ptex, or RIC fractions. Corresponding frequency curves plotted as a function of log$_2$() (solid) or log$_{10}$(%TP) (dashed). Source data are provided as a Source Data file.

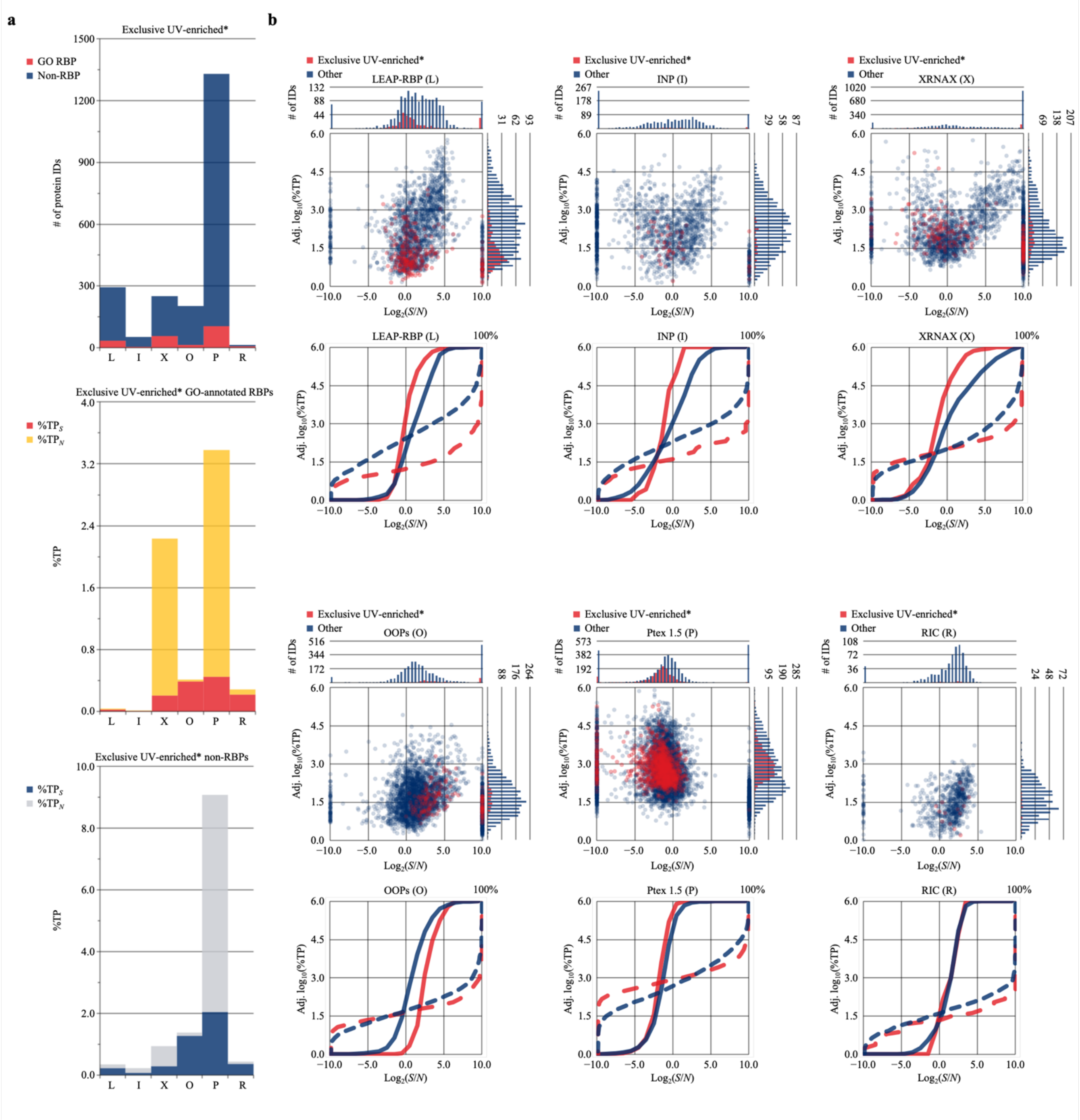

Supplementary Figure 25: Proteins with exclusive UV-enrichment* status are overrepresented by non-RBPs. a, **b** Analyses were performed with MS data from LEAP-RBP or INP SILAC LC-MS/MS experiments or with available MS data from referenced XRNAX (SILAC) [5], OOPs (non-SILAC) [6], Ptex (non-SILAC) [7], and RIC (non-SILAC) [8] studies (Methods). **a** Stacked bar charts showing the number of RBPs and non-RBPs with exclusive UV-enrichment* status identified by each method, or the estimated $\%TP_S$ and $\%TP_N$ contributions of RBPs, or non-RBPs, with exclusive UV-enrichment* status in each RNP fraction. **b** Scatterplots illustrating the differences in abundance ($\log_{10}(\%TP)$) and enrichment ($\log_2(S/N)$) of proteins with exclusive UV-enrichment* status in LEAP-RBP, INP, XRNAX, OOPs, Ptex, or RIC fractions. Corresponding frequency curves plotted as a function of $\log_2(S/N)$ (solid) or $\log_{10}(\%TP)$ (dashed). Source data are provided as a Source Data file.

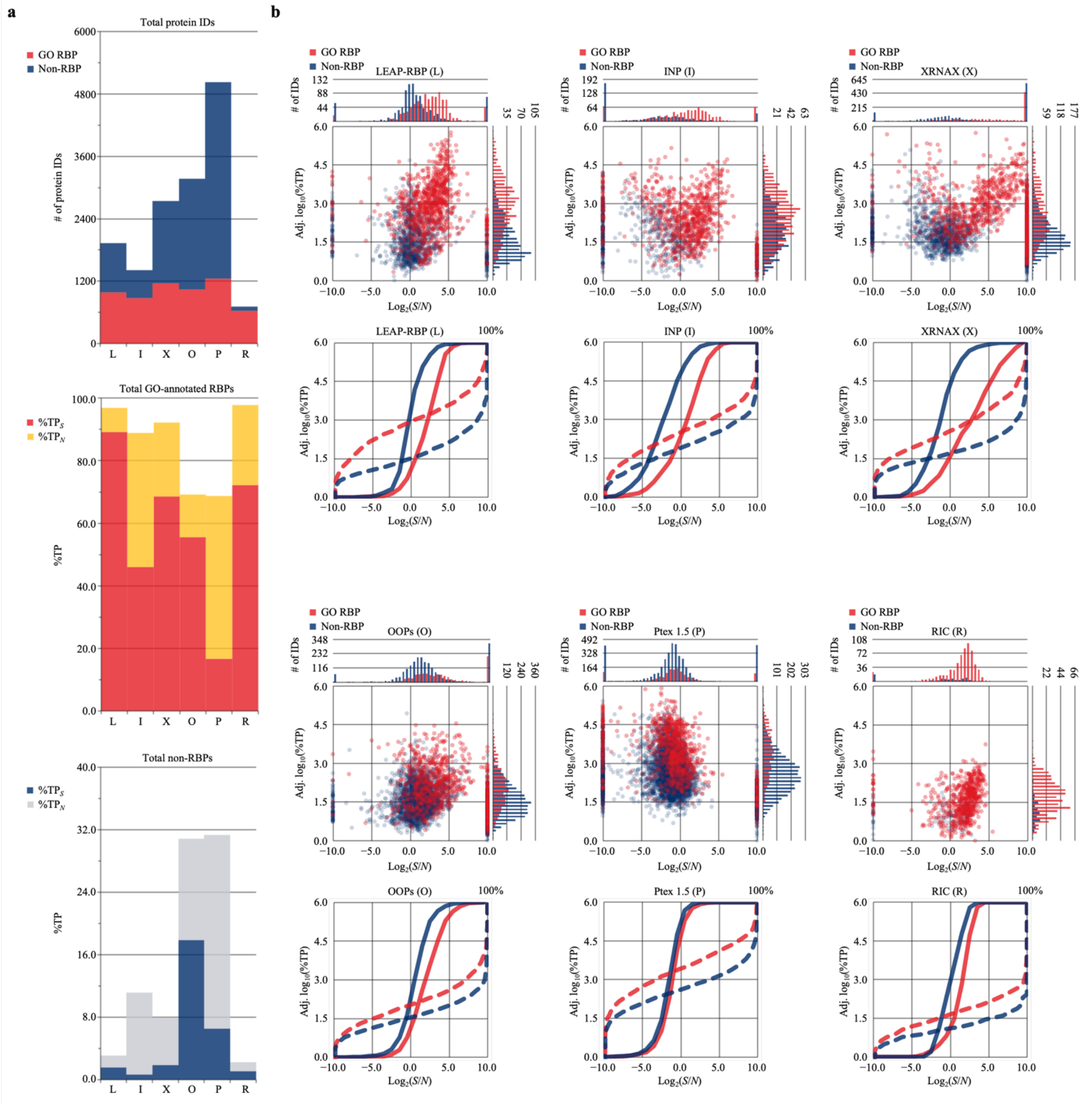

Supplementary Figure 26: Comparative analysis of total protein IDs identified by each method. a, **b** Analyses were performed with MS data from LEAP-RBP or INP SILAC LC-MS/MS experiments or with available MS data from referenced XRNAX (SILAC) [5], OOPs (non-SILAC) [6], Ptex (non-SILAC) [7], and RIC (non-SILAC) [8] studies (Method**s**). **a** Stacked bar charts showing the number of RBPs and non-RBPs identified by each method, or the estimated $\%TP_S$ and $\%TP_N$ contributions of RBPs, or non-RBPs, in each RNP fraction. **b** Scatterplots illustrating the difference in protein abundance ($\log_{10}$(%TP)) and enrichment ($\log_2(S/N)$) of GO-annotated RBPs and non-RBPs identified by each method. Corresponding frequency curves plotted as a function of $\log_2(S/N)$ (solid) or $\log_{10}$(%TP) (dashed). Source data are provided as a Source Data file.

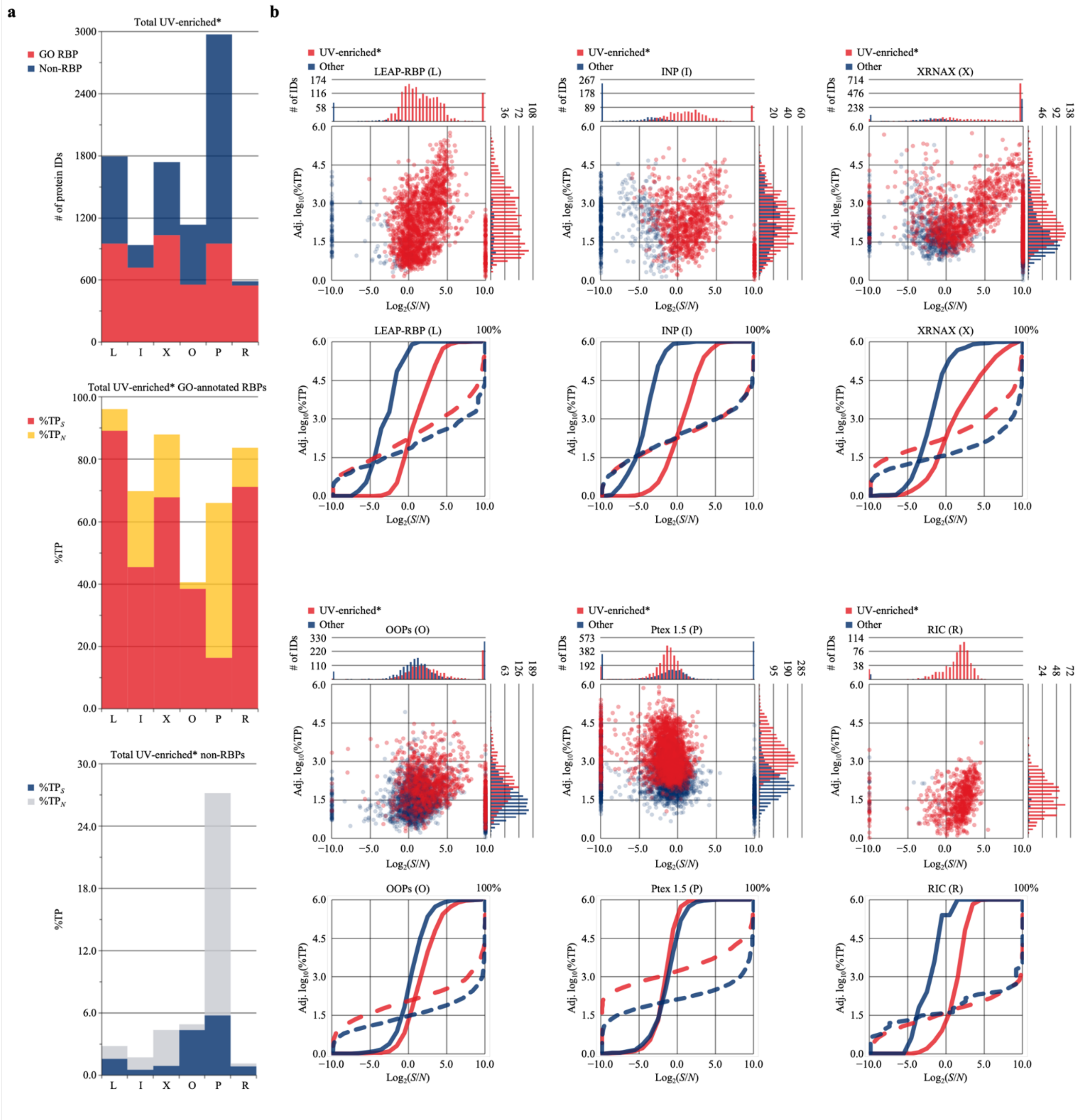

Supplementary Figure 27: Comparative analysis of total protein IDs identified as UV-enriched* by each method. a, **b** Analyses were performed with MS data from LEAP-RBP or INP SILAC LC-MS/MS experiments or with available MS data from referenced XRNAX (SILAC) [5], OOPs (non-SILAC) [6], Ptex (non-SILAC) [7], and RIC (non-SILAC) [8] studies (Methods). **a** Stacked bar charts showing the number of RBPs and non-RBPs identified as UV-enriched* by each method, or the estimated %TP_S and %TP_N contributions of RBPs, or non-RBPs, identified as UV-enriched* in each RNP fraction. **b** Scatterplots illustrating the difference in protein abundance ($\log_{10}$(%TP)) and enrichment ($\log_2(S/N)$) of proteins identified as UV-enriched* by each method. Corresponding frequency curves plotted as a function of $\log_2(S/N)$ (solid) or $\log_{10}$(%TP) (dashed). Source data are provided as a Source Data file.

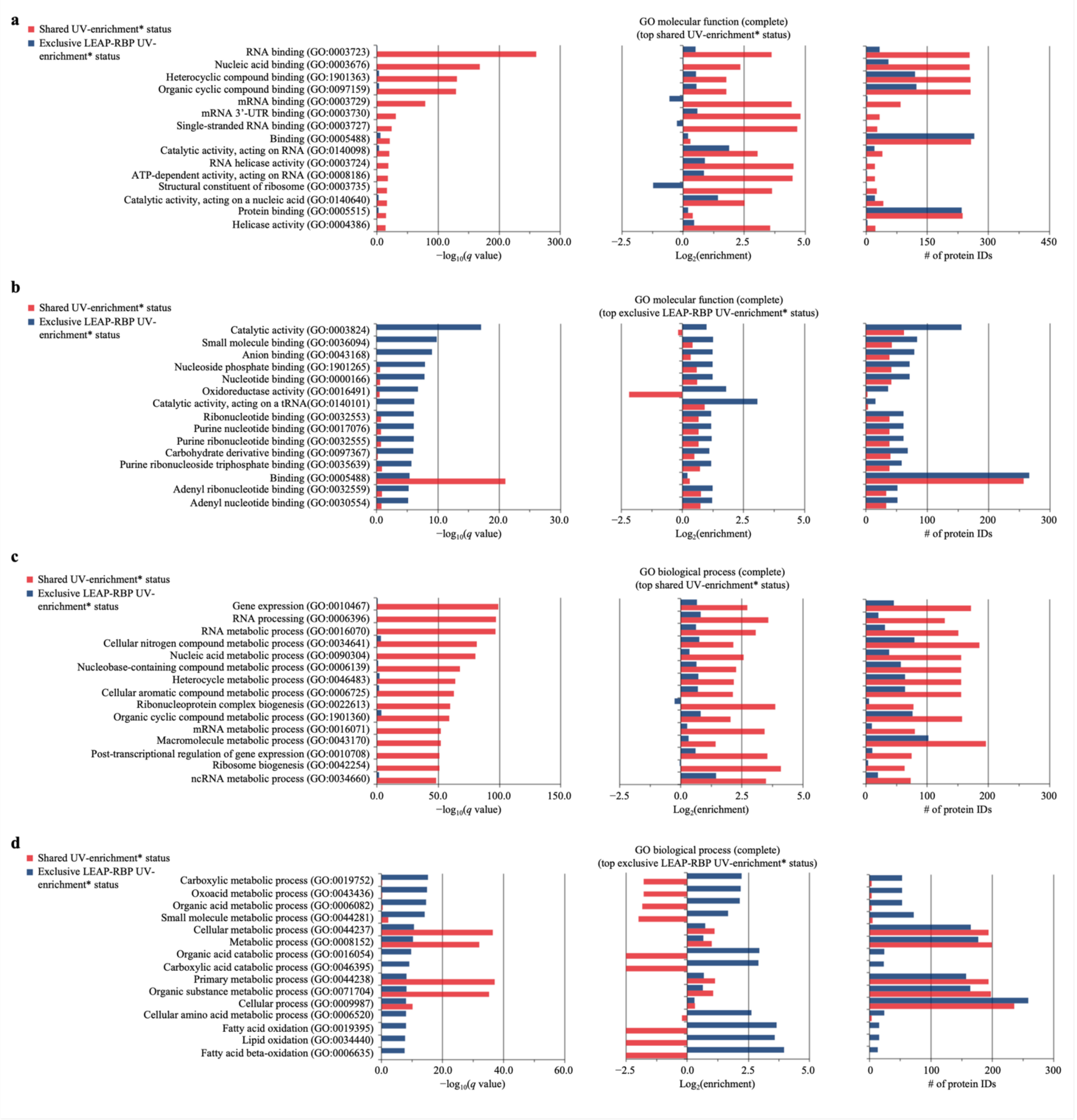

Supplementary Figure 28: GO-analysis of protein IDs with exclusive LEAP-RBP UV-enrichment* identifies proteins involved in organic acid metabolism. a-d GO-analysis was performed on assigned Uniprot IDs of proteins with exclusive LEAP-RBP UV-enrichment* status or shared UV-enrichment* status (LEAP-RBP and the 5 referenced studies) from Supplementary Figure 23; Uniprot IDs were made available in Supplementary Data 7; Fisher's Exact, two-tailed, correction for multiple hypothesis testing performed using the Benjamini-Hochberg approach and a false-discovery rate of 5%. **a**, **b** $-\log_{10}(q$ value), $\log_2$(enrichment), and number of protein IDs for the top 15 enriched GO terms for proteins with shared (**a**) or exclusive LEAP-RBP (**b**) UV-enrichment* status. **c**, **d** $-\log_{10}(q$ value), $\log_2$(enrichment), and number of protein IDs for the top 15 enriched GO terms for proteins with shared (**c**) or exclusive (**d**) UV-enrichment* status. Source data are provided as a Source Data file.

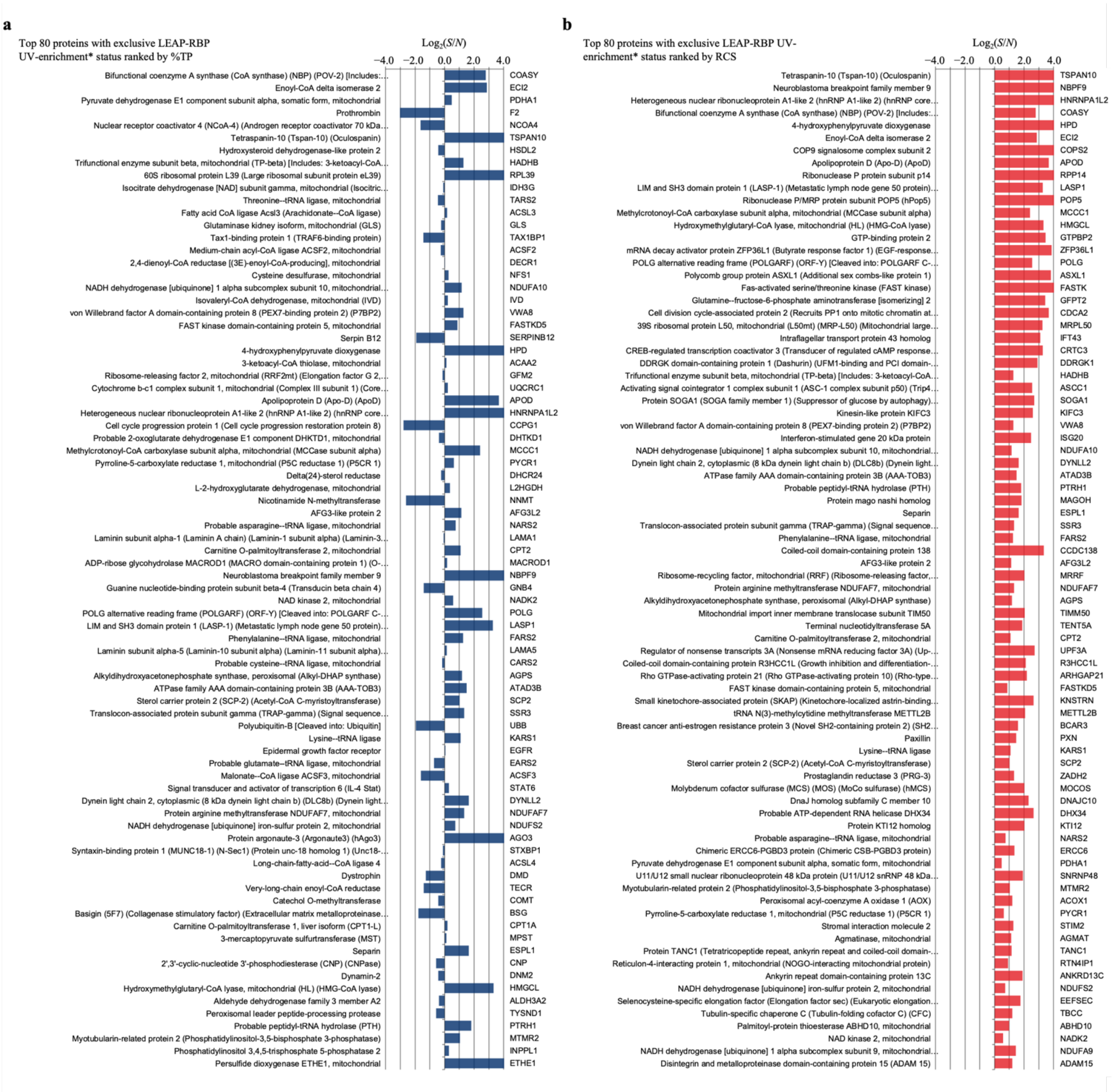

Supplementary Figure 29: Proteins with exclusive LEAP-RBP UV-enrichment* status are involved in organic acid metabolism and many lack RNA-related GO-annotations. a, b $\log_2(S/N)$ ratios of the top exclusive LEAP-RBP UV-enriched* protein IDs ranked by abundance (%TP) (**a**) or RCS (**b**). Primary gene names and protein names are included on the right and left of each bar chart, respectively. Full lists of Uniprot IDs were made available in Supplementary Data 7; RCS, %TP, $\log_2(S/N)$ ratios and other metric were included in Supplementary Data 3. Source data are provided as a Source Data file.

Supplementary Note 8
This note provides extended *S*/*N*-based analysis of LEAP-RBP, INP, and referenced RNA-centric methods.

8a. SRA and SILAC LC-MS/MS serve as complementary "*S*/*N*-based" analytical approaches when evaluating method specificity for RNA-bound RBPs.
In this study, *S*/*N* and $\%TP_S$ serve as key metrics for evaluating RNA-bound protein enrichment and method specificity for RNA-bound RBPs. Both SRA and SILAC LC-MS/MS are considered "*S*/*N*-based" analytical approaches because they distinguish RNA-bound proteins from their unbound counterparts and evaluate *S*/*N*. Comparison of INP ($\%TP_S = 47$) and LEAP-RBP ($\%TP_S = 91$) fractions by SRA and SILAC LC-MS/MS analysis identified distinguishing features for methods with high specificity for RNA-bound RBPs. This primarily includes a lack of background proteins ($S = 0$) appearing as RNase-insensitive bands by SRA and Coomassie Blue (protein) staining or as a distribution with mean $\log_2$(CL/nCL) ratios of 0 by SILAC LC-MS/MS analysis (Fig. 2a, b, 4f, Supplementary Note 4d). When plotting the abundance (%TP) of proteins identified by SILAC LC-MS/MS analysis as a function of their $\log_2(S/N)$ ratios, methods with high $\%TP_S$ (e.g., LEAP-RBP) are distinguishable from methods with low $\%TP_S$ (e.g., INP) by the lack of high abundance proteins displaying negatives $\log_2(S/N)$ ratios (blue boxes on scatterplots; Supplementary Fig. 30a). The observed quantities for proteins displaying positive or negative $\log_2(S/N)$ ratios are thus more representative of their RNA-bound or free protein quantities respectively (Supplementary Note 6b). GO-annotated RBPs contribute >98% of the total RNA-bound protein in INP and LEAP-RBP fractions ($\%TP_{(S)}$; Source Data Fig. 5h), likely reflecting high UV-crosslinking specificity for RNA-binding proteins. When $\%TP_S = 100\%$, $\%TP_S = \%TP_{(S)}$ and observed protein abundances will reflect their true RNA-bound abundance (Supplementary Note 4e, f, 6a). The relative $\%TP_{(S)}$ contributions of RBPs vs non-RBPs is higher than their relative $\%TP_{(N)}$ contributions in input or RNP fractions (Supplementary Note 7e). Therefore, methods with high $\%TP_S$ (e.g., LEAP-RBP) are distinguished from methods with low $\%TP_S$ (e.g., INP) by an increase in the abundance (%TP) of RBPs (red) as compared to non-RBPs (blue) (gold boxes on scatterplots or dashed lines on cumulative frequency curves; Supplementary Fig. 30a). Contrarily, enhanced *S*/*N* was found to result in higher enrichment efficiencies of both RBPs and non-RBPs despite continued differences in their enrichment efficiencies. Therefore, methods with high $\%TP_S$ (e.g., LEAP-RBP) are distinguishable from methods with low $\%TP_S$ (e.g., INP) by the enhanced enrichment of both RBPs and non-RBPs (red and blue solid lines on cumulative frequency curves; Supplementary Fig. 30a).

RBP confidence score ranking exploits the observed differences in UV-enrichment efficiency (*S*/*N*) and abundance (%TP) of GO-annotated RBPs to distinguish them from non-RBP (Fig. 6e-g). As noted in the previous section, the relative abundance of RBPs as compared to non-RBPs is dependent on $\%TP_S$. Therefore, RCS ranking is more accurate for methods with high specificity for RNA-bound proteins ($\%TP_S$). For example, many of the RNA-binding proteins (red text) identified as RNase-sensitive in LEAP-RBP fractions (high $\%TP_S$) by SRA and immunoblot (e.g., NCL, TRAPα, and RPN1; Fig. 2b), displayed $\log_2(S/N)$ ratios less than 0 in INP fractions (low $\%TP_S$) by SILAC LC-MS/MS (Supplementary Fig. 30b). Because RCS ranking automatically places proteins with negative $\log_2(S/N)$ ratios at lower ordinal rank (Fig. 6f, g, Supplementary Methods), these would be considered low confidence RBPs (low RCS; Supplementary Fig. 30c). The placement of proteins displaying negative $\log_2(S/N)$ ratios at lower ordinal rank is important for comparative studies because their observed abundance is more representative of their free protein abundance (Supplementary Note 6b, c). However, the placement of GO-annotated RBPs at lower RCS rank is indicative of low specificity, as most RBPs display positive $\log_2(S/N)$ in LEAP-RBP fractions (high $\%TP_S$). Therefore, the ability of both RCS and %TP ranking to accurately place GO-annotated RBPs at higher ordinal rank than non-RBPs is indicative of high $\%TP_S$ (Supplementary Fig. 30d, e).

Comparison of observed protein quantities (SPI) between biological replicates after mean-normalized to total SPI illustrates the expected correlation for proteomic samples with similar protein profiles (i.e., relative protein quantities or $\Delta\log_{10}$(SPI); Supplementary Fig. 31a, Supplementary Methods). Conversely, observed protein profiles in the UV-crosslinked (SPI_{CL}) and non-crosslinked (SPI_{nCL}) SILAC channels are dissimilar (Supplementary Fig. 31b). This is expected; the observed protein quantities in the UV-crosslinked SILAC channel represent RNA-bound and free protein quantities ($S + N$) while observed protein quantities in the non-crosslinked SILAC channel represent free protein quantities (*N*; Supplementary Note 5). Therefore, the profile

of proteins displaying high RCS (high S/N) are expected to be more dissimilar: $(S + N)_{CL} > N_{nCL}$. Conversely, the profile of proteins displaying low RCS (low S/N) are expected to be more similar: $(S + N)_{CL} \approx N_{CL} \approx N_{nCL}$. Indeed, proteins displaying lower RCS are identifiable in methods with low $\%TP_S$ by their similar profiles in both SILAC channels (red arrows; Supplementary Fig. 31b). For methods with high $\%TP_S$, the most abundant proteins in both SILAC channels display high RCS ($\log_2(S/N) > 0$). During non-SILAC comparisons, free proteins are expected to appear UV-enriched (Supplementary Note 7e), but they can be identified by the similarity of their (free) protein profiles in both UV-crosslinked and non-crosslinked samples (Supplementary Note 8b). Proteins identified exclusively in the UV-crosslinked SILAC channel during SILAC LC-MS/MS analysis of LEAP-RBP and INP fractions were also the least abundant (purple boxes; Supplementary Fig. 31b). While these proteins were given pseudo-$\log_2(S/N)$ ratios of 10 and considered high-confidence RBPs in the traditional sense (purple boxes; Supplementary Fig. 30a, Supplementary Methods), their enrichment is not considered meaningful. For example, XRN1 displays enhanced RNase-sensitivity (S/N) in LEAP-RBP (L) fractions compared to INP (I) fractions by SRA and immunoblot (Fig. 2b). This should equate to a higher S/N ratio for XRN1 in LEAP-RBP fractions as compared to INP fractions by SILAC LC-MS/MS analysis. However, XRN1 was identified in both SILAC channels for LEAP-RBP (high $\%TP_S$) and given a $\log_2(S/N)$ ratio of 3.5, but only detected in the UV-crosslinked SILAC channel for INP (low $\%TP_S$) and given a pseudo-$\log_2(S/N)$ ratio of 10 (XRN1; Supplementary Fig. 30b). This is attributed to improved sensitivity by the LEAP-RBP method and lower limit of detection for noise in the non-crosslinked SILAC channel (Supplementary Note 5). Indeed, proteins only identified in the UV-crosslinked SILAC channel of LEAP-RBP (high $\%TP_S$) fractions were overrepresented by non-RBPs as compared to those identified in INP (low $\%TP_S$) fractions (purple arrows; Supplementary Fig. 31b). Therefore, the inability to detect noise for GO-annotated RBPs is indicative of low sensitivity and $\%TP_S$. Because of this, proteins undetected in the non-crosslinked SILAC channel or non-crosslinked sample were not included in RCS rank analysis because of their less meaningful S/N ratios and lower quantitative accuracy (i.e., # of peptides; Supplementary Methods).

8b. Additional analyses of referenced MS datasets.
Additional analyses of the referenced MS data (**bold**) are provided below. Additional information on MS data processing and analysis of referenced datasets are included in the Supplementary Methods.

XRNAX [5]. Supplementary Data 10, Supplementary Fig. 32a-g.
Analyses were performed with available MS data generated by SILAC LC-MS/MS analysis of 12 XRNAX fractions isolated from pooled UV-crosslinked (0.2 J/cm^2, 254 nm) and non-crosslinked cells (MCF7, HeLa, and HEK293) grown to either half-confluence or confluence and digested for 15 minutes or 30 minutes prior to silica enrichment [5]. In this study, XRNAX fractions were isolated from 30 million UV-crosslinked (0.2 J/cm^2, 254 nm) or non-crosslinked HeLa cells (Supplementary Note 9a). Evaluation of XRNAX fractions by SRA and SYBR Safe (RNA&DNA) staining demonstrated high UV-enrichment of RNA and efficient digestion of DNA (TBE gel analysis or polyacrylamide gel; Fig. 8a, b), consistent with published data ([5]; Fig. 1b). Comparison of XRNAX fractions isolated from UV-crosslinked and non-crosslinked cells showed moderate UV-enrichment of RNase-insensitive (free) protein by SRA and Coomassie Blue (protein) staining (Fig. 8 b). This is consistent with reported observations and rationale for using a SILAC LC-MS/MS approach (Supplementary Note 4b). XRNAX and LEAP-RBP display comparable signal recovery by SRA and immunoblot (e.g., HuR, X vs L; Fig. 8c) but some RBPs display discernibly lower RNase-sensitivity (S/N) in XRNAX fractions (e.g., RPL4). This is consistent with reported observations and rationale for using partial tryptic digestion and repeated TiO_2/SiO_2 enrichment to further enrich RNA-bound peptides prior to LC-MS/MS analysis [5]. Available MS data indicates ~71% of the total protein in XRNAX fractions is RNA-bound ($\%TP_S$) (Source Data Fig. 8e). Comparing the observed RNase-sensitivity of total protein in XRNAX (pre-cleanup) and INP ($\%TP_S = 47$; Source Data Fig. 5h) fractions by SRA and Coomassie Blue (protein) staining (Fig. 2a, 8b) suggests this is highly effective at increasing $\%TP_S$ of XRNAX fractions. Importantly, this can be considered a favorable increase in $\%TP_S$ (Supplementary Methods); i.e., it doesn't discernibly increase non-specific $\%TP_{(S)}$ contributions (2.6; Source Data Fig. 8e). The ability of both RCS and %TP ranking to accurately place GO-annotated RBPs at higher ordinal rank than non-RBPs is considered indicative of methods with high $\%TP_S$ (Supplementary Note 8a). Indeed, compared to non-RBPs, RNase-sensitive RBPs were of mostly higher abundance in XRNAX fractions and scored as higher confidence (Supplementary Fig. 32c, d). A similar trend was observed for all GO-annotated RBPs and non-RBPs (Supplementary Fig. 32f). In

this study, protein quantities were estimated using sum peptide intensities (SPI) rather than iBAQ intensities as done in the original XRNAX study [5]. The 30 most abundant proteins in XRNAX fractions estimated as a percentage of total iBAQ intensity ([5]; Figure S1A) were mostly comparable to the 30 most abundant proteins in XRNAX fractions estimated as a percentage of total SPI (%TP; Supplementary Table 10). This suggests either label-free quantification method is suitable for MS data analyses [3]. Comparison of observed protein quantities in the UV-crosslinked and non-crosslinked SILAC channels shows similar free (low RCS) and dissimilar RNA-bound (high RCS) protein profiles (Supplementary Fig. 32b). These data and observations are reasonably consistent with those reported by the original XRNAX study [5].

OOPs [6]. Supplementary Data 11, 20, Supplementary Fig. 33a-g, 34a-g.
Analyses were performed with available MS data (3 out of 4 replicates) generated by LC-MS/MS analysis of OOPs fractions isolated from the organic phase following AGPC extraction of RNase-treated 5th AGPC interphase samples isolated from UV-crosslinked (0.8 J/cm^2, 254 nm) or non-crosslinked human CD4$^+$ T cells [6]. In this study, OOPs fractions were isolated from the organic phase following AGPC extraction of RNase-treated and untreated 3rd AGPC interphase samples isolated from 30 million UV-crosslinked (0.2 J/cm^2, 254 nm) or non-crosslinked HeLa cells (Supplementary Note 9b). Evaluation of OOPs fractions by SRA and Coomassie Blue (protein) staining demonstrated moderate UV-enrichment of proteins exhibiting ubiquitous RNase-dependent enrichment (OOPs; Fig. 8b). This is consistent with observations of the original OOPs study that 96% of proteins UV-enriched* at the 3rd AGPC interphase (CL), including CL-independent proteins (RNA-free), exhibit RNase-dependent migration into the organic phase ([13]; Fig. 2i, Supplementary Fig. 2h). Therefore, observed RNase-dependent migration of free protein into the organic phase is expected to overestimate *S*/*N* and %TP$_S$ of OOPs fractions by SRA analysis (Fig. 8b, c, Supplementary Note 7e). This is less likely to impact the observed *S*/*N* of RBPs with higher UV-crosslinking efficiencies and/or those more readily enriched by repeated AGPC extraction (Supplementary Note 4e). As noted by the authors of the original OOPs study ([13]; Supplementary Fig. 2b), additional repeated AGPC extraction would be expected to improve UV-dependent enrichment of RNA-bound and free proteins at the AGPC interphase (Fig. 1b, c). Analyses were performed with MS data generated by a non-SILAC LC-MS/MS experiment. Therefore, the observed enrichment efficiencies (*S*/*N*) of RBPs and non-RBPs are expected to reflect their observed UV-enrichment in RNase-treated OOPs fractions by SRA and immunoblot (O, CL vs nCL; Fig 8c). Additionally, the observed abundances (%TP) of RBPs and non-RBPs are expected to mostly reflect their observed abundance in RNase-treated OOPs fractions isolated from UV-crosslinked cells by SRA and immunoblot (O, CL and RNase; Fig. 8c). Available MS data (non-SILAC) showing higher enrichment efficiencies and abundances for RBPs as compared to non-RBPs (O; Fig. 8d, Supplementary Fig. 33a), and high %TP$_S$ (73.4) with high non-specific %TP$_{(S)}$ contributions (24.4) match these predictions (Source Data Fig. 8e). In this study, protein quantities were estimated using sum peptide intensities (SPI) rather than LFQ intensities generated by MaxLFQ as reported in the referenced OOPs study [6]. Comparable analyses on reported LFQ intensities can be found in Supplementary Fig. 34a-g while annotated MS data can be found in Supplementary Data 20. As discussed in Supplementary Note 4f, LFQ algorithms assumes protein profiles are mostly comparable between experimental samples (i.e., CL and nCL). However, protein recovered from UV-crosslinked cells contains both RNA-bound and free protein while protein recovered from non-crosslinked cells contains only free protein; therefore, this LFQ algorithms are expected to underestimate UV-enrichment efficiencies (*S*/*N*; Supplementary Note 8a). Indeed, many proteins showing clear UV-enrichment by SRA and immunoblot appear non-enriched using LFQ intensities (e.g., NCL; Supplementary Fig. 33d, 34d) and %TP$_S$ is discernibly lower (22.1; Supplementary Table 20). In both datasets, similar protein profiles are observed in both UV-crosslinked and non-crosslinked samples (Supplementary Fig. 33b, 34b). This finding is consistent with reported RNase-dependent enrichment of CL independent (RNA free) protein in OOPs fractions [13], and the expectation that free protein quantities display more similar profiles in UV-crosslinked vs non-crosslinked samples (Supplementary Note 8a). Overall, the data and observations presented in this study are consistent with those reported by the original OOPs study [13], and the referenced MS data [6].

Ptex [7]. Supplementary Data 12, 18, 19, Supplementary Fig. 35a-g, 36a-g, 37a-g.
Analyses were performed with available MS data generated by LC-MS/MS analysis of Ptex fractions isolated from UV-crosslinked (1.5 J/cm^2, 254 nm) or non-crosslinked HEK293 cells [7]. In this study, Ptex fractions were isolated from 30 million UV-crosslinked (0.2 J/cm^2, 254 nm) or non-crosslinked HeLa cells

(Supplementary Note 9c). Evaluation of Ptex fractions by SRA and Coomassie Blue (protein) staining demonstrating moderate UV-enrichment of RNase-insensitive protein displaying comparable protein profiles in both UV-crosslinked and non-crosslinked samples (blue boxes, Ptex; Fig. 8b). Indeed, comparing the observed protein quantities in UV-crosslinked and non-crosslinked samples shows highly similar profiles (Supplementary Fig. 35b). Additionally, the observed profile of total protein in Ptex fractions by SDS-PAGE and Silver Stain (RNA, DNA, and potein) staining (Silver Stain, Ptex; Fig. 8b) is consistent with reported polyacrylamide gels showing SDS-PAGE separated Ptex fractions stained with Silver Stain ([7]; Supplementary Figure 22). Evaluation of Ptex fractions by SRA and immunoblot showed UV-enrichment of RNase-insensitive RBPs (e.g., NCL and TRAPα) and GRP94 (P; Fig. 8c). This is consistent with published immunoblots showing similar non-specific UV-enrichment of RNase-insensitive proteins in Ptex fractions ([7]; Fig. 1f). Analyses were performed with MS data generated by a non-SILAC LC-MS/MS experiment. Therefore, the observed enrichment efficiencies (*S*/*N*) of RBPs and non-RBPs are expected to reflect their observed UV-enrichment in RNase-treated Ptex fractions by SRA and immunoblot (P, CL vs nCL; Fig 8c). Additionally, the observed abundances (%TP) of RBPs and non-RBPs are expected to mostly reflect their observed abundances in RNase-treated Ptex fractions isolated from UV-crosslinked cells by SRA and immunoblot (P, CL and RNase; Fig. 8c). Available MS data (non-SILAC) showing non-specific *S*/*N* (red vs blue solid line; Supplementary Fig. 35a), and non-specific $\%TP_{(S)}$ contributions (28.4) match these predictions (Source Data Fig. 8e). The observed abundance of proteins (e.g., NCL and GRP94) in Ptex fractions by SRA and immunoblot are consistent with their observed abundance in available MS data (Supplementary Fig. 35d). Comparable analyses of MS data generated by LC-MS/MS analysis of Ptex fractions isolated from HEK293 cells UV-irradiated with 0.015 or 0.15 J/cm^2 (254 nm) were included in Supplementary Fig. 36a-g, Supplementary Fig. 37a-g respectively; annotated MS data can be found in Supplementary Table 18, Supplementary Table 19 respectively. In this study, protein quantities were estimated using sum peptide intensities (SPI) rather than iBAQ intensities as done in the original Ptex study [7]. Nonetheless, published scatterplots showing protein abundances estimated using iBAQ intensities are comparable to protein abundances estimated using SPI values ([7]; Fig. 5f). These data and observations are reasonably consistent with those reported by the original Ptex study [7].

TRAPP [4]. Supplementary Data 13, 16, 17; Supplementary Fig. 38a-j, 39a-g, 40a-g.

Analyses were performed with available MS data generated by SILAC LC-MS/MS analysis of TRAPP fractions isolated from pooled samples containing non-crosslinked and UV-crosslinked (400, 800, or 1360 mJ/cm^2; 254 nm) yeast cells [4]. In this study, TRAPP fractions were isolated from 30 million UV-crosslinked (0.2 J/cm^2, 254 nm) or non-crosslinked HeLa cells (Supplementary Note 9d). Evaluation of TRAPP fractions (CL vs nCL) by SRA and Coomassie Blue (protein) staining demonstrated high UV-dependent enrichment of RNase-sensitive proteins (blue boxes, TRAPP; Fig. 8b). Further evaluation by SRA and immunoblot showed high specificity for RNase-sensitive RBPs and signal-dependent recovery of noise (TRAPP, gold box and red (signal) or blue (noise) arrows; Source Data Fig. 8c). The observed RNase-sensitivity of total protein in TRAPP fractions (CL) by SRA and Coomassie Blue (protein) staining is suggestive of high $\%TP_S$ (Fig. 9a, Supplementary Note 8a). Indeed, analysis of available SILAC LC-MS/MS data (1360 mJ/cm^2, 254 nm) indicates 86% of the total protein in the sample is RNA-bound ($\%TP_{(S)}$; Source Data Fig. 9c). The observed $\%TP_S$ of TRAPP fractions was found to depend on the UV-dose, with only 60% or 67% of total protein originating from RNA-bound protein when yeast cells were UV-irradiated with 400 and 800 mJ/cm^2 (254 nm) respectively (Source Data Fig. 9j). The apparent non-specific $\%TP_{(S)}$ of TRAPP fractions (400, 800, and 1360) is due to incomplete GO-annotation (GO:RBP) of ribosomal proteins (Source Data Fig. 9j). When accounting for ribosomal proteins lacking RBP-annotations (i.e., proteins whose primary gene name starts with “RPS” or “RPL”), non-specific $\%TP_{(S)}$ contributions are greatly reduced (Supplementary Fig. 38i). Curiously, there is a larger improvement for TRAPP 400 (95) and TRAPP 800 (95) fractions as compared to TRAPP 1360 (79) fractions despite their lower $\%TP_S$ (60, 67, and 86 respectively; Supplementary Fig. 38h, i, Supplementary Data 13, 16, 17). Because RBPs contributed most of the total RNA-bound protein in XRNAX ($\%TP_{(S),\ RBPs}$ = 97), LEAP-RBP ($\%TP_{(S),\ RBPs}$ = 98), and INP ($\%TP_{(S),\ RBPs}$ = 99) fractions by SILAC LC-MS/MS (Source Data Fig. 5h, 8e), this likely reflect a UV-dependent decrease in $\%TP_{(S)}$ specificity (Supplementary Fig. 38h, i). To test this, we binned 30 most abundant RNA-bound non-RBPs ($\%TP_S$ rank) identified in TRAPP 1360 fractions and compared their estimated $\%TP_S$ and $\%TP_N$ contributions in TRAPP 400, 800, and 1360 fractions. Indeed, there was a clear UV-dose dependent decrease in $\%TP_S$ contributions of most ribosomal protein (red

text) and an increase in %TP$_S$ contributions for all non-ribosomal proteins (blue text) (Supplementary Fig. 38j). We speculate high levels of UV-crosslinking is resulting in more non-specific UV-crosslinking and/or RBP-specific signal loss during the stringent purification procedures. In support of this, we observed a decreased in RNA yield in TRAPP fractions isolated from UV-crosslinked vs non-crosslinked cells (µg of RNA, CL vs nCL; Fig. 8b). The effect of UV-dose on RNA yield was not evaluated in the original TRAPP study [4]. Comparison of TRAPP and LEAP-RBP fractions isolated from UV-crosslinked cells by SRA and immunoblot showed comparable recovery of ribosomal proteins (e.g., RPL4 and RPL8) displaying high RNase-sensitivity (*S*/*N*), but a large difference in recovery of non-ribosomal proteins (Fig. 9b). Indeed, available MS data shows RPL4 and RPL8, among other ribosomal proteins, as the most abundant RBPs in TRAPP (Source Data Fig. 9h, Supplementary Fig. 38d). These observations illustrate how variations in signal recovery can introduce potential biases (Supplementary Note 1). These data and observations are reasonably consistent with those reported by the original TRAPP study [4].

RIC and eRIC [8]. Supplementary Data 14, 15; Supplementary Fig. 41a-h, 42a-g.
Analyses were performed with available MS data ([8]; full dataset tab of Supplementary Data 1), generated by LC-MS/MS analysis of RIC and eRIC fractions isolated from UV-crosslinked (0.15 J/cm^2, 254 nm) or non-crosslinked Jurkat cells. In this study, RIC fractions were isolated from 30 million UV-crosslinked (0.2 J/cm^2, 254 nm) or non-crosslinked HeLa cells (Supplementary Note 9e). Evaluation of RIC fractions (CL vs nCL) by SRA and Coomassie Blue (protein) staining demonstrated high UV-enrichment of RNase-sensitive proteins (blue boxes, RIC; Fig. 8b). Further evaluation by SRA and immunoblot showed high specificity for RNase-sensitive RBPs and signal-dependent recovery of noise (RIC, gold box and red (signal) or blue (noise) arrows; Source Data Fig. 8c). *S*/*N*-based analyses of RIC fractions by SRA and SILAC LC-MS/MS analysis have not yet been reported. However, the RNase-sensitivity of total protein in RIC fractions (CL) by SRA and Coomassie Blue (protein) staining is indicative of high %TP$_S$ (Fig. 9a, b, Supplementary Note 8a). This is expected to limit UV-enriched free protein contributions towards observed enrichment efficiencies (*S*/*N*) and RNA-bound abundances (%TP$_S$) of RBPs and non-RBPs (Supplementary Note 7e). Nonetheless, the observed enrichment efficiencies (*S*/*N*) of RBPs and non-RBPs in available MS data are expected to reflect their observed UV-enrichment in RNase-treated RIC fractions by SRA and immunoblot (R, + CL vs nCL; Fig 8c). Additionally, the abundances (%TP) of RBPs and non-RBPs are expected to mostly reflect their observed abundances in RNase-treated RIC fractions isolated from UV-crosslinked cells by SRA and immunoblot (R, CL and RNase; **Fig. 5c**). Available MS data (non-SILAC) showing high enrichment efficiencies and abundances for most RBPs over non-RBPs match these predictions (R; Fig. 8d, Supplementary Fig. 41d). Most of the proteins identified in RIC fractions by LC-MS/MS have prior RBP-annotations (GO:RBP) regardless of their individual enrichment efficiencies (*S*/*N*; Supplementary Fig. 341a). We postulated that RNA-binding activity for many of the GO-annotated RBPs displaying low *S*/*N* ratios was inferred because of their UV-enrichment* status in prior RIC studies (non-SILAC). Indeed, the RNA-binding activity for 87 of the bottom 100 RBPs ranked by RCS and identified in the referenced RIC study was inferred by their UV-enrichment* status in prior RIC studies (Supplementary Fig. 41h) [24, 41]. As demonstrated in this study, UV-enrichment* alone is insufficient evidence for assigning RNA-binding activity. Therefore, the accuracy of RBP-annotations (GO:RBP) would likely benefit from additional criteria such as demonstrating their RNase-sensitivity by SRA and immunblot. These observations suggest the number of RNA-binding proteins has been significantly overestimated (Supplementary Note 7c, d). The 300 most abundant proteins identified in LEAP-RBP fractions by SILAC LC-MS/MS contribute 95% of total RNA-bound protein in the sample (%TP$_{(S)}$). Therefore, it's unlikely that a decrease in RBP-annotations (GO:RBP) would diminish the ability of %TP$_S$ to reflect method specificity.

Compared to the TRAPP method, RIC displayed more efficient recovery of non-ribosomal proteins (e.g., HuR, pAbPC1, and PABPC4) and less efficient recovery of ribosomal protein (e.g., RPL4 and RPL8) by SRA and immunoblot (RNase T vs R; Fig. 9b). However, when compared to LEAP-RBP, the selective recovery of non-ribosomal proteins by RIC is is no longer apparent (RNase, R vs L; Fig. 9b). Indeed, the abundance of ribosomal (e.g., RPL4 and RPL8) relative to non-ribosomal proteins (e.g., HuR, pAbPC1, and PABPC4) in RIC fractions is comparable to LEAP-RBP fractions (Supplementary Fig. 30b, 41d). The high abundance of NCL relative to other proteins (~one order of magnitude) identified RIC fractions by LC-MS/MS analysis is consistent with its observed abundance (~110 kD band) by SRA and Coomassie Blue (protein) staining

(RNase, R; Fig. 9a). The abundance of ribosomal proteins identified in RIC fractions by LC-MS/MS is consistent with TBE gel analysis of RIC fractions (CL) showing appreciable recovery of rRNA (RNP fraction, RIC; Fig. 8a). Indeed, these observations were reported by others and served as the driving force behind development of more stringent protocols utilizing LNA probes (eRIC) ([8]; Fig. 2a). Compared to RIC (Supplementary Fig. 41d), the abundance of ribosomal proteins (e.g., RPL4 and RPL8) identified in eRIC fractions by LC-MS/MS are decreased relative to non-ribosomal proteins (e.g., HuR and TIA1) (Supplementary Fig. 42d). This includes the highly abundant nucleolar RBP, nucleolin (NCL), consistent with published polyacrylamide gels stained by Silver Stain showing a decrease in observable NCL migrating at its expected weight (~110 kD) in eRIC fractions (RNase) when compared to RIC fractions (RNase) ([8]; Fig. 2c). These data and observations are reasonably consistent with those reported by the referenced RIC and eRIC study [8].

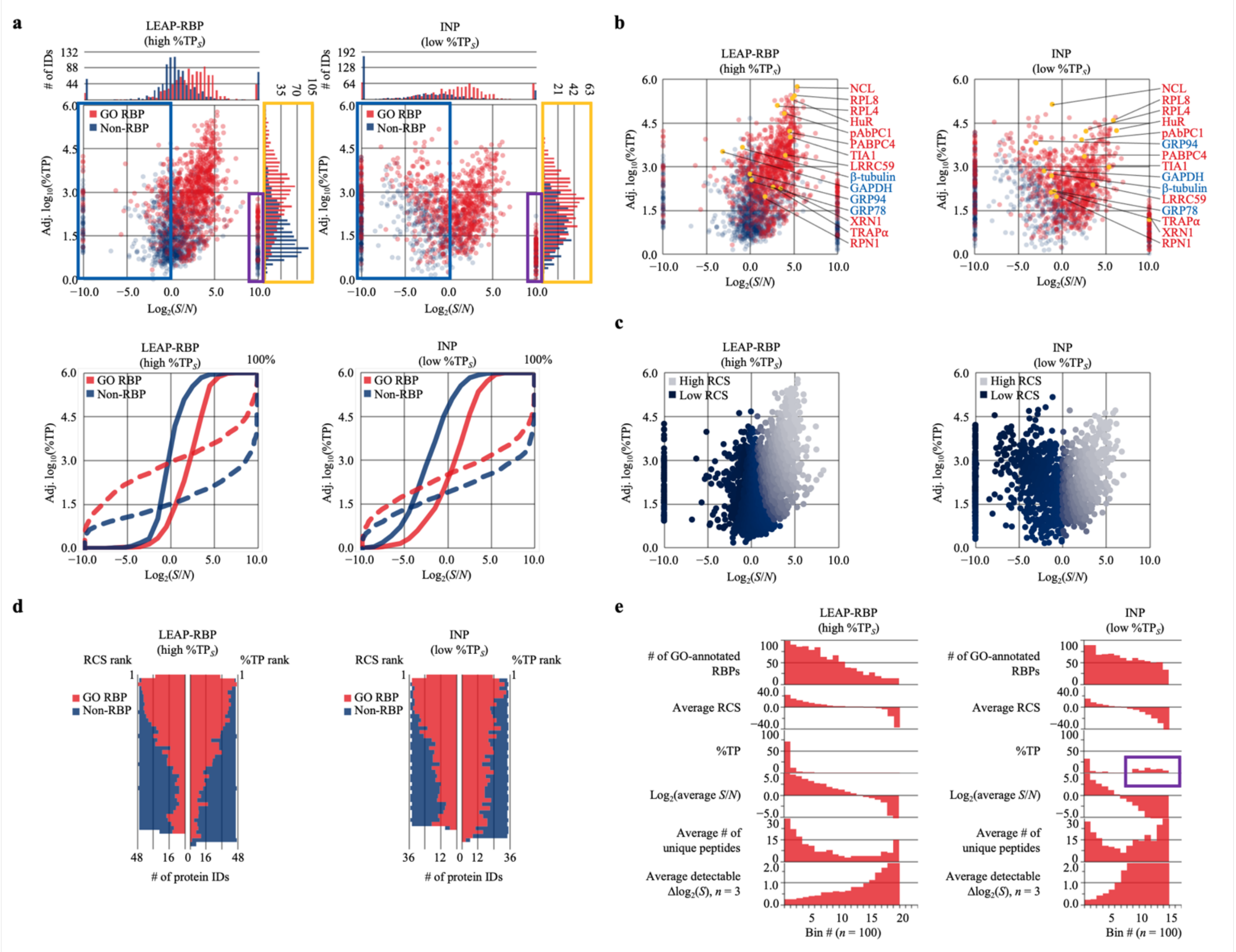

Supplementary Figure 30: SILAC LC-MS/MS analysis distinguishes methods with high or low %TP$_S$ part 1. a-e Analyses were performed with MS data from SILAC LC-MS/MS analysis of LEAP-RBP and INP fractions. **a** Scatterplots illustrating the difference in relative abundance ($\Delta\log_{10}$(%TP)) of RBPs as compared to non-RBPs (gold boxes) for methods with high or low %TP$_S$; plotted as a function of their $\log_2(S/N)$ ratios to evaluate RNA-bound and free protein contributions towards observed abundances (blue boxes). Corresponding frequency curves plotted as a function of $\log_2(S/N)$ (solid) or $\log_{10}$(%TP) (dashed). **b** Main scatterplots from **a** with labeled data points representing proteins evaluated by SRA and immunoblot in Fig. 2b. Color of text determined by SRA and immunoblot analysis of total clRNP fractions; red text: RNase-sensitive RBP; blue text: undetected or RNase-insensitive protein. **c** Main scatterplots from **a** with color overlay based on RCS rank; the most abundant proteins identified in RNP fractions with high %TP$_S$ are more representative of their RNA-bound abundances (positive RCS). **d** The ability of RCS and %TP ranking to accurately place RBPs higher than non-RBPs is indicative of methods with high %TP$_S$. **e** RCS rank analysis allows comprehensive evaluation of method specificity and suitability for RBP studies: # of RBPs displaying positive RCS, coverage (# of peptides), and average detectable $\Delta\log_2(S)$. Proteins more representative of free quantities ($S/N < 0$) are automatically placed at lower ordinal rank (RCS) and identified by their high %TP contributions (purple box, INP), and coverage (# of peptides). Source data are provided as a Source Data file.

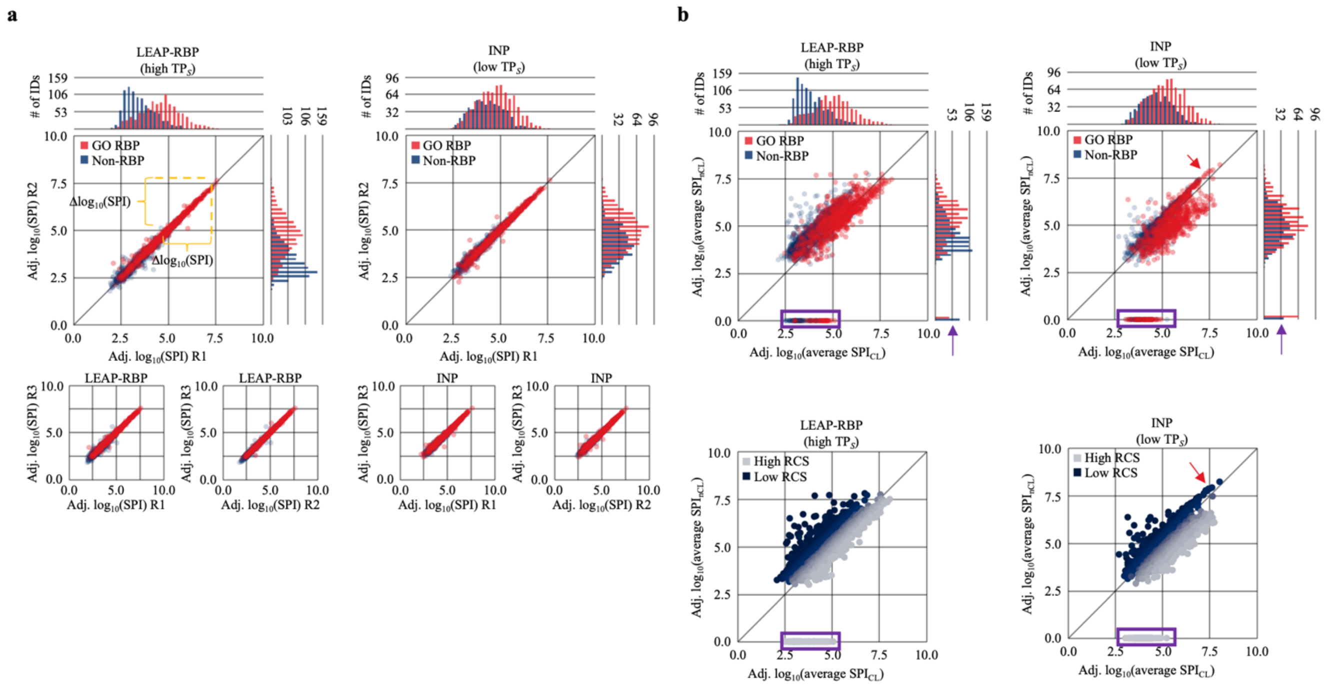

Supplementary Figure 31: SILAC LC-MS/MS analysis distinguishes methods with high or low %TP_S part 2. a, **b** Analyses were performed with MS data from SILAC LC-MS/MS analysis of LEAP-RBP and INP fractions. **a** Comparison of observed proteins quantities (SPI) between biological replicates normalized to total SPI illustrates the expected correlation for proteomic samples with comparable protein profiles (i.e., relative quantities of proteins). **b** Comparisons of observed protein quantities in the UV-crosslinked and non-crosslinked SILAC channels after mean-normalization of total SPI (total SPI_{CL} = total SPI_{nCL}); color overlays based on GO-annotation status (red: RBP; blue: non-RBP) or ordinal RCS rank (silver: high RCS; navy blue: low RCS). Source data are provided as a Source Data file.

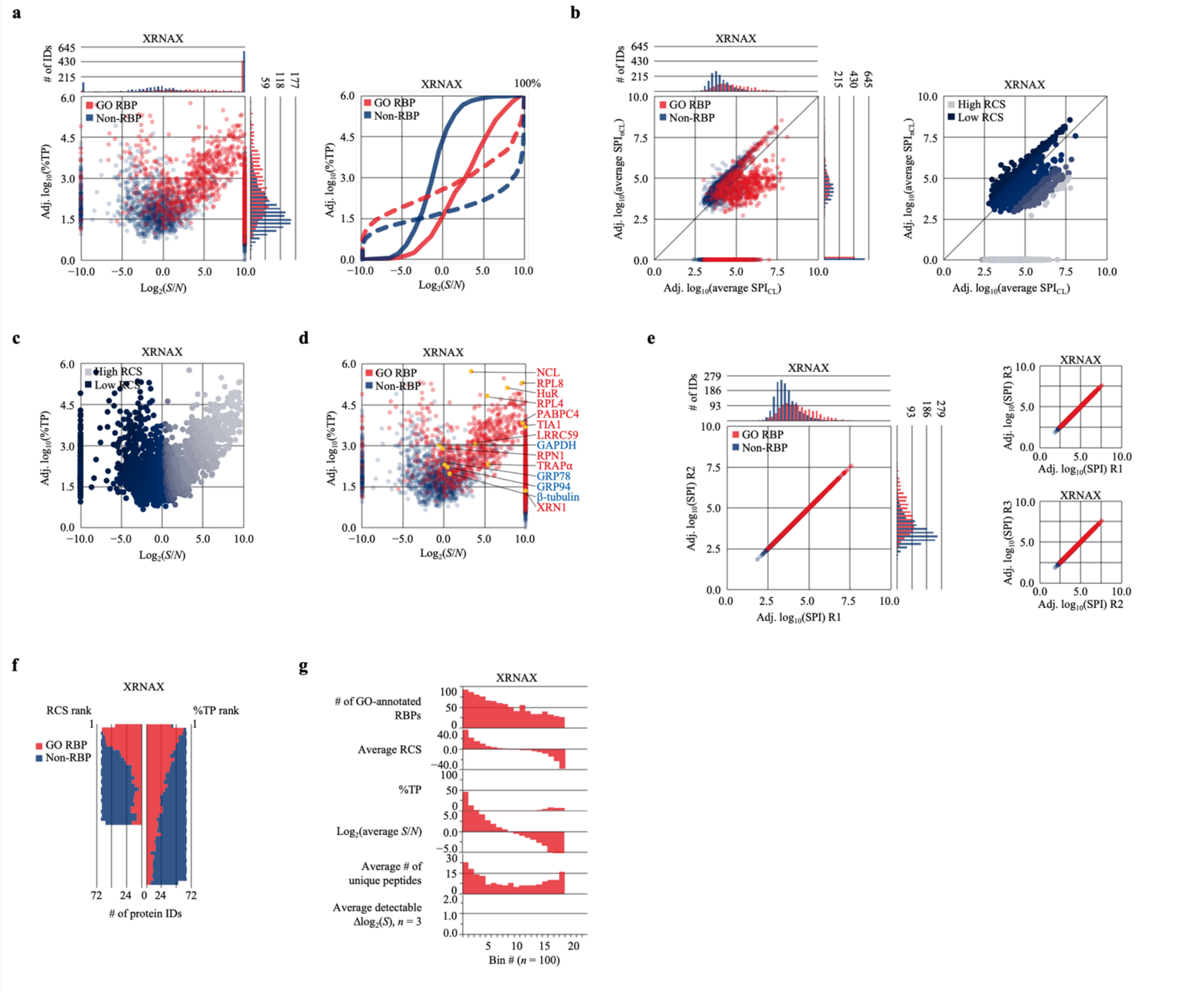

Supplementary Figure 32: Extended analysis of available MS data (SILAC) from referenced XRNAX study [5]. The following analyses were performed on the MS dataset provided in Supplementary Data 10. **a** Method specificity for RNA-bound RBPs evaluated by comparing the abundance (%TP) of RBPs and non-RBPs as a function of their $\log_2(S/N)$ ratios. Corresponding frequency curves plotted as a function of $\log_2(S/N)$ (solid) or $\log_{10}$(%TP) (dashed). **b** Comparison of observed protein quantities in the UV-crosslinked and non-crosslinked SILAC channel after mean-normalization of total SPI (total SPI_{CL} = total SPI_{nCL}); color overlays based on GO-annotation status (red: RBP; blue: non-RBP) or ordinal RCS rank (silver: high RCS; navy blue: low RCS). **c** Main scatterplot from **a** with color overlay based on ordinal RCS rank. **d** Main scatterplot from **a** with labeled data points representing proteins evaluated by SRA and immunoblot in Fig. 8c. Color of text based on conclusions from SRA and immunoblot analysis of total clRNP fractions; red text: RNase-sensitive RBP; blue text: undetected or RNase-insensitive protein. **e** Comparison of observed protein quantities between replicate sample. Because available MS data for XRNAX does not include replicate samples, SPI_{CL} and SPI_{nCL} values represent the average non-zero values of 12 different samples (Methods). **f** Comparison of RBP and non-RBP placement by RCS and %TP ranking. **g** RCS rank analysis. Source data are provided as a Source Data file.

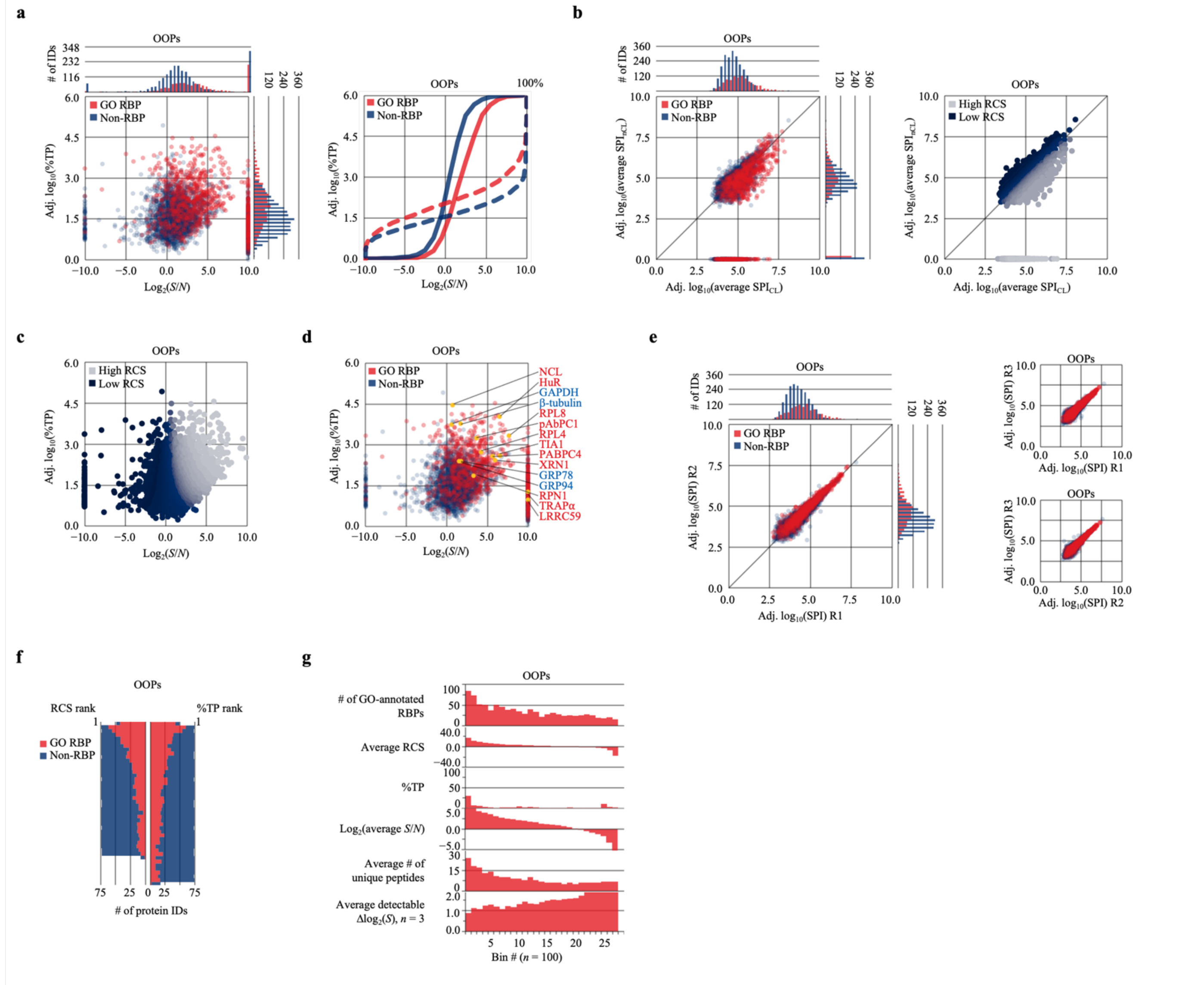

Supplementary Figure 33: Extended analysis of available MS data (non-SILAC) from referenced OOPs study [6]; not from original OOPs methods paper [13]. The following analyses were performed on the MS dataset provided in Supplementary Data 11. **a** Method specificity for RNA-bound RBPs evaluated by comparing the abundance (%TP) of RBPs and non-RBPs as a function of their $\log_2(S/N)$ ratios. Corresponding frequency curves plotted as a function of $\log_2(S/N)$ (solid) or $\log_{10}$(%TP) (dashed). **b** Comparison of observed protein quantities in UV-crosslinked and non-crosslinked samples after mean-normalization of total SPI (total SPI_{CL} = total SPI_{nCL}); color overlays based on GO-annotation status (red: RBP; blue: non-RBP) or ordinal RCS rank (silver: high RCS; navy blue: low RCS). **c** Main scatterplot from **a** with color overlay based on ordinal RCS rank. **d** Main scatterplot from **a** with labeled data points representing proteins evaluated by SRA and immunoblot in Fig. 8c. Color of text based on conclusions from SRA and immunoblot analysis of total clRNP fractions; red text: RNase-sensitive RBP; blue text: undetected or RNase-insensitive protein. **e** Comparison of observed protein quantities between replicate sample. **f** Comparison of RBP and non-RBP placement by RCS and %TP ranking. **g** RCS rank analysis. Source data are provided as a Source Data file.

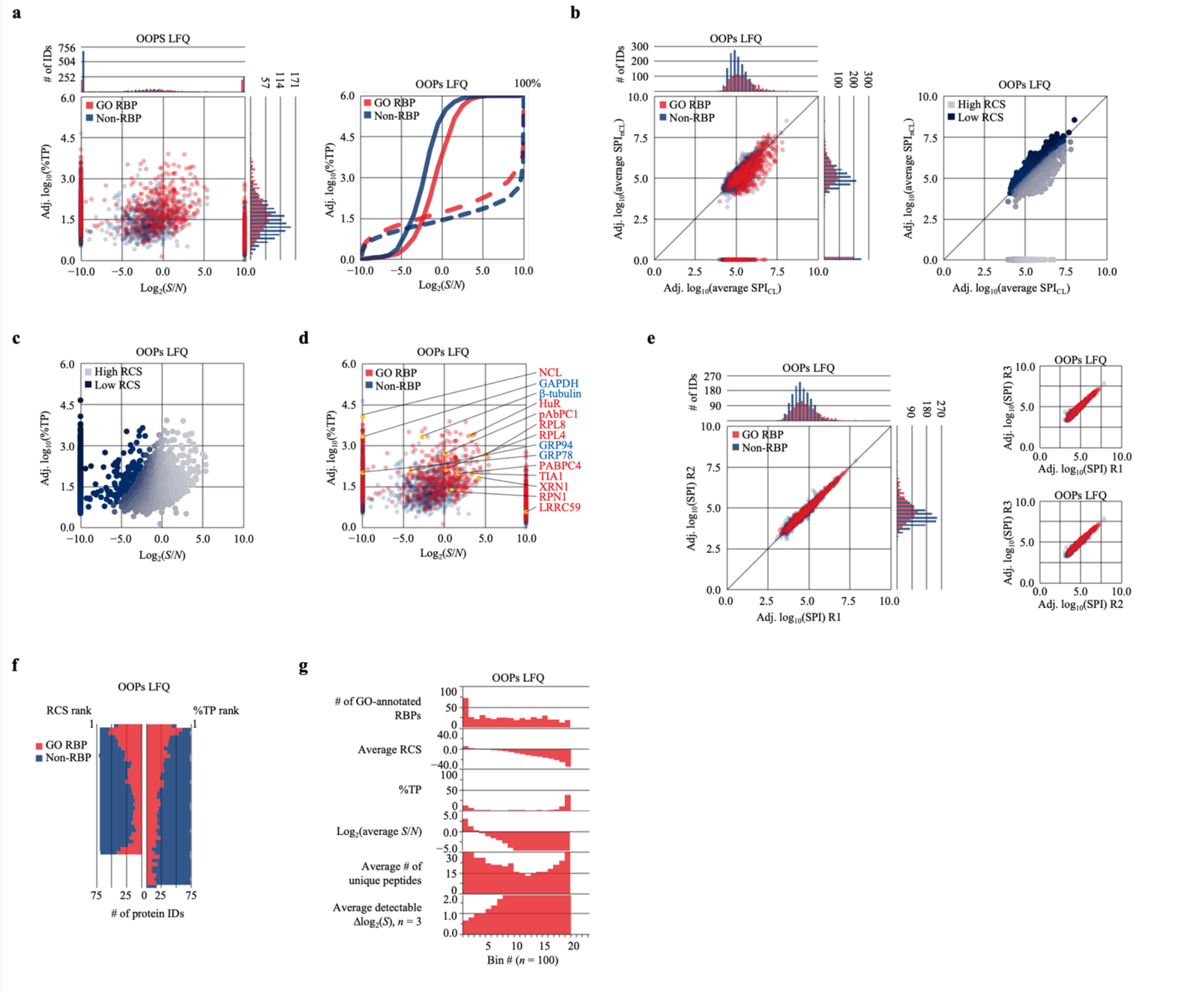

Supplementary Figure 34: Extended analysis of LFQ data (non-SILAC) provided by referenced OOPs study [6]; not from original OOPs methods paper [13]. The following analyses were performed on the MS dataset provided in Supplementary Data 20. **a** Method specificity for RNA-bound RBPs evaluated by comparing the abundance (%TP) of RBPs and non-RBPs as a function of their $\log_2(S/N)$ ratios. Corresponding frequency curves plotted as a function of $\log_2(S/N)$ (solid) or $\log_{10}$(%TP) (dashed). **b** Comparison of observed protein quantities in UV-crosslinked and non-crosslinked samples after mean-normalization of total SPI (total SPI_{CL} = total SPI_{nCL}); color overlays based on GO-annotation status (red: RBP; blue: non-RBP) or ordinal RCS rank (silver: high RCS; navy blue: low RCS). **c** Main scatterplot from **a** with color overlay based on ordinal RCS rank. **d** Main scatterplot from **a** with labeled data points representing proteins evaluated by SRA and immunoblot in Fig. 8c. Color of text based on conclusions from SRA and immunoblot analysis of total clRNP fractions; red text: RNase-sensitive RBP; blue text: undetected or RNase-insensitive protein. **e** Comparison of observed protein quantities between replicate sample. **f** Comparison of RBP and non-RBP placement by RCS and %TP ranking. **g** RCS rank analysis. Source data are provided as a Source Data file.

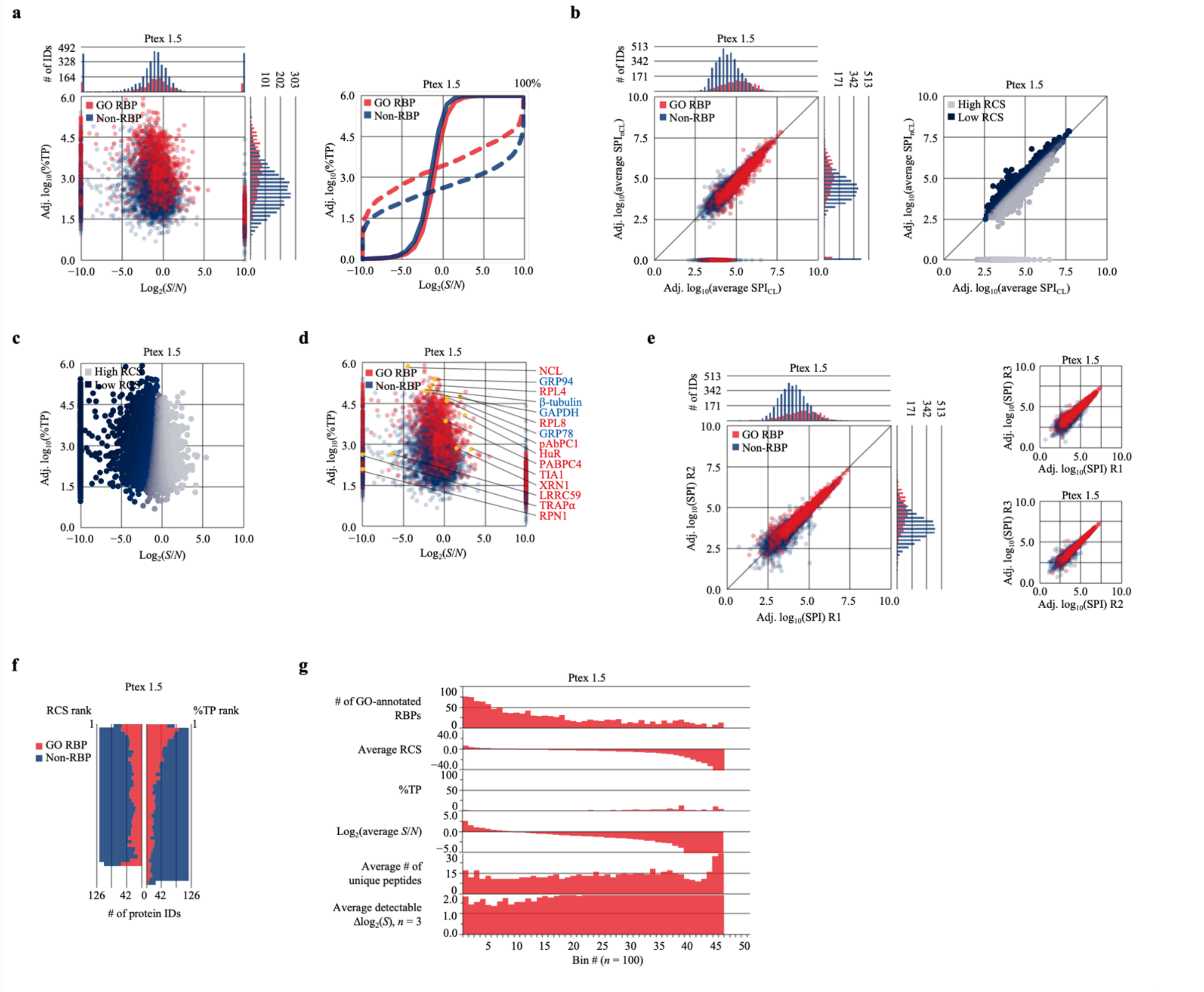

Supplementary Figure 35: Extended analysis of available MS data (non-SILAC) from referenced Ptex study [7]. Ptex 1.5: Ptex fractions isolated from HEK293 cells UV-irradiated with 1.5 J/cm^2 (254 nm). The following analyses were performed on the MS dataset provided in Supplementary Data 12. **a** Method specificity for RNA-bound RBPs evaluated by comparing the abundance (%TP) of RBPs and non-RBPs as a function of their $\log_2(S/N)$ ratios. Corresponding frequency curves plotted as a function of $\log_2(S/N)$ (solid) or $\log_{10}$(%TP) (dashed). **b** Comparison of observed protein quantities in UV-crosslinked and non-crosslinked samples after mean-normalization of total SPI (total SPI_{CL} = total SPI_{nCL}); color overlays based on GO-annotation status (red: RBP; blue: non-RBP) or ordinal RCS rank (silver: high RCS; navy blue: low RCS). **c** Main scatterplot from **a** with color overlay based on ordinal RCS rank. **d** Main scatterplot from **a** with labeled data points representing proteins evaluated by SRA and immunoblot in Fig. 8c. Color of text based on conclusions from SRA and immunoblot analysis of total clRNP fractions; red text: RNase-sensitive RBP; blue text: undetected or RNase-insensitive protein. **e** Comparison of observed protein quantities between replicate sample. **f** Comparison of RBP and non-RBP placement by RCS and %TP ranking. **g** RCS rank analysis. Source data are provided as a Source Data file.

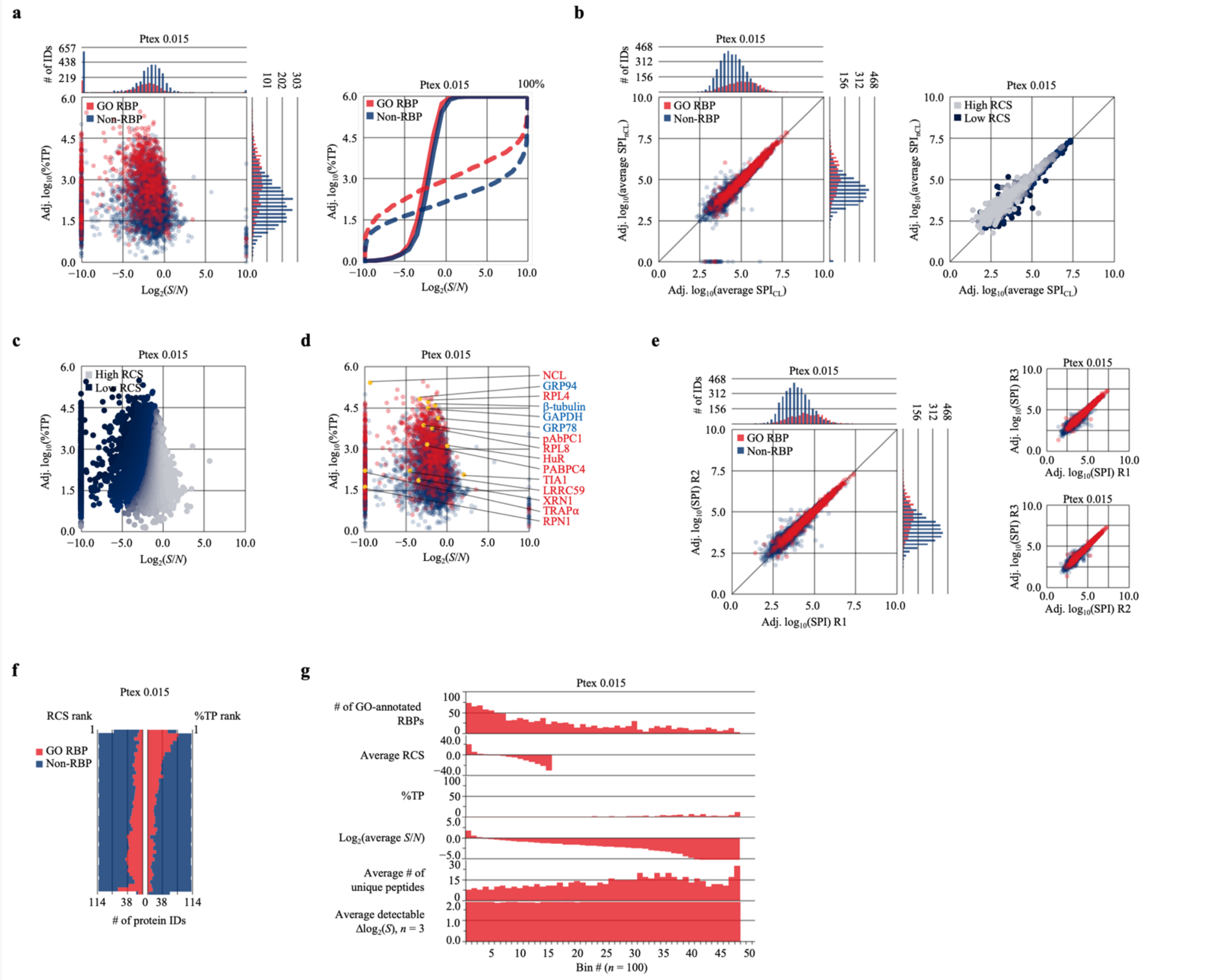

Supplementary Figure 36: Extended analysis of available MS data (non-SILAC) from referenced Ptex study [7]. Ptex 0.015: Ptex fractions isolated from HEK293 cells UV-irradiated with 0.015 J/cm^2 (254 nm). The following analyses were performed on the MS dataset provided in Supplementary Data 18. **a** Method specificity for RNA-bound RBPs evaluated by comparing the abundance (%TP) of RBPs and non-RBPs as a function of their $\log_2(S/N)$ ratios. Corresponding frequency curves plotted as a function of $\log_2(S/N)$ (solid) or $\log_{10}$(%TP) (dashed). **b** Comparison of observed protein quantities in UV-crosslinked and non-crosslinked samples after mean-normalization of total SPI (total SPI_{CL} = total SPI_{nCL}); color overlays based on GO-annotation status (red: RBP; blue: non-RBP) or ordinal RCS rank (silver: high RCS; navy blue: low RCS). **c** Main scatterplot from **a** with color overlay based on ordinal RCS rank. **d** Main scatterplot from **a** with labeled data points representing proteins evaluated by SRA and immunoblot in Fig. 8c. Color of text based on conclusions from SRA and immunoblot analysis of total clRNP fractions; red text: RNase-sensitive RBP; blue text: undetected or RNase-insensitive protein. **e** Comparison of observed protein quantities between replicate sample. **f** Comparison of RBP and non-RBP placement by RCS and %TP ranking. **g** RCS rank analysis. Source data are provided as a Source Data file.

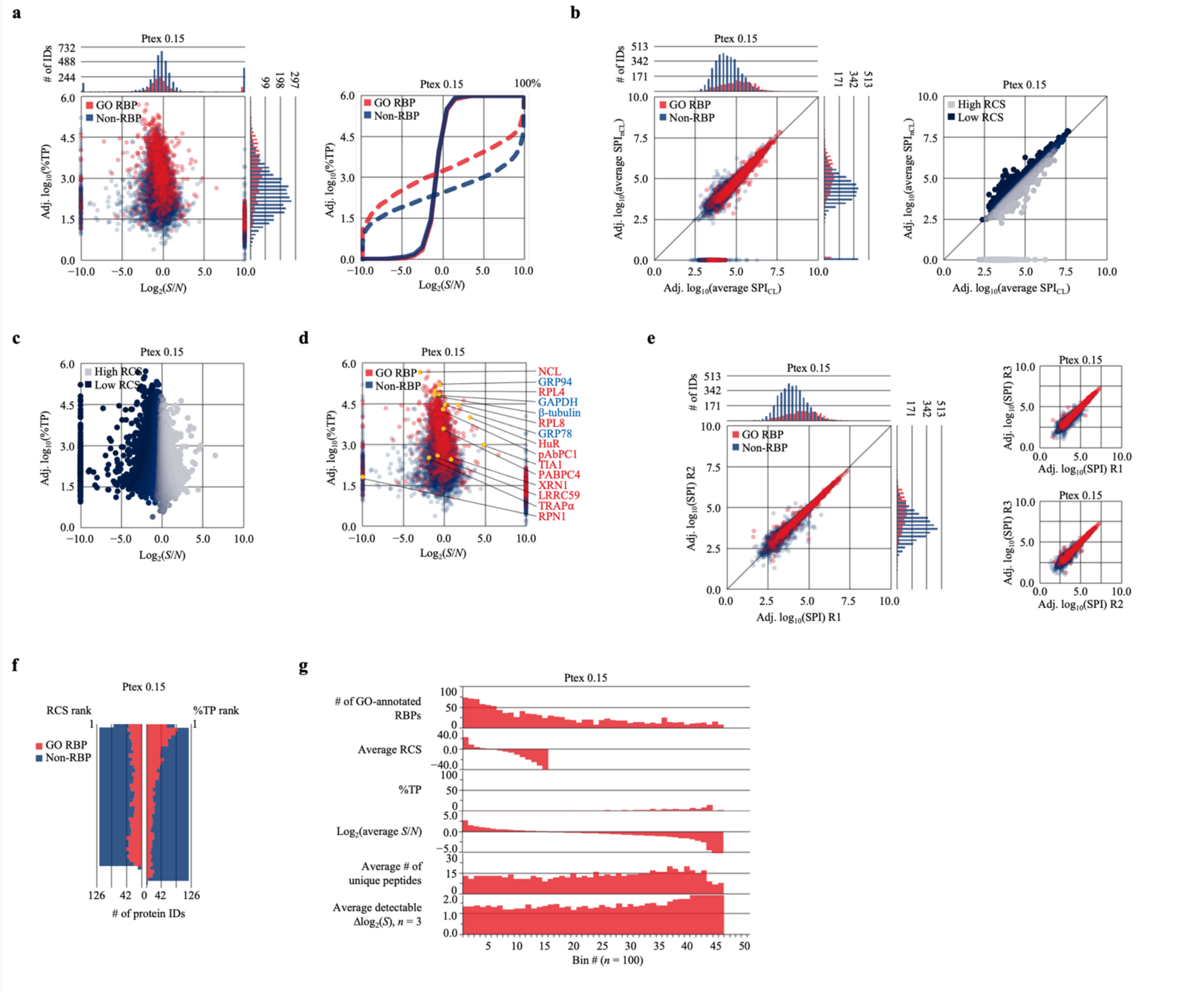

Supplementary Figure 37: Extended analysis of available MS data (non-SILAC) from referenced Ptex study [7]. Ptex 0.15: Ptex fractions isolated from HEK293 cells UV-irradiated with 0.15 J/cm^2 (254 nm). The following analyses were performed on the MS dataset provided in Supplementary Data 19. **a** Method specificity for RNA-bound RBPs evaluated by comparing the abundance (%TP) of RBPs and non-RBPs as a function of their $\log_2(S/N)$ ratios. Corresponding frequency curves plotted as a function of $\log_2(S/N)$ (solid) or $\log_{10}$(%TP) (dashed). **b** Comparison of observed protein quantities in UV-crosslinked and non-crosslinked samples after mean-normalization of total SPI (total SPI_{CL} = total SPI_{nCL}); color overlays based on GO-annotation status (red: RBP; blue: non-RBP) or ordinal RCS rank (silver: high RCS; navy blue: low RCS). **c** Main scatterplot from **a** with color overlay based on ordinal RCS rank. **d** Main scatterplot from **a** with labeled data points representing proteins evaluated by SRA and immunoblot in Fig. 8c. Color of text based on conclusions from SRA and immunoblot analysis of total clRNP fractions; red text: RNase-sensitive RBP; blue text: undetected or RNase-insensitive protein. **e** Comparison of observed protein quantities between replicate sample. **f** Comparison of RBP and non-RBP placement by RCS and %TP ranking. **g** RCS rank analysis. Source data are provided as a Source Data file.

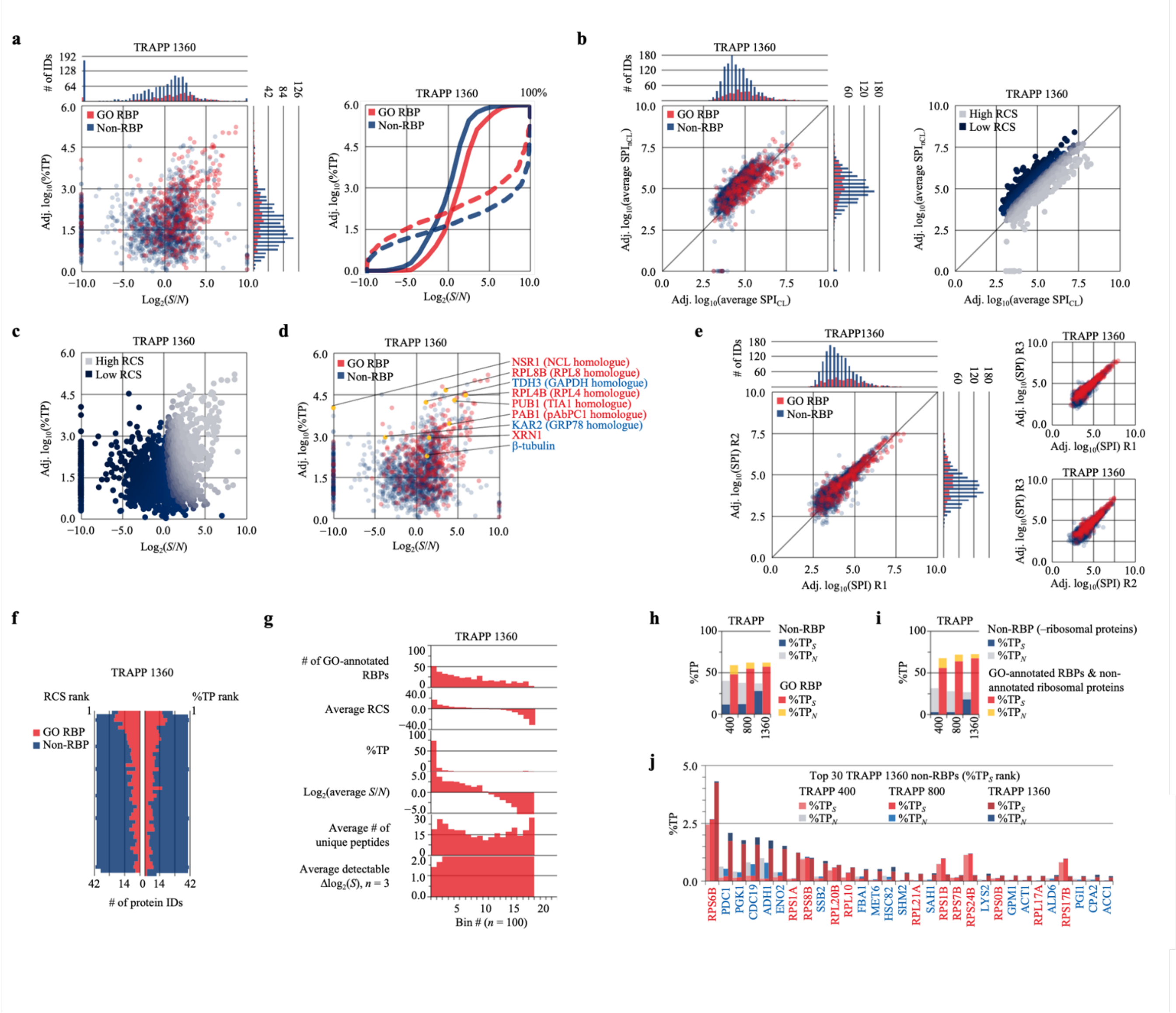

Supplementary Figure 38: Extended analysis of available MS data (SILAC) from referenced TRAPP study [4]. The following analyses were performed on the MS dataset provided in Supplementary Data 13. Notably, the referenced TRAPP study was performed in yeast and may limit comparison due to incomplete GO-annotations. **a** Method specificity for RNA-bound RBPs evaluated by comparing the abundance (%TP) of RBPs and non-RBPs as a function of their $\log_2(S/N)$ ratios. Corresponding frequency curves plotted as a function of $\log_2(S/N)$ (solid) or $\log_{10}$(%TP) (dashed). **b** Comparison of observed protein quantities in the UV-crosslinked and non-crosslinked SILAC channel after mean-normalization of total SPI (total SPI_{CL} = total SPI_{nCL}); color overlays based on GO-annotation status (red: RBP; blue: non-RBP) or ordinal RCS rank (silver: high RCS; navy blue: low RCS). **c** Main scatterplot from **a** with color overlay based on ordinal RCS rank. **d** Main scatterplot from **a** with labeled data points representing homologues of proteins evaluated by SRA and immunoblot in Fig. 9b. Color of text based on conclusions from SRA and immunoblot analysis of total clRNP fractions; red text: RNase-sensitive RBP; blue text: undetected or RNase-insensitive protein. **e** Comparison of observed protein quantities between replicate sample. **f** Comparison of RBP and non-RBP placement by RCS and %TP ranking. **g** RCS rank analysis. **h** Stacked bar charts showing the estimated $\%TP_S$ and $\%TP_N$ contributions of RBPs and non-RBPs. **i** Stacked bar charts from **h** but RBPs include ribosomal proteins lacking GO-annotations in yeast (see Supplementary Data 13, 16, 17). **j** Estimated $\%TP_S$ and $\%TP_N$ contributions of the top 30 TRAPP 1360 non-RBPs ($\%TP_S$ rank) in TRAPP 400, 800, and 1360 fractions; red text: ribosomal protein, blue text: other. Source data are provided as a Source Data file.

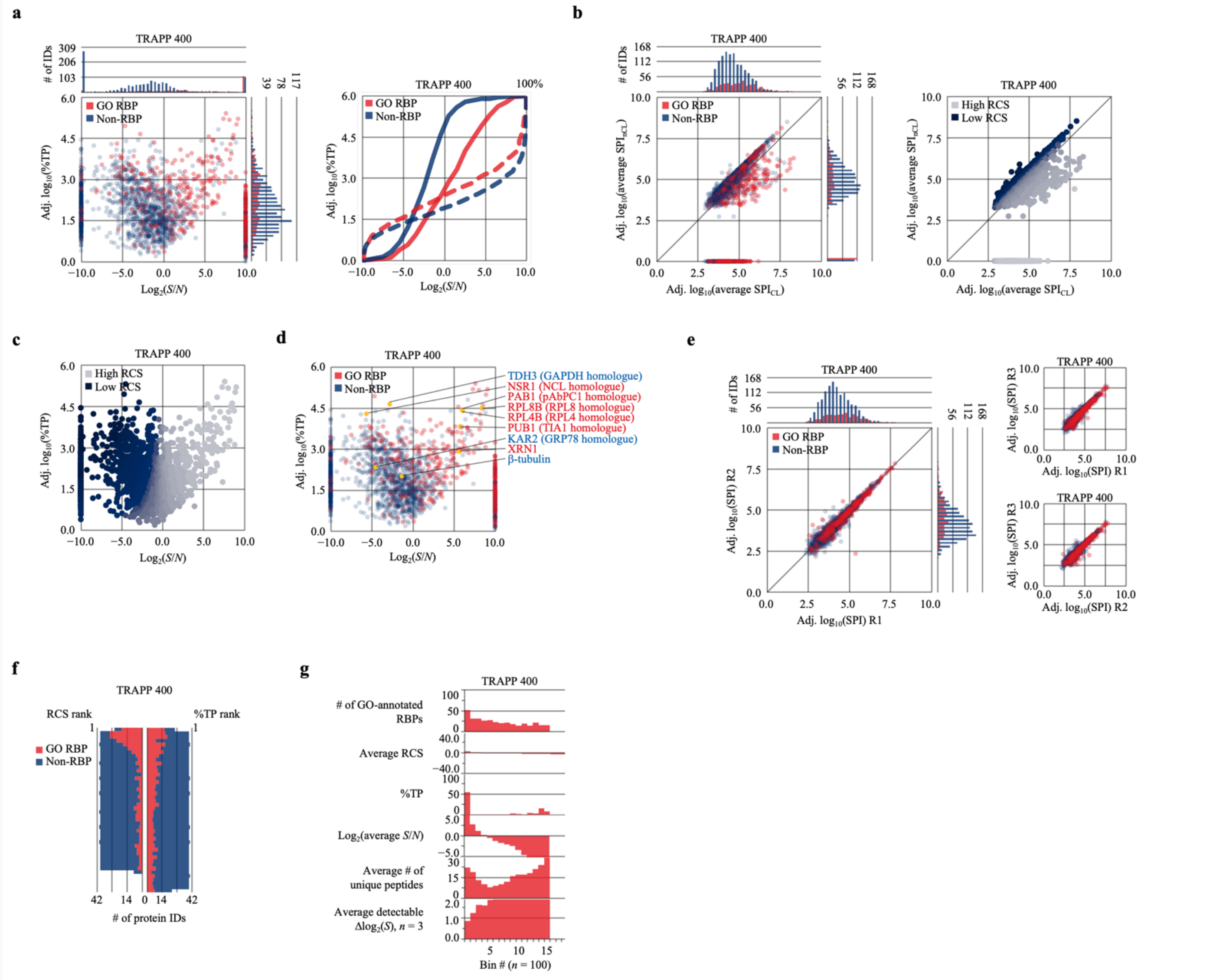

Supplementary Figure 39: Extended analysis of available MS data (SILAC) from referenced TRAPP study [4]. The following analyses were performed on the MS dataset provided in Supplementary Data 16. Notably, the referenced TRAPP study was performed in yeast and may limit comparison due to incomplete GO-annotations. **a** Method specificity for RNA-bound RBPs evaluated by comparing the abundance (%TP) of RBPs and non-RBPs as a function of their $\log_2(S/N)$ ratios. Corresponding frequency curves plotted as a function of $\log_2(S/N)$ (solid) or $\log_{10}$(%TP) (dashed). **b** Comparison of observed protein quantities in the UV-crosslinked and non-crosslinked SILAC channel after mean-normalization of total SPI (total SPI_{CL} = total SPI_{nCL}); color overlays based on GO-annotation status (red: RBP; blue: non-RBP) or ordinal RCS rank (silver: high RCS; navy blue: low RCS). **c** Main scatterplot from **a** with color overlay based on ordinal RCS rank. **d** Main scatterplot from **a** with labeled data points representing homologues of proteins evaluated by SRA and immunoblot in Fig. 9b. Color of text based on conclusions from SRA and immunoblot analysis of total clRNP fractions; red text: RNase-sensitive RBP; blue text: undetected or RNase-insensitive protein. **e** Comparison of observed protein quantities between replicate sample. **f** Comparison of RBP and non-RBP placement by RCS and %TP ranking. **g** RCS rank analysis. Source data are provided as a Source Data file.

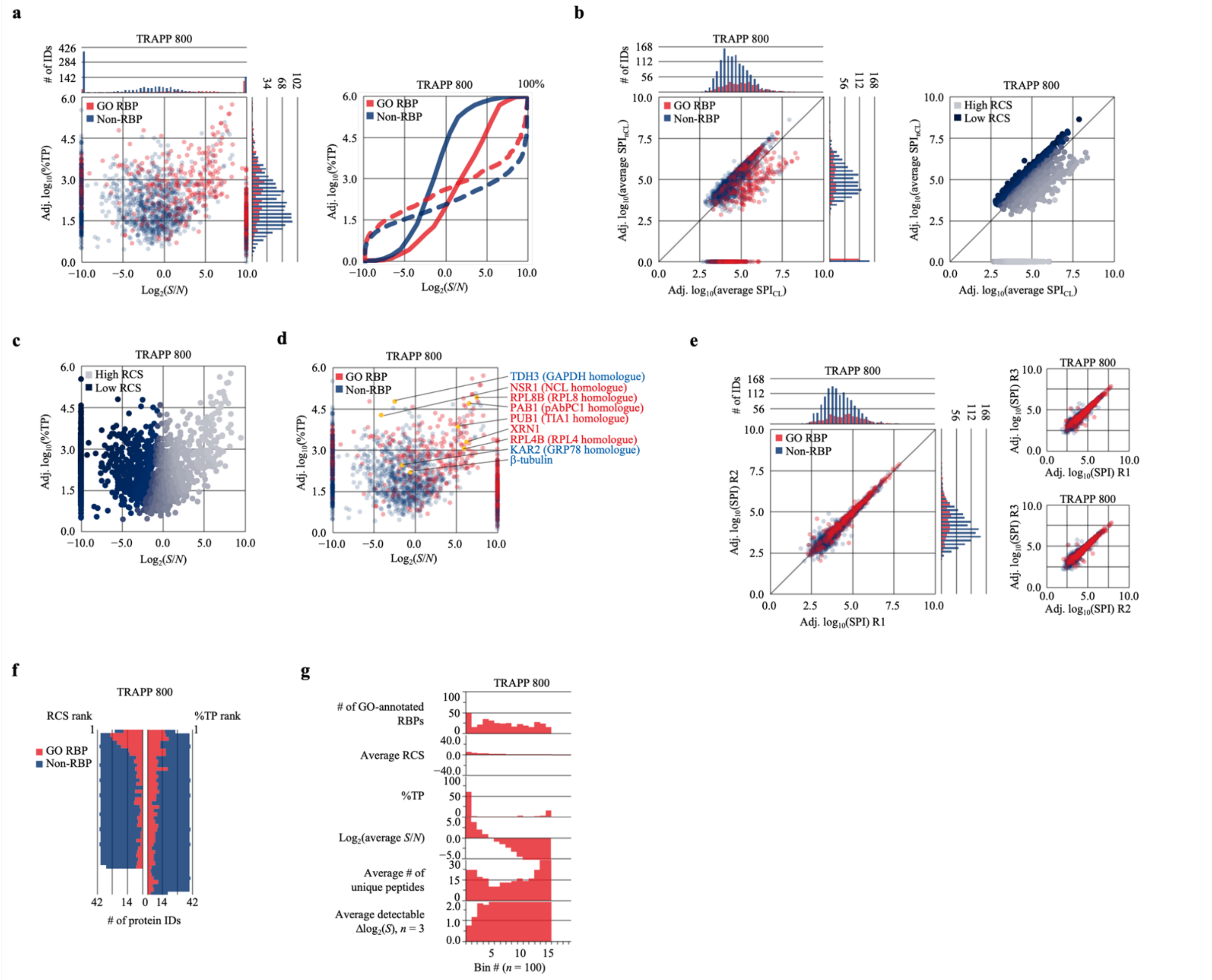

Supplementary Figure 40: Extended analysis of available MS data (SILAC) from referenced TRAPP study [4]. The following analyses were performed on the MS dataset provided in Supplementary Data 17. Notably, the referenced TRAPP study was performed in yeast and may limit comparison due to incomplete GO-annotations. **a** Method specificity for RNA-bound RBPs evaluated by comparing the abundance (%TP) of RBPs and non-RBPs as a function of their $\log_2(S/N)$ ratios. Corresponding frequency curves plotted as a function of $\log_2(S/N)$ (solid) or $\log_{10}$(%TP) (dashed). **b** Comparison of observed protein quantities in the UV-crosslinked and non-crosslinked SILAC channel after mean-normalization of total SPI (total SPI_{CL} = total SPI_{nCL}); color overlays based on GO-annotation status (red: RBP; blue: non-RBP) or ordinal RCS rank (silver: high RCS; navy blue: low RCS). **c** Main scatterplot from **a** with color overlay based on ordinal RCS rank. **d** Main scatterplot from **a** with labeled data points representing homologues of proteins evaluated by SRA and immunoblot in Fig. 9b. Color of text based on conclusions from SRA and immunoblot analysis of total clRNP fractions; red text: RNase-sensitive RBP; blue text: undetected or RNase-insensitive protein. **e** Comparison of observed protein quantities between replicate sample. **f** Comparison of RBP and non-RBP placement by RCS and %TP ranking. **g** RCS rank analysis. Source data are provided as a Source Data file.

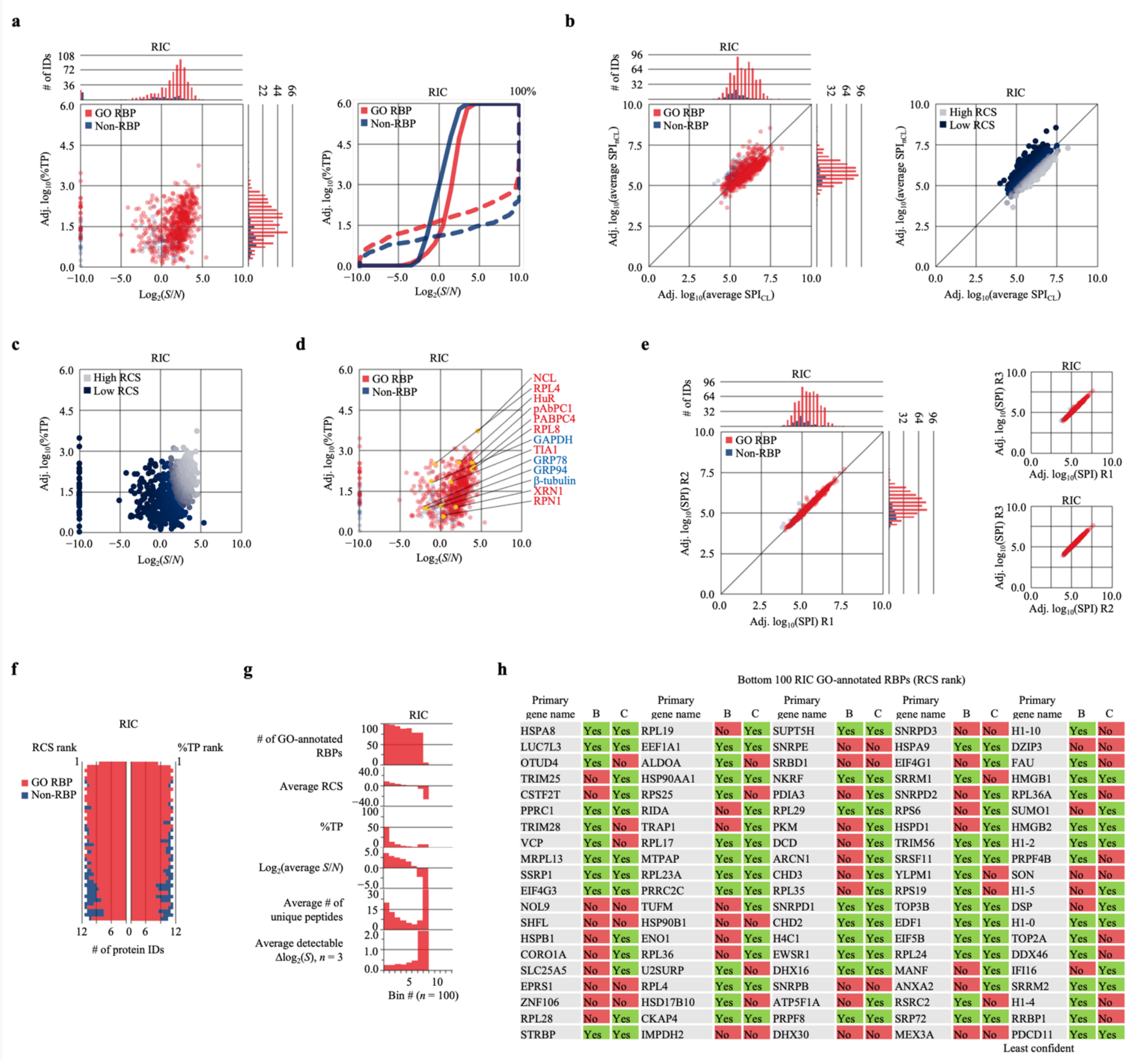

Primary gene name	B	C	Primary gene name	B	C	Primary gene name	B	C	Primary gene name	B	C	Primary gene name	B	C
HSPA8	Yes	Yes	RPL19	No	Yes	SUPT5H	Yes	Yes	SNRPD3	No	No	H1-10	Yes	No
LUC7L3	Yes	Yes	EEF1A1	Yes	Yes	SNRPE	No	No	HSPA9	Yes	Yes	DZIP3	No	No
OTUD4	Yes	No	ALDOA	Yes	No	SRBD1	No	No	EIF4G1	No	Yes	FAU	Yes	No
TRIM25	No	Yes	HSP90AA1	Yes	Yes	NKRF	Yes	Yes	SRRM1	Yes	No	HMGB1	Yes	Yes
CSTF2T	No	Yes	RPS25	Yes	No	PDIA3	No	Yes	SNRPD2	No	Yes	RPL36A	Yes	No
PPRC1	Yes	Yes	RIDA	No	Yes	RPL29	Yes	Yes	RPS6	No	Yes	SUMO1	No	Yes
TRIM28	Yes	No	TRAP1	No	Yes	PKM	No	Yes	HSPD1	No	Yes	HMGB2	Yes	Yes
VCP	Yes	No	RPL17	Yes	Yes	DCD	No	Yes	TRIM56	Yes	Yes	H1-2	Yes	Yes
MRPL13	Yes	Yes	MTPAP	Yes	Yes	ARCN1	No	Yes	SRSF11	Yes	Yes	PRPF4B	Yes	No
SSRP1	Yes	Yes	RPL23A	Yes	Yes	CHD3	No	Yes	YLPM1	Yes	No	SON	No	No
EIF4G3	Yes	Yes	PRRC2C	Yes	Yes	RPL35	No	Yes	RPS19	Yes	No	H1-5	No	Yes
NOL9	No	No	TUFM	No	Yes	SNRPD1	Yes	Yes	TOP3B	Yes	Yes	DSP	No	Yes
SHFL	No	No	HSP90B1	No	No	CHD2	Yes	Yes	EDF1	Yes	Yes	H1-0	Yes	Yes
HSPB1	No	Yes	ENO1	No	Yes	H4C1	Yes	Yes	EIF5B	Yes	Yes	TOP2A	Yes	No
CORO1A	No	Yes	RPL36	No	Yes	EWSR1	Yes	Yes	RPL24	Yes	Yes	DDX46	Yes	No
SLC25A5	No	Yes	U2SURP	Yes	No	DHX16	Yes	Yes	MANF	No	Yes	IFI16	No	Yes
EPRS1	No	No	RPL4	Yes	Yes	SNRPB	No	No	ANXA2	No	Yes	SRRM2	Yes	Yes
ZNF106	No	No	HSD17B10	Yes	No	ATP5F1A	No	Yes	RSRC2	Yes	No	H1-4	Yes	No
RPL28	No	Yes	CKAP4	Yes	Yes	PRPF8	Yes	Yes	SRP72	Yes	Yes	RRBP1	Yes	No
STRBP	Yes	Yes	IMPDH2	No	No	DHX30	No	No	MEX3A	No	No	PDCD11	Yes	Yes

Supplementary Figure 41: Extended analysis of available MS data (non-SILAC) from referenced RIC study [8]; not from the original RIC methods paper [24]. The following analyses were performed on the MS dataset provided in Supplementary Data 14. **a** Method specificity for RNA-bound RBPs evaluated by comparing the abundance (%TP) of RBPs and non-RBPs as a function of their $\log_2(S/N)$ ratios. Corresponding frequency curves plotted as a function of $\log_2(S/N)$ (solid) or $\log_{10}$(%TP) (dashed). **b** Comparison of observed protein quantities in UV-crosslinked and non-crosslinked samples after mean-normalization of total SPI (total SPI_{CL} = total SPI_{nCL}); color overlays based on GO-annotation status (red: RBP; blue: non-RBP) or ordinal RCS rank (silver: high RCS; navy blue: low RCS). **c** Main scatterplot from **a** with color overlay based on ordinal RCS rank. **d** Main scatterplot from **a** with labeled data points representing proteins evaluated by SRA and immunoblot in Fig. 9b. Color of text based on conclusions from SRA and immunoblot analysis of total clRNP fractions; red text: RNase-sensitive RBP; blue text: undetected or RNase-insensitive protein. **e** Comparison of observed protein quantities between replicate sample. **f** Comparison of RBP and non-RBP placement by RCS and %TP ranking. **g** RCS rank analysis. **h** Bottom 100 GO-annotated RBPs identified by RIC (RCS rank) with high throughput (HTP) evidence codes; B: [41]; C: [24]. Source data are provided as a Source Data file.

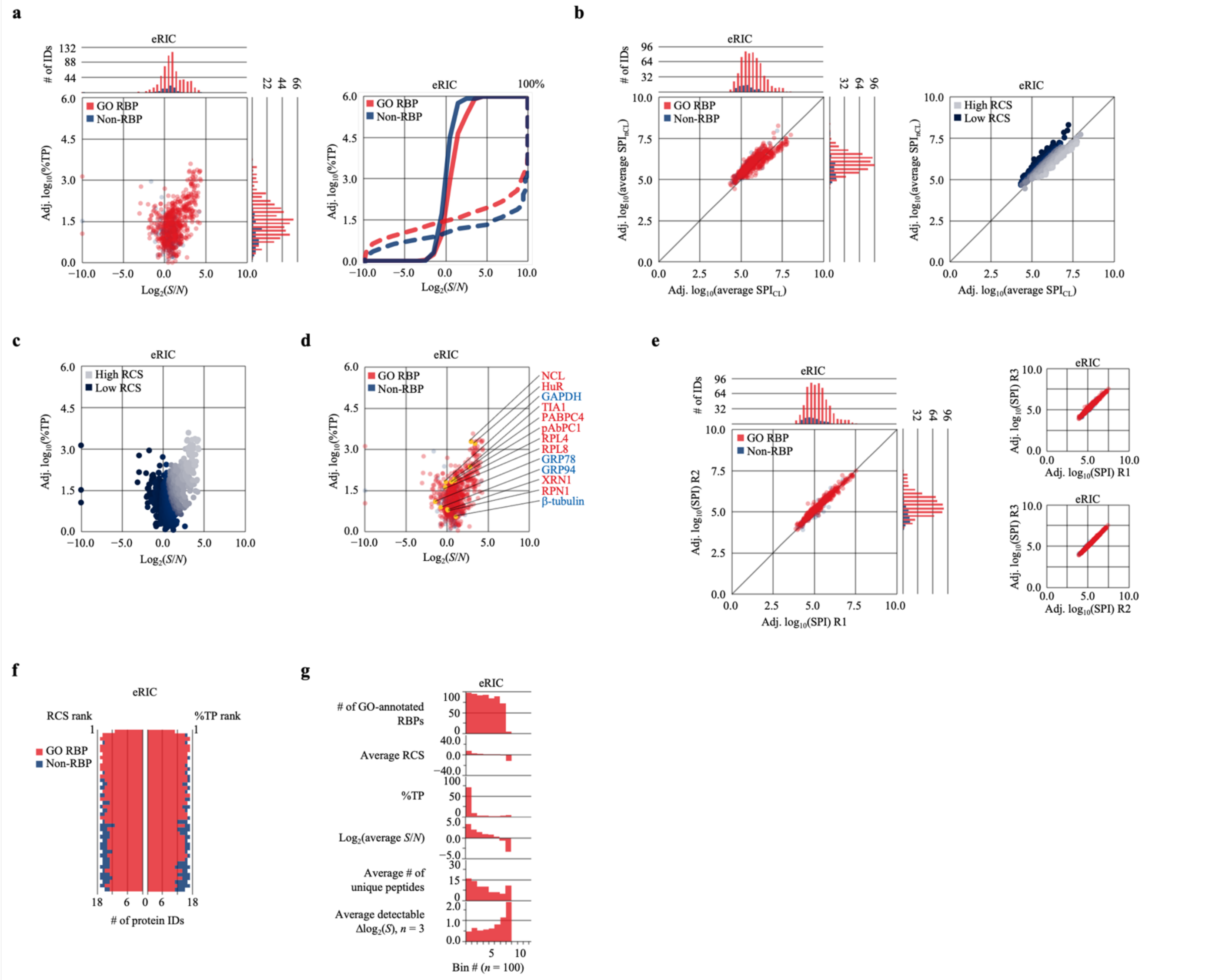

Supplementary Figure 42: Extended analysis of available MS data (non-SILAC) from referenced eRIC study [8]. The following analyses were performed on the MS dataset provided in Supplementary Data 15. **a** Method specificity for RNA-bound RBPs evaluated by comparing the abundance (%TP) of RBPs and non-RBPs as a function of their $\log_2(S/N)$ ratios. Corresponding frequency curves plotted as a function of $\log_2(S/N)$ (solid) or $\log_{10}$(%TP) (dashed). **b** Comparison of observed protein quantities in UV-crosslinked and non-crosslinked samples after mean-normalization of total SPI (total SPI_{CL} = total SPI_{nCL}); color overlays based on GO-annotation status (red: RBP; blue: non-RBP) or ordinal RCS rank (silver: high RCS; navy blue: low RCS). **c** Main scatterplot from **a** with color overlay based on ordinal RCS rank. **d** Main scatterplot from **a** with labeled data points representing proteins evaluated by SRA and immunoblot in Fig. 9b. Color of text based on conclusions from SRA and immunoblot analysis of total clRNP fractions; red text: RNase-sensitive RBP; blue text: undetected or RNase-insensitive protein. **e** Comparison of observed protein quantities between replicate sample. **f** Comparison of RBP and non-RBP placement by RCS and %TP ranking. **g** RCS rank analysis. Source data are provided as a Source Data file.

Supplementary Note 9
This note includes extended protocols for referenced RNA-centric methods.

9a. XRNAX [5].
XRNAX was performed on samples containing either 30 million UV-crosslinked (0.2 J/cm^2, 254 nm) or 30 million non-crosslinked HeLa cells according to the published protocol. Cells were harvested with two 800 µL aliquots of GT buffer and transferred to a 15 mL conical tube. Then, 800 µL phenol (acidic) were added and samples were triturated until no visible clumps remained. The samples were split between three 2 mL microcentrifuge tubes and 160 µL chloroform were added to each. Samples were inverted four times, incubated standing for 5 min at RT, and centrifuged at 7,000 x g for 10 min at 4 ˚C. Aqueous phases were removed and the interphase fractions were transferred to a 2 mL microcentrifuge tube. Interphase samples were washed twice with 0.3 mL TE + 0.1% SDS. The remaining interphase fractions were disintegrated using two 0.3 mL aliquots of TE + 0.1% SDS and two 0.3 mL aliquots of TE + 0.5% SDS by pipetting with each aliquot and transferring solubilized fractions to a 2 mL tube. Pooled solubilized interphase samples were mixed briefly and aliquoted between two 2 mL microcentrifuge tubes for isopropanol precipitation. To each aliquot, 36 µL 5.0 M NaCl, 0.6 µL Glycoblue, and 600 µL isopropanol were added. Samples were inverted several times and centrifuged at 18,000 x g for 15 min at 4 ˚C. Supernatants were removed and precipitates were washed with 0.3 mL RT 70% ethanol. Samples were spun down at 18,000 x g for 1 min at RT. Precipitates were air dried, 270 µL of DEPC-treated water were added, and samples were incubated overnight at 4 ˚C. Then, 30 µL 10X DNase I buffer, 0.3 µL RNaseOUT (10777019, Invitrogen), and 15 µL DNase I (M0303S, NEB) were added to each sample. Samples were incubated in a thermomixer for 90 min at 37 ˚C (700 rpm) and precipitated with 18 µL 5.0 M NaCl, 0.3 µL GlycoBlue, and 300 µL isopropanol. Samples were inverted several times and centrifuged at 18,000 x g for 15 min at 4 ˚C. Supernatants were removed and precipitates were washed with 0.15 mL room-temperature 70% ethanol by pipetting. Samples were centrifuged down at 18,000 x g for 1 min at RT. An additional transfer/washing step was used to improve solubilization of precipitates: three 400 µL aliquots of RT 95% methanol were used to recover precipitates adhering to the sides of the tubes and combined in a 1.5 mL microcentrifuge tube. The tubes were then placed vertically at 4 ˚C to allow precipitate settling at the bottom of the tube. Samples were centrifuged at 20,000 x g for 10 min at 20 ˚C and supernatants were removed. Pellets were air dried and resuspended at the desired concentration with 1% LiDS TE.

9b. OOPs [13].
OOPs was performed on samples containing either 30 million UV-crosslinked (0.2 J/cm^2, 254 nm) or 30 million non-crosslinked HeLa cells according to the published protocol. Cells were harvested with two 1 mL aliquots of GT buffer and transferred to a 15 mL conical tube. Then, 1 mL phenol (acidic) was added and samples were triturated until no visible clumps remained. The samples were split between three 2 mL microcentrifuge tubes and 200 µL chloroform were added to each. Samples were vortexed (max) for 15 sec and centrifuged at 12,000 x g for 15 min at 4 ˚C. A gel loading pipette tip was used to remove the aqueous and organic phases while leaving the interphase undisturbed. 1 mL of fresh acidic guanidinium thiocyanate-phenol (2:1) buffer was added and interphase samples were resolubilized by pipetting. Then, 200 µL chloroform were added and samples were AGPC extracted as before. This process was repeated for a total of three AGPC extractions. Then, 9 volumes of RT 100% methanol (~1.35 mL) were added to interphase samples and immediately centrifuged at 14,000 x g for 10 min at 4 ˚C. Precipitates were washed twice with 1 mL RT 95% methanol by pipetting and centrifuged at 14,000 x g for 10 min at 4 ˚C. Three 400 µL aliquots of RT 95% methanol were used to recover precipitates adhering to the sides of the tubes and combined (pool aliquots) in a 1.5 mL microcentrifuge tube. The tubes were then placed vertically and incubated for 30 min at RT to allow precipitate settling at the bottom of the tube. Samples were centrifuged at 14,000 x g for 10 min, supernatants were removed, and precipitated were air dried. 70 µL TE buffer were added and samples were incubated overnight at 4 ˚C followed by pipetting until precipitates solubilized. RNase-digestion was performed in separate 1.5 mL microcentrifuge tubes using 30 µL of TE-suspended interphase samples. RNase Cocktail (AM2286, Invitrogen), 10X RNase digest buffer (100 mM Tris-HCl pH 7.5, 1 M NaCl, and 10 mM EDTA), and 25X protease inhibitors (11836153001, Roche) were added at the same time to a final concentration of 2 µL RNase Cocktail/15 µg protein-bound RNA, 1X RNase digestion buffer, and 1X protease inhibitors (100 µL total reaction volume). Untreated control samples were set up without RNase

Cocktail, and both were incubated for 2 hours at 37 ˚C. The recommended RNase-digest conditions do not include RNase digestion buffer and involve overnight incubation at 37 ˚C. The additional (optional) MeOH washes were found to improve subsequent solubilization. This, along with the addition of RNase digestion buffer, was found to facilitate efficient digestion of RNA within a timeframe that avoided protein degradation. 1 mL of fresh acidic guanidinium thiocyanate-phenol (2:1) were added to each sample followed by brief vortex. 200 µL chloroform were added and samples were vortexed for 15 sec. Samples were centrifuged at 12,000 x g for 15 min at 4 ˚C. The upper aqueous phase and interphase fractions were removed, and three 150 µL aliquots of the organic phase were each transferred to 1.5 mL microcentrifuge tubes containing 1.35 mL RT 100% methanol. Samples were vortexed for 15 sec and centrifuged at 20,000 x g for 10 min at 4 ˚C. Precipitates were washed twice with 1 mL RT 95% methanol by pipetting and centrifuged at 14,000 x g for 10 min at 4 ˚C. Three 400 µL aliquots of RT 95% methanol were used to recover precipitates adhering to the sides of the tubes and combined in a 1.5 mL microcentrifuge tube. The tubes were then placed vertically and incubated for 30 min at RT to allow precipitate settling at the bottom of the tube. Samples were centrifuged at 14,000 x g for 10 min and supernatants were removed. Pellets were air dried and resuspended at the desired concentration with 1% LiDS TE.

9c. Ptex [7].

Ptex was performed on samples containing either 30 million UV-crosslinked (0.2 J/cm^2, 254 nm) or 30 million non-crosslinked HeLa cells according to the published protocol. Cells were harvested with two 1 mL aliquots of ice-cold 1X PBS and transferred to a 15 mL conical tube. Additional 1X PBS was added to each sample for a final volume of 2.25 mL. 750 µL neutral phenol, 750 µL toluol (244511, Sigma-Aldrich), and 750 µL 1,3-bromochloropropane (BCP) (B9673, Sigma-Aldrich) were added and samples were triturated until no visible clumps remained. Samples were aliquoted between three 2 mL microcentrifuge tubes and mixed at 2,000 rpm for 1 minute at RT. Samples were centrifuged at 20,000 x g for 3 min at 4 ˚C. The aqueous phases were each transferred to 2 mL microcentrifuge tubes containing 300 µL solution D. 600 µL neutral phenol and 200 µL BCP were added. Samples were mixed at 2,000 rpm for 1 min at RT and centrifuged at 20,000 x g for 3 min at 4 ˚C. 3/4th of the aqueous and organic phases were removed and 400 µL DEPC-treated water, 200 µL 100% ethanol, 400 µL neutral phenol, and 200 µL BCP were added to each sample. Samples were mixed at 2,000 rpm for 1 min at RT and centrifuged at 20,000 x g for 3 min at 4 ˚C. A gel loading pipette tip was used to remove the aqueous and organic phases while leaving the interphase undisturbed. 9 volumes of 100% ethanol were added to each sample and incubated overnight at −20˚C. The next day, samples were spun down at 20,000 x g for 30 min at 4 ˚C and supernatants were removed. To ensure removal of salts prior to resuspension and RNase-digestion, pellets were washed twice with 1.0 mL ice-cold 75% ethanol. For each wash, samples were incubated on ice for 5 min followed by centrifugation at 18,000 x g for 5 min at 4 ˚C and removal of supernatant. Then, three 400 µL aliquots of ice-cold 75% ethanol were used to recover precipitates adhering to the sides of the tubes and combined (pool aliquots) in a 1.5 mL microcentrifuge tube. The tubes were then placed vertically and incubated for 30 min on ice to allow precipitate settling at the bottom of the tube. Samples were centrifuged at 18,000 x g for 5 min at 4 ˚C and supernatants were removed. Pellets were air dried and resuspended at the desired concentration with 1% LiDS TE.

9d. TRAPP [4].

TRAPP was performed on samples containing either 30 million UV-crosslinked (0.2 J/cm^2, 254 nm) or 30 million non-crosslinked HeLa cells according to the published protocol. Cells were harvested with two 600 µL aliquots of GT buffer and transferred to a 15 mL conical tube. Then, 1.2 mL phenol (acidic) were added and lysates were sheered by passaging through a 19 ga 1-1/2” needle fifteen times. Samples were centrifuged at 4,600 x g for 5 min at 4 ˚C and supernatants were transferred to 15 mL conical tubes. Samples were centrifuged at 13,000 x g for 10 min at 4 ˚C and supernatants were transferred to a 15 mL conical tube (~2.65 mL per clarified sample with residual PBS). 270 µL 3 M sodium acetate-acetic acid pH 4.0 were added to each tube and samples were mixed briefly. 3 mL RT 100% ethanol were added slowly to samples and then mixed by vortex (5 sec). 1.5 mL of equilibrated 50% silica bead slurry (S5631, Sigma Aldrich) were added to each sample followed by 1.5 mL RT 100% ethanol; silica beads were equilibrated by incubating overnight in 1 M HCl and washed several times with DEPC-treated water. Samples were vortexed briefly to fully resuspend beads and incubated on a rotator for 60 min at RT. Samples were centrifuged at 2,500 x g for 2 min at 4 ˚C and supernatants were removed. Silica beads were resuspended by vigorous vortexing in 4.5 mL wash buffer

I (4 M guanidine thiocyanate, 1 M sodium acetate-acetic acid pH 4.0, and 30% ethanol); 30 sec. Samples were centrifuged at 2,500 x g for 2 min at 4 ˚C and supernatants were removed. This wash step was repeated two more times (wash buffer I), followed by three washes using 4.5 mL wash buffer II (100 mM NaCl, 50 mM Tris-HCl pH 6.4, and 80% ethanol). After the 3rd wash with wash buffer II, silica beads were transferred to 2.0 mL microcentrifuge tubes using three 500 µL aliquots of WB2 and centrifuged at 2,500 x g for 2 minat 4 ˚C. Supernatants were removed and silica beads were dried. RNPs were heat eluted using four 500 µL aliquots of 20 mM Tris-HCl pH 7.5 + 1 mM EDTA pH 8.0. Each time, samples were incubated at 55 ˚C for 2 min, vortexed for 10 sec and centrifuged at 4,000 x g for 1 min at 20 ˚C. Supernatants were transferred to a 2 ml microcentrifuge tube. After pooling all four aliquots, samples were incubated at 55 ˚C for 2 min, vortexed for 10 sec, and centrifuged at 14,000 x g for 1 min at 20 ˚C. This sequence was repeated. Samples were split between four 2 mL microcentrifuge tubes each containing 3.0 µL GlycoBlue and mixed by brief vortex. Then, 68.6 µL 5 M NaCl were added to each (0.6 M final) and mixed by brief vortex. 1.143 mL RT 100% isopropanol were added to each fraction, vortexed, and incubated on rotator overnight at 4 ˚C. Samples were centrifuged at 18,000 x g for 15 min at 4 ˚C and supernatants were removed. To ensure removal of salts prior to resuspension and RNase-digestion, pellets were washed twice with 1 mL ice-cold 75% ethanol. For each wash, samples were incubated on ice for 5 min followed by centrifugation at 18,000 x g for 5 min at 4 ˚C and removal of supernatant. Then, three 400 µL aliquots of ice-cold 75% ethanol were used to recover precipitates adhering to the sides of the tubes and combined in a 1.5 mL microcentrifuge tube. The tubes were then placed vertically and incubated for 30 min on ice to allow precipitate settling at the bottom of the tube. Samples were centrifuged at 18,000 x g for 5 min at 4 ˚C and supernatants were removed. Pellets were air dried and resuspended at the desired concentration with 1% LiDS TE. An RNase elution was also performed following heat elution as a control. Beads were resuspended in 430 µL DEPC-treated water, 50 µL 10X RNase buffer, and 20 µL RNase Cocktail (AM2286, Invitrogen). Samples were incubated on a rotator at 37 ˚C for 2 hr, incubated at 55 ˚C for 2 min, vortexed for 10 sec, and centrifuged at 4,000 x g for 1 min at 20 ˚C. Supernatants were transferred to a fresh 2 mL microcentrifuge tube and incubated at 55 ˚C for 2 min, vortexed for 10 sec, and centrifuged at 14,000 x g for 1 min at 20 ˚C. This was repeated and clarified supernatants were transferred to 15 mL conical tubes containing 10 mL RT 100% methanol. Samples were incubated on a rotator overnight at RT. Samples were transferred to a 2.0 mL microcentrifuge tube and centrifuged at 20,000 x g for 10 min at 20 ˚C; supernatants were removed and discarded after each spin. Precipitates were washed twice with 1.0 mL RT 95% methanol. For each wash, samples were vortexed for at least 5 sec, incubated on a rotator for at least 10 min at RT, and centrifuged at 20,000 x g for at least 10 min at 20 ˚C. Then, three 400 µL aliquots of RT 95% methanol were used to recover precipitates adhering to the sides of the tubes and combined in a 1.5 mL microcentrifuge tube. The tubes were then placed vertically for 1 hour at RT allow precipitates to settle at the bottom of the tube. Samples were centrifuged at 20,000 x g for at least 10 min at 20 ˚C and supernatants were removed. Pellets were air dried and resuspended at the desired concentration with 1% LiDS TE.

9e. RNA-interactome capture [24].

RIC was performed on samples containing either 30 million UV-crosslinked (0.2 J/cm^2, 254 nm) or 30 million non-crosslinked HeLa cells according to the published protocol using Oligo d(T)$_{25}$ magnetic beads (S1419S, NEB). Cells were harvested with two 15 mL aliquots of lysis/binding buffer (100 mM Tris-HCl, pH 7.5, 500 mM LiCl, 0.5% LiDS, 1 mM EDTA pH 8.0, and 5 mM DTT) and transferred to 50 mL conical tubes. Lysates were sheered by passaging through a 19 ga 1-1/2" needle fifteen times. 3 mL equilibrated oligo d(T)$_{25}$ magnetic bead slurry were added to each sample and incubated on agitator for 10 min at RT. A magnet was used to recover beads and supernatants were removed. Beads were washed twice with 15 mL wash buffer 1 (20 mM Tris-HCl pH 7.5, 500 mM LiCl, 0.1% LiDS, 1 mM EDTA pH 8.0, and 5 mM DTT), twice with 15 mL wash buffer 2 (20 mM Tris-HCl pH 7.5, 500 mM LiCl, and 1 mM EDTA pH 8.0), and once 15 mL low salt buffer (20 mM Tris-HCl pH 7.5, 200 mM LiCl, and 1 mM EDTA pH 8.0). For each wash, samples were mixed with agitation for 1 min, beads were recovered using a magnet, and supernatants were removed. Two 2 mL aliquots of wash buffer 2 were used to transfer beads to a fresh 2 mL microcentrifuge tube, a magnet was used to recover beads and remove supernatant each time. RNPs were heat eluted using four 500 µL aliquots of 20 mM Tris-HCl pH 7.5, 1 mM EDTA pH 8.0. Each time, samples were incubated at 55 ˚C for 2 min and vortexed for 10 sec (max). After pooling all four aliquots, samples were incubated at 55 ˚C for 2 min and vortexed for 10 sec (max). Beads were recovered and supernatants were transferred to a 2 mL microcentrifuge tube. Samples were split between four 2 mL microcentrifuge tubes each containing 3.0 µL

GlycoBlue and mixed by brief vortex. Then, 68.6 µL 5 M NaCl were added to each (0.6 M final) and mixed by brief vortex. 1.143 mL RT 100% isopropanol were added to each fraction, vortexed, and incubated on rotator overnight at 4 ˚C. Samples were centrifuged at 18,000 x g for 15 min at 4 ˚C and supernatants were removed. To ensure removal of salts prior to resuspension and RNase-digestion, pellets were washed twice with 1 mL ice-cold 75% ethanol. For each wash, samples were incubated on ice for 5 min followed by centrifugation at 18,000 x g for 5 min at 4 ˚C and removal of supernatant. Then, three 400 µL aliquots of ice-cold 75% ethanol were used to recover precipitates adhering to the sides of the tubes and combined in a 1.5 mL microcentrifuge tube. The tubes were then placed vertically and incubated for 30 min on ice to allow precipitate settling at the bottom of the tube. Samples were centrifuged at 18,000 x g for 5 min at 4 ˚C (soft brake) and supernatants were removed. Pellets were air dried and resuspended at the desired concentration with 1% LiDS TE. An RNase elution was also performed following heat elution as a control. Beads were resuspended in 430 µL DEPC-treated water, 50 µL 10X RNase buffer, and 20 µL RNase Cocktail (AM2286, Invitrogen). Samples were incubated on a rotator at 37 ˚C for 2 hr, incubated at 55 ˚C for 2 min, and vortexed for 10 sec. Beads were recovered and supernatants were transferred to a 2 ml microcentrifuge tube. Samples were incubated again at 55 ˚C for 2 min and vortexed for 10 sec. Beads were recovered and supernatants were transferred to 15 mL conical tubes containing 10 mL RT 100% methanol. Samples were incubated on rotator overnight at RT. Samples were transferred to a 2 mL microcentrifuge tube and centrifuged at 20,000 x g for 10 min at 20 ˚C; supernatants were removed and discarded after each spin. Precipitates were washed twice with 1 mL RT 95% methanol. For each wash, samples were vortexed for at least 5 sec, incubated on a rotator for at least 10 min at RT, and centrifuged at 20,000 x g for at least 10 min at 20 ˚C. Then, three 400 µL aliquots of RT 95% methanol were used to recover precipitates adhering to the sides of the tubes and combined in a 1.5 mL microcentrifuge tube. The tubes were then placed vertically for 1 hour at RT allow precipitates to settle at the bottom of the tube. Samples were centrifuged at 20,000 x g for at least 10 min at 20 ˚C and supernatants were removed. Pellets were air dried and resuspended at the desired concentration with 1% LiDS TE.

Supplementary References

1. Wisniewski, J.R., et al., *Extensive quantitative remodeling of the proteome between normal colon tissue and adenocarcinoma.* Mol Syst Biol, 2012. **8**: p. 611.
2. Wisniewski, J.R. and D. Rakus, *Multi-enzyme digestion FASP and the 'Total Protein Approach'-based absolute quantification of the Escherichia coli proteome.* J Proteomics, 2014. **109**: p. 322-31.
3. Wisniewski, J.R., *Label-Free and Standard-Free Absolute Quantitative Proteomics Using the "Total Protein" and "Proteomic Ruler" Approaches.* Methods Enzymol, 2017. **585**: p. 49-60.
4. Shchepachev, V., et al., *Defining the RNA interactome by total RNA-associated protein purification.* Mol Syst Biol, 2019. **15**(4): p. e8689.
5. Trendel, J., et al., *The Human RNA-Binding Proteome and Its Dynamics during Translational Arrest.* Cell, 2019. **176**(1-2): p. 391-403 e19.
6. Hoefig, K.P., et al., *Defining the RBPome of primary T helper cells to elucidate higher-order Roquin-mediated mRNA regulation.* Nat Commun, 2021. **12**(1): p. 5208.
7. Urdaneta, E.C., et al., *Purification of cross-linked RNA-protein complexes by phenol-toluol extraction.* Nat Commun, 2019. **10**(1): p. 990.
8. Perez-Perri, J.I., et al., *Discovery of RNA-binding proteins and characterization of their dynamic responses by enhanced RNA interactome capture.* Nat Commun, 2018. **9**(1): p. 4408.
9. Bantscheff, M., et al., *Quantitative mass spectrometry in proteomics: a critical review.* Anal Bioanal Chem, 2007. **389**(4): p. 1017-31.
10. Jankowsky, E. and M.E. Harris, *Specificity and nonspecificity in RNA-protein interactions.* Nat Rev Mol Cell Biol, 2015. **16**(9): p. 533-44.
11. Chomczynski, P. and N. Sacchi, *Single-step method of RNA isolation by acid guanidinium thiocyanate-phenol-chloroform extraction.* Anal Biochem, 1987. **162**(1): p. 156-9.
12. Chomczynski, P. and N. Sacchi, *The single-step method of RNA isolation by acid guanidinium thiocyanate-phenol-chloroform extraction: twenty-something years on.* Nat Protoc, 2006. **1**(2): p. 581-5.
13. Queiroz, R.M.L., et al., *Comprehensive identification of RNA-protein interactions in any organism using orthogonal organic phase separation (OOPS).* Nat Biotechnol, 2019. **37**(2): p. 169-178.
14. Corley, M., M.C. Burns, and G.W. Yeo, *How RNA-Binding Proteins Interact with RNA: Molecules and Mechanisms.* Mol Cell, 2020. **78**(1): p. 9-29.
15. Erlendsson, S. and K. Teilum, *Binding Revisited-Avidity in Cellular Function and Signaling.* Front Mol Biosci, 2020. **7**: p. 615565.
16. Khong, A. and R. Parker, *The landscape of eukaryotic mRNPs.* RNA, 2020. **26**(3): p. 229-239.
17. Sharma, D., et al., *The kinetic landscape of an RNA-binding protein in cells.* Nature, 2021. **591**(7848): p. 152-156.
18. Knorlein, A., et al., *Nucleotide-amino acid pi-stacking interactions initiate photo cross-linking in RNA-protein complexes.* Nat Commun, 2022. **13**(1): p. 2719.
19. Licatalosi, D.D., X. Ye, and E. Jankowsky, *Approaches for measuring the dynamics of RNA-protein interactions.* Wiley Interdiscip Rev RNA, 2020. **11**(1): p. e1565.
20. Vieira-Vieira, C.H. and M. Selbach, *Opportunities and Challenges in Global Quantification of RNA-Protein Interaction via UV Cross-Linking.* Front Mol Biosci, 2021. **8**: p. 669939.
21. Castello, A., et al., *Comprehensive Identification of RNA-Binding Domains in Human Cells.* Mol Cell, 2016. **63**(4): p. 696-710.
22. Cox, J., et al., *Accurate proteome-wide label-free quantification by delayed normalization and maximal peptide ratio extraction, termed MaxLFQ.* Mol Cell Proteomics, 2014. **13**(9): p. 2513-26.
23. Domon, B. and R. Aebersold, *Mass spectrometry and protein analysis.* Science, 2006. **312**(5771): p. 212-7.
24. Castello, A., et al., *Insights into RNA biology from an atlas of mammalian mRNA-binding proteins.* Cell, 2012. **149**(6): p. 1393-406.
25. Carrillo, B., et al., *Methods for combining peptide intensities to estimate relative protein abundance.* Bioinformatics, 2010. **26**(1): p. 98-103.

26. Vergeynst, L., et al., *Accurate mass determination, quantification and determination of detection limits in liquid chromatography-high-resolution time-of-flight mass spectrometry: challenges and practical solutions.* Anal Chim Acta, 2013. **789**: p. 74-82.
27. Andreasson, U., et al., *A Practical Guide to Immunoassay Method Validation.* Front Neurol, 2015. **6**: p. 179.
28. Lee, J.W., et al., *Fit-for-purpose method development and validation for successful biomarker measurement.* Pharm Res, 2006. **23**(2): p. 312-28.
29. Izquierdo, J.M. and J. Valcarcel, *Two isoforms of the T-cell intracellular antigen 1 (TIA-1) splicing factor display distinct splicing regulation activities. Control of TIA-1 isoform ratio by TIA-1-related protein.* J Biol Chem, 2007. **282**(27): p. 19410-7.
30. Bauer, W.J., et al., *Three RNA recognition motifs participate in RNA recognition and structural organization by the pro-apoptotic factor TIA-1.* J Mol Biol, 2012. **415**(4): p. 727-40.
31. Cruz-Gallardo, I., et al., *RNA binding of T-cell intracellular antigen-1 (TIA-1) C-terminal RNA recognition motif is modified by pH conditions.* J Biol Chem, 2013. **288**(36): p. 25986-25994.
32. Hamdollah Zadeh, M.A., et al., *Alternative splicing of TIA-1 in human colon cancer regulates VEGF isoform expression, angiogenesis, tumour growth and bevacizumab resistance.* Mol Oncol, 2015. **9**(1): p. 167-78.
33. Garcia-Maurino, S.M., et al., *RNA Binding Protein Regulation and Cross-Talk in the Control of AU-rich mRNA Fate.* Front Mol Biosci, 2017. **4**: p. 71.
34. Hentze, M.W., et al., *A brave new world of RNA-binding proteins.* Nat Rev Mol Cell Biol, 2018. **19**(5): p. 327-341.
35. Ramanathan, M., D.F. Porter, and P.A. Khavari, *Methods to study RNA-protein interactions.* Nat Methods, 2019. **16**(3): p. 225-234.
36. Tawk, C., et al., *A systematic analysis of the RNA-targeting potential of secreted bacterial effector proteins.* Sci Rep, 2017. **7**(1): p. 9328.
37. Lin, C. and W.O. Miles, *Beyond CLIP: advances and opportunities to measure RBP-RNA and RNA-RNA interactions.* Nucleic Acids Res, 2019. **47**(11): p. 5490-5501.
38. Vaishali, et al., *Validation and classification of RNA binding proteins identified by mRNA interactome capture.* RNA, 2021. **27**(10): p. 1173-1185.
39. Gerstberger, S., M. Hafner, and T. Tuschl, *A census of human RNA-binding proteins.* Nat Rev Genet, 2014. **15**(12): p. 829-45.
40. Van Ende, R., S. Balzarini, and K. Geuten, *Single and Combined Methods to Specifically or Bulk-Purify RNA-Protein Complexes.* Biomolecules, 2020. **10**(8).
41. Baltz, A.G., et al., *The mRNA-bound proteome and its global occupancy profile on protein-coding transcripts.* Mol Cell, 2012. **46**(5): p. 674-90.
42. Balcerak, A., et al., *RNA-protein interactions: disorder, moonlighting and junk contribute to eukaryotic complexity.* Open Biol, 2019. **9**(6): p. 190096.
43. Castello, A., M.W. Hentze, and T. Preiss, *Metabolic Enzymes Enjoying New Partnerships as RNA-Binding Proteins.* Trends Endocrinol Metab, 2015. **26**(12): p. 746-757.
44. Garcin, E.D., *GAPDH as a model non-canonical AU-rich RNA binding protein.* Semin Cell Dev Biol, 2019. **86**: p. 162-173.
45. Kroning, N., et al., *ATP binding to the KTN/RCK subunit KtrA from the K+ -uptake system KtrAB of Vibrio alginolyticus: its role in the formation of the KtrAB complex and its requirement in vivo.* J Biol Chem, 2007. **282**(19): p. 14018-27.
46. Kramer, K., et al., *Photo-cross-linking and high-resolution mass spectrometry for assignment of RNA-binding sites in RNA-binding proteins.* Nat Methods, 2014. **11**(10): p. 1064-70.
47. Hentze, M.W., *Enzymes as RNA-binding proteins: a role for (di)nucleotide-binding domains?* Trends Biochem Sci, 1994. **19**(3): p. 101-3.
48. Singh, R. and M.R. Green, *Sequence-specific binding of transfer RNA by glyceraldehyde-3-phosphate dehydrogenase.* Science, 1993. **259**(5093): p. 365-8.
49. Bonafe, N., et al., *Glyceraldehyde-3-phosphate dehydrogenase binds to the AU-Rich 3' untranslated region of colony-stimulating factor-1 (CSF-1) messenger RNA in human ovarian cancer cells: possible role in CSF-1 posttranscriptional regulation and tumor phenotype.* Cancer Res, 2005. **65**(9): p. 3762-71.

50. Fillebeen, C., N. Wilkinson, and K. Pantopoulos, *Electrophoretic mobility shift assay (EMSA) for the study of RNA-protein interactions: the IRE/IRP example.* J Vis Exp, 2014(94).
51. Rio, D.C., *Electrophoretic mobility shift assays for RNA-protein complexes.* Cold Spring Harb Protoc, 2014. **2014**(4): p. 435-40.
52. Meisenheimer, K.M. and T.H. Koch, *Photocross-linking of nucleic acids to associated proteins.* Crit Rev Biochem Mol Biol, 1997. **32**(2): p. 101-40.
53. Saito, I., H. Sugiyama, and T. Matsuura, *Photochemical reactions of nucleic acids and their constituents of photobiological relevance.* Photochem Photobiol, 1983. **38**(6): p. 735-43.
