# Supplementary material for: Signal-noise metrics for RNA binding protein identification reveal broad spectrum protein-RNA interaction frequencies and dynamics
Source: Nat Commun. 2023 Sep 21;14:5868. doi: 10.1038/s41467-023-41284-9 (PMC10514315; doi:10.1038/s41467-023-41284-9)
Supplement: Supplementary file 2 — Reporting Summary [file 41467_2023_41284_MOESM2_ESM.pdf]

nature portfolio

Corresponding author(s): Christopher V. Nicchitta

Last updated by author(s): Aug 25, 2023

Reporting Summary

Nature Portfolio wishes to improve the reproducibility of the work that we publish. This form provides structure for consistency and transparency in reporting. For further information on Nature Portfolio policies, see our Editorial Policies and the Editorial Policy Checklist.

Statistics

For all statistical analyses, confirm that the following items are present in the figure legend, table legend, main text, or Methods section.

n/a	Confirmed	
☐	☒	The exact sample size (*n*) for each experimental group/condition, given as a discrete number and unit of measurement
☐	☒	A statement on whether measurements were taken from distinct samples or whether the same sample was measured repeatedly
☐	☒	The statistical test(s) used AND whether they are one- or two-sided<br>*Only common tests should be described solely by name; describe more complex techniques in the Methods section.*
☐	☒	A description of all covariates tested
☐	☒	A description of any assumptions or corrections, such as tests of normality and adjustment for multiple comparisons
☐	☒	A full description of the statistical parameters including central tendency (e.g. means) or other basic estimates (e.g. regression coefficient) AND variation (e.g. standard deviation) or associated estimates of uncertainty (e.g. confidence intervals)
☐	☒	For null hypothesis testing, the test statistic (e.g. *F*, *t*, *r*) with confidence intervals, effect sizes, degrees of freedom and *P* value noted<br>*Give P values as exact values whenever suitable.*
☒	☐	For Bayesian analysis, information on the choice of priors and Markov chain Monte Carlo settings
☒	☐	For hierarchical and complex designs, identification of the appropriate level for tests and full reporting of outcomes
☐	☒	Estimates of effect sizes (e.g. Cohen's *d*, Pearson's *r*), indicating how they were calculated

Our web collection on statistics for biologists contains articles on many of the points above.

Software and code

Policy information about availability of computer code

Data collection	For mass spectrometry experiments, data were collected using a Thermo Orbitrap Fusion Lumos high resolution accurate mass tandem mass spectrometer (Thermo). For RNA sequencing experiments, data were collected using a Illumina NovaSeq 6000.
Data analysis	All statistical analyses were performed using JMPro14.0. A detailed description of the analytical pipeline used for analysis of LC-MS/MS raw data is provided in the Methods as well as in the Supplementary Information file, subsection 7: MS data analysis. For the INP SILAC LC-MS/MS experiment, MS raw files were processed using Proteome Discoverer 2.4 (Thermo Scientific Inc.) and Minora Feature Detector algorithm in Proteome Discoverer. Mascot Distiller and Mascot Server (v 2.5, Matrix sciences) were utilized to produce fragment ion spectra and to perform the database searches. Peptide Validator and Protein FDR Validator nodes in Proteome Discoverer were used for data annotation. For the LEAP-RBP SILAC LC-MS/MS experiment, MS raw files were processed using Proteome Discoverer 2.5 (Thermo Scientific Inc.) and Minora Feature Detector algorithm in Proteome Discoverer. Mascot Distiller and Mascot Server (v 2.5, Matrix sciences) were utilized to produce fragment ion spectra and to perform the database searches. Peptide Validator and Protein FDR Validator nodes in Proteome Discoverer were used for data annotation. For the LC-MS/MS experiment (comparative LEAP-RBP experiment), MS raw files were processed using Proteome Discoverer 3.0 (Thermo Scientific Inc.) and Minora Feature Detector algorithm in Proteome Discoverer. Sequest with INFERYS in Proteome Discoverer was utilized to produce fragment ion spectra and perform database searches. Peptide Validator and Protein FDR Validator nodes in Proteome Discoverer were used for data annotation.<br><br>The following software were used for small RNA sequencing experiments:<br>Reads were cleaned using custom perl and python scripts which are proprietary software of Novogene. Reads were mapped and expression levels were analyzed using Bowtie version 0.12.9 [https://sourceforge.net/projects/bowtie-bio/files/bowtie/0.12.9/] (Langmead et al., 2019). Repeat analysis was performed using RepeatMasker version 4.0.3 [https://www.repeatmasker.org/] (Smit et al., 2013-2015), and Rfam version 11.0 [https://rfam.xfam.org/] (Burge et al., 2013).

Mapped small RNAs were examined for known miRNA homologies using miRDeep2 version 0.0.5 [https://github.com/rajewsky-lab/mirdeep2] (Friedlander et al., 2011).
Novel miRNA predictions were performed using miRDeep2 version 0.0.5 modified with with miREvo version 1.1 [https://github.com/akahanaton/miREvo] (Wen et al., 2012), and ViennaRNA version 2.1.1 [https://www.tbi.univie.ac.at/RNA/#download] (Lorenz et al., 2011).

For manuscripts utilizing custom algorithms or software that are central to the research but not yet described in published literature, software must be made available to editors and reviewers. We strongly encourage code deposition in a community repository (e.g. GitHub). See the Nature Portfolio guidelines for submitting code & software for further information.

Data

Policy information about availability of data

All manuscripts must include a data availability statement. This statement should provide the following information, where applicable:

- Accession codes, unique identifiers, or web links for publicly available datasets
- A description of any restrictions on data availability
- For clinical datasets or third party data, please ensure that the statement adheres to our policy

A data availability statement is provided in the manuscript and included here as well, sans embedded references. Raw data and Protein Discoverer results files from LEAPR-RBP and INP SILAC, and non-SILAC LC-MS/MS experiments are available on the MassIVE repository, accession record MSV000088005: [https://massive.ucsd.edu/ProteoSAFe/dataset.jsp?accession=MSV000088005]. Small RNA sequencing data are available at NCBI GEO, series record GSE235647 [https://www.ncbi.nlm.nih.gov/geo/query/acc.cgi?acc=GSE235647]. Maxquant output files for XRNAX, OOPs, Ptex, and TRAPP were downloaded from the ProteomeXchange using the following accession codes; XRNAX: PXD010520 [https://www.ebi.ac.uk/pride/archive/projects/PXD010520] (proteinGroups.txt file located in the txt_ihRBP.zip file); OOPs: PXD021169 [https://www.ebi.ac.uk/pride/archive/projects/PXD021169] (proteinGroups.txt file located in the txt.zip file); Ptex: PXD009571 [https://www.ebi.ac.uk/pride/archive/projects/PXD009571] (proteinGroups.txt file located in the txt_Human.zip file); TRAPP: PXD011071 [https://www.ebi.ac.uk/pride/archive/projects/PXD011071] (Maxquant_proteinGroups.txt files located in the TRAPP_cerevisiae_400.zip, TRAPP_cerevisiae_800.zip, and TRAPP_cerevisiae_1360.zip files). MS datasets for RIC and eRIC including protein identifiers, unique peptide counts, and sum peptide intensities were obtained from [https://www.nature.com/articles/s41467-018-06557-8] (Supplementary Data 1, “full dataset” tab) [52].

Complete MS datasets and parameters discussed in this study for LEAP-RBP, INP, and each of the referenced studies are provided as individual Excel files: LEAP-RBP (Supplementary Data 3), INP (Supplementary Data 5), XRNAX (Supplementary Data 10), OOPs (Supplementary Data 11), Ptex 1.5 (Supplementary Data 12), TRAPP 1360 (Supplementary Data 13), RIC (Supplementary Data 14), eRIC (Supplementary Data 15), TRAPP 400 (Supplementary Data 16), TRAPP 800 (Supplementary Data 17), Ptex 0.015 (Supplementary Data 18), Ptex 0.15 (Supplementary Data 19), and OOPs LFQ (Supplementary Data 20). Data sources for all referenced MS datasets can be found in Supplementary Data 6. Summary of statistical tests used for identification of UV-enriched* protein IDs in LEAP-RBP and INP fractions by SILAC LC-MS/MS were provided in Supplementary Data 2, 4, respectively. Summary of statistical tests used for identification of proteins displaying a significant difference in total (input samples) and/or RNA-bound abundance (clRNP fractions) in response to harringtonine treatment were provided in Supplementary Data 8, 9, respectively.

The data supporting the findings of this study are available within the main Manuscript and Supplementary Information, or in the Source data provided with this paper. Specific p values are included within the Source Data file as well.

Research involving human participants, their data, or biological material

Policy information about studies with human participants or human data. See also policy information about sex, gender (identity/presentation), and sexual orientation and race, ethnicity and racism.

Reporting on sex and gender	N/A
Reporting on race, ethnicity, or other socially relevant groupings	N/A
Population characteristics	N/A
Recruitment	N/A
Ethics oversight	N/A

Note that full information on the approval of the study protocol must also be provided in the manuscript.

Field-specific reporting

Please select the one below that is the best fit for your research. If you are not sure, read the appropriate sections before making your selection.

[x] Life sciences [ ] Behavioural & social sciences [ ] Ecological, evolutionary & environmental sciences

For a reference copy of the document with all sections, see nature.com/documents/nr-reporting-summary-flat.pdf

Life sciences study design

All studies must disclose on these points even when the disclosure is negative.

Sample size	Experiments were performed with three biologically independent samples per sample group when testing for a difference in RNA and protein yield between experimental groups. Experiments were performed with two independently prepared samples per sample group when testing for main effects using multiple (>3) experimental groups and/or when biological variability was not of interest. No sample size calculations were performed. Sample sizes were chosen based on the minimal number of samples required to perform the desired statistical tests.
Data exclusions	No data were excluded.
Replication	The number of times experiments were repeated is included in the Figure and Supplementary Figure legends. Results were further validated using orthogonal approaches.
Randomization	Randomization was not applicable as this study did not involve patients or clinical trials. SILAC LC-MS/MS experiments were performed without label swapping and potential biases in labeling efficiency were ruled out based on the comparable recovery and abundance of background proteins.
Blinding	Blinding was not relevant to this study as no investigator-dependent selection of data was performed.

Reporting for specific materials, systems and methods

We require information from authors about some types of materials, experimental systems and methods used in many studies. Here, indicate whether each material, system or method listed is relevant to your study. If you are not sure if a list item applies to your research, read the appropriate section before selecting a response.

Materials & experimental systems

n/a	Involved in the study	
☐	☒	Antibodies
☐	☒	Eukaryotic cell lines
☐	☐	Palaeontology and archaeology
☐	☐	Animals and other organisms
☐	☐	Clinical data
☐	☐	Dual use research of concern
☐	☐	Plants

Methods

n/a	Involved in the study	
☒	☐	ChIP-seq
☒	☐	Flow cytometry
☒	☐	MRI-based neuroimaging

Antibodies

Antibodies used	Anti-pABPC1 (A14872, ABclonal); anti-PABPC4 (A5948, ABclonal); anti-TIA1 (A6237, ABclonal); anti-HuR (Sc-5261, Santa Cruz Biotechnology); anti-XRN1 (A300-443A, Bethyl Laboratories); anti-RPL4 (Sc-100838, Santa Cruz Biotechnology); Anti-RPL8 (A10042, ABclonal); anti-LRRC59 (A305-076A, Bethyl Laboratories); anti-NCL (A5904, ABclonal); anti-RPN1 (alphaP3, Nicchitta); anti-TRAPalpha (TRAPalpha, Nicchitta); anti-GRP94 (DU120, Nicchitta); anti-GAPDH (DSHB-hGAPDH-2G7, DSHB); GRP78 (Sc-376768, Santa Cruz Biotechnology); anti-beta-tubulin (E7-s, DSHB); anti-RPS3 (A4872, ABclonal); anti-SND1 (A5874, ABclonal); anti-UPF1/RENT1 (A5071, ABclonal); anti-HDLBP (A20896, ABclonal); anti-ABCF3 (A15168, ABclonal); anti-GEMIN5 (A17125, ABclonal); anti-eEF2 (A9721, ABclonal); anti-CELF1 (A5958, ABclonal); anti-Fibrillarin/U3 RNP (A1136, ABclonal). Detailed information on antibodies used and immunoblotting conditions is provided in the Methods and Supplementary Data 1. Full blots for each target protein are provided in Supplementary Figure 1.
Validation	Validation for each antibody can be found using the following URL links.<br>Anti-pABPC1: https://abclonal.com/catalog-antibodies/pAbPC1RabbitpAb/A14872.<br>Anti-PABPC4: https://abclonal.com/catalog-antibodies/PABPC4RabbitpAb/A5948.<br>Anti-TIA1: https://abclonal.com/catalog-antibodies/TIA1PolyclonalAntibody/A6237.<br>Anti-HuR: https://www.scbt.com/p/hurantibody-3a2.<br>Anti-XRN1: https://www.thermofisher.com/antibody/product/XRN1-Antibody-Polyclonal/A300-443A.<br>Anti-RPL4: https://www.scbt.com/p/ribosomal-protein-l4-antibody-rq-7?requestFrom=search.<br>Anti-RPL8: https://abclonal.com/catalogantibodies/ RPL8RabbitpAb/A10042.<br>Anti-LRRC59: https://www.thermofisher.com/antibody/product/LRRC59-Antibody-Polyclonal/A305-076A.<br>Anti-NCL: https://abclonal.com/catalog-antibodies/NCLRabbitpAb/A5904.<br>Anti-RPN1 and anti-TRAPalpha: https://www.jbc.org/article/S0021-9258(20)31543-X/fulltext.<br>Anti-GRP94: https://www.sciencedirect.com/science/ article/pii/S0021925819664604.<br>Anti-GAPDH: https://dshb.biology.uiowa.edu/DSHB-hGAPDH-2G7.<br>Anti-GRP78: https://datasheets.scbt.com/sc-376768.pdf.<br>Anti-beta-tubulin: https://dshb.biology.uiowa.edu/E7_2.<br>Anti-RPS3: https://abclonal.com/catalog-antibodies/RPS3RabbitmAb/A4872.<br>Anti-SND1: https://abclonal.com/catalog-antibodies/KOValidatedSND1RabbitpAb/A5874.

Anti-UPF1: https://abclonal.com/catalog-antibodies/UPF1RENT1RabbitmAb/A5071.
Anti-HDLBP: https://abclonal.com/catalog-antibodies/HDLBPRabbitmAb/A20896.
Anti-ABCF3: https://abclonal.com/catalog-antibodies/ABCF3RabbitpAb/A15168.
Anti-GEMIN5: https://abclonal.com/catalog-antibodies/GEMIN5RabbitpAb/A17125.
Anti-eEF2: https://abclonal.com/catalog-antibodies/eEF2RabbitmAb/A9721.
Anti-CELF1: https://abclonal.com/catalog-antibodies/CELF1RabbitpAb/A5958.
Anti-FBL: https://abclonal.com/catalog-antibodies/FibrillarinU3RNPRabbitpAb/A1136.

Validation was performed by siRNA knockdown and immunoblotting, as well as SDS-PAGE mobility assays, either on site or as supplied by the vendors noted above.

Eukaryotic cell lines

Policy information about cell lines and Sex and Gender in Research

Cell line source(s)	Immortalized human-derived cell lines (HeLa, 293T, and HuH7) were obtained from the Duke University Comprehensive Cancer Center Cell Culture facility and screened for mycoplasma contamination by RT-PCR analysis. Rat insulinoma cell line 832/13 was provided by Christopher Newgard, PhD, Duke University School of Medicine.
Authentication	Cell line authentication performed by the Duke University Comprehensive Cancer Center Cell Culture Facility by STR analysis.
Mycoplasma contamination	All cell lines were screened for mycoplasma contamination by RT-PCR using the ThermoFisher MycoSEQ Mycoplasma Detection System and tested negative.
Commonly misidentified lines (See ICLAC register)	No commonly misidentified cell lines were used in this study.

Palaeontology and Archaeology

Specimen provenance	N/A
Specimen deposition	N/A
Dating methods	N/A

☐ Tick this box to confirm that the raw and calibrated dates are available in the paper or in Supplementary Information.

Ethics oversight	N/A

Note that full information on the approval of the study protocol must also be provided in the manuscript.

Animals and other research organisms

Policy information about studies involving animals; ARRIVE guidelines recommended for reporting animal research, and Sex and Gender in Research

Laboratory animals	N/A
Wild animals	N/A
Reporting on sex	N/A
Field-collected samples	N/A
Ethics oversight	N/A

Note that full information on the approval of the study protocol must also be provided in the manuscript.

Clinical data

Policy information about clinical studies
All manuscripts should comply with the ICMJEguidelines for publication of clinical research and a completedCONSORT checklist must be included with all submissions.

Clinical trial registration	N/A
Study protocol	N/A

Data collection	N/A
Outcomes	N/A

Dual use research of concern

Policy information about dual use research of concern

Hazards

Could the accidental, deliberate or reckless misuse of agents or technologies generated in the work, or the application of information presented in the manuscript, pose a threat to:

No	Yes	
x		Public health
x		National security
x		Crops and/or livestock
x		Ecosystems
x		Any other significant area

Experiments of concern

Does the work involve any of these experiments of concern:

No	Yes	
x		Demonstrate how to render a vaccine ineffective
x		Confer resistance to therapeutically useful antibiotics or antiviral agents
x		Enhance the virulence of a pathogen or render a nonpathogen virulent
x		Increase transmissibility of a pathogen
x		Alter the host range of a pathogen
x		Enable evasion of diagnostic/detection modalities
x		Enable the weaponization of a biological agent or toxin
x		Any other potentially harmful combination of experiments and agents
