# Supplementary material for: Signal-noise metrics for RNA binding protein identification reveal broad spectrum protein-RNA interaction frequencies and dynamics
Source: Nat Commun. 2023 Sep 21;14:5868. doi: 10.1038/s41467-023-41284-9 (PMC10514315; doi:10.1038/s41467-023-41284-9)
Supplement: Supplementary file 4 — Description of Additional Supplementary Files [file 41467_2023_41284_MOESM4_ESM.pdf]

Title: Supplementary Data 1
Description: Western blot conditions and protein target information.

Title: Supplementary Data 2
Description: Summary of statistical tests used for identification of UV-enriched* protein IDs in LEAP-RBP fractions by SILAC LC-MS/MS analysis.

Title: Supplementary Data 3
Description: Complete MS dataset for SILAC LC-MS/MS analysis of LEAP-RBP fractions.

Title: Supplementary Data 4
Description: Summary of statistical tests used for identification of UV-enriched* protein IDs in INP fractions by SILAC LC-MS/MS analysis.

Title: Supplementary Data 5
Description: Complete MS dataset for SILAC LC-MS/MS analysis of INP fractions.

Title: Supplementary Data 6
Description: Primary UniProt ID formatting and sources for referenced MS datasets.

Title: Supplementary Data 7
Description: Category and GO terms.

Title: Supplementary Data 8
Description: Complete MS dataset for LC-MS/MS analysis of input samples during the comparative LEAP-RBP experiment. Includes a summary of the statistical tests used for the identification of proteins displaying a significant difference in total abundance in response to harringtonine treatment.

Title: Supplementary Data 9
Description: Complete MS dataset for LC-MS/MS analysis of clRNP fractions during the comparative LEAP-RBP experiment. Includes a summary of the statistical tests used for the identification of proteins displaying a significant difference in RNA-bound abundance in response to harringtonine treatment.

Title: Supplementary Data 10
Description: Complete MS dataset for referenced XRNAX study (Trendel, 2019).

Title: Supplementary Data 11
Description: Complete MS dataset for referenced OOPs study (Hoefig, 2021).

Title: Supplementary Data 12
Description: Complete MS dataset for referenced Ptex study, 1.5 J/cm^2 (Urdaneta, 2019).

Title: Supplementary Data 13
Description: Complete MS dataset for referenced TRAPP study, 1,360 mJ/cm^2 (Shchepachev, 2019).

Title: Supplementary Data 14
Description: Complete MS dataset for referenced RIC study (Perez-Perri, 2018).

Title: Supplementary Data 15
Description: Complete MS dataset for referenced eRIC study (Perez-Perri, 2018).

Title: Supplementary Data 16
Description: Complete MS dataset for referenced TRAPP study, 400 mJ/cm^2 (Shchepachev, 2019).

Title: Supplementary Data 17
Description: Complete MS dataset for referenced TRAPP study, 800 mJ/cm^2 (Shchepachev, 2019).

Title: Supplementary Data 18
Description: Complete MS dataset for referenced Ptex study, 0.15 J/cm^2 (Urdaneta, 2019).

Title: Supplementary Data 19
Description: Complete MS dataset for referenced Ptex study, 0.015 J/cm^2 (Urdaneta, 2019).

Title: Supplementary Data 20
Description: Complete MS dataset for referenced OOPs study, LFQ (Hoefig, 2021).
